# Cyclodextrins as Modulators of Photostability and Photochemical Behavior: Mechanisms, Analytical Assessment, and Applications

**DOI:** 10.3390/ijms272110040

**Published:** 2026-11-09

**Authors:** Monika Zielińska-Pisklak, Agnieszka Mykytyn, Marcin Gackowski, Łukasz Szeleszczuk

**Affiliations:** 1 Department of Pharmaceutical Chemistry and Biomaterials, Faculty of Pharmacy, Medical University of Warsaw, 1 Banacha Str., 02-093 Warsaw, Poland; 2 Department of Organic and Physical Chemistry, Medical University of Warsaw, 1 Banacha Str., 02-097 Warsaw, Poland; s091821@student.wum.edu.pl (A.M.); lukasz.szeleszczuk@wum.edu.pl (Ł.S.); 3 Department of Toxicology and Bromatology, Faculty of Pharmacy, L. Rydygier Collegium Medicum in Bydgoszcz, Nicolaus Copernicus University in Torun, 2 Jurasza Str., 85-089 Bydgoszcz, Poland; marcin.gackowski@cm.umk.pl

**Keywords:** cyclodextrins, photostability, photodegradation, inclusion complexes, host–guest interactions, photoprotection, excited-state processes, photoresponsive systems, supramolecular chemistry

## Abstract

Photostability is a critical determinant of the performance and safety of light-sensitive compounds, yet the effects of cyclodextrin (CD) complexation are often interpreted as uniformly protective. This review critically examines how native and modified CDs and CD-based supramolecular architectures influence photodegradation and other light-driven processes across pharmaceuticals, natural bioactives, vitamins, sunscreen filters, dyes, photosensitizers, and agrochemicals. Evidence from experimental studies is evaluated in relation to host–guest inclusion geometry, conformational restriction, aggregation, microenvironmental polarity, oxygen accessibility, and excited-state and radical pathways. Direct quantification of intact compounds by stability-indicating chromatography (e.g., HPLC/UPLC) is distinguished from indirect indicators such as spectral changes or retained functional activity. The literature reveals condition-dependent outcomes ranging from substantial photoprotection to negligible effects or accelerated photodegradation, potentially reflecting disruption of protective guest aggregates or differential shielding of hydrolysis- and photolysis-prone regions. We also discuss photoresponsive systems in which CD association is exploited to control, rather than suppress, photochemical reactions. Reliable assessment requires matched irradiation controls, characterized irradiation spectra and doses (using ICH Q1B benchmarks where applicable), and consideration of formulation effects. Overall, cyclodextrins emerge as context-dependent modulators of photochemical behavior, supporting a shift from empirical complexation toward mechanism-guided design of light-stable and photoactive systems.

## 1. Introduction

Light-induced degradation is a major stability concern for a wide range of biologically and technologically relevant compounds. Absorption of ultraviolet or visible radiation may generate electronically excited states from which molecules undergo photoisomerization, bond cleavage, oxidation, electron or energy transfer, radical reactions, or processes involving molecular oxygen and reactive oxygen species. The consequences may extend beyond simple loss of the parent compound and include changes in physicochemical properties, biological activity, and formulation performance, as well as the formation of photoproducts with distinct properties [1,2]. In pharmaceuticals, these changes may affect potency and safety during storage or use, whereas in other applications they may impair the intended function of the light-sensitive compound [3,4,5,6].

This problem occurs across chemically and functionally diverse classes of compounds. Retinoids and vitamin A derivatives readily undergo light-induced isomerization and oxidation, whereas many natural phenolic compounds combine limited aqueous solubility with susceptibility to light and oxygen [7,8]. For organic sunscreen filters, photostability is intrinsically linked to performance because degradation or isomerization may alter the spectral properties of the filter and generate reactive intermediates or photoproducts [9,10]. Agrochemicals may encounter similar processes after environmental exposure, while in fluorescent dyes and imaging probes repeated excitation can lead to aggregation-dependent quenching, non-radiative deactivation and photobleaching [11,12]. Photostability is therefore not a problem confined to one type of formulation, but a common challenge across pharmaceutical, food, cosmetic, analytical and environmental applications. In other systems, however, light-driven processes are deliberately exploited for functions such as photosensitization [13,14], molecular switching [15], controlled release [16,17], or optical sensing [18,19].

Cyclodextrins (CDs) provide an unusual approach to this problem because they act through molecular recognition and modification of the local environment of the guest. Native α-, β- and γ-CDs, as well as numerous chemically modified derivatives, possess a relatively hydrophobic internal cavity surrounded by a hydrophilic exterior and can accommodate entire guest molecules or selected molecular regions [20,21,22]. Inclusion can improve aqueous solubility and chemical stability and may reduce susceptibility to light-induced degradation, while substitution of the CD provides further control over affinity, solubility, charge and supramolecular organization [20,21,23]. This versatility has led to the use of CDs not only as pharmaceutical excipients but also as components of polymers, nanocarriers, cross-linked networks and increasingly complex supramolecular materials [20,21,24].

An important aspect of cyclodextrin functionality is their influence on photostability, although this effect is not straightforward. CDs generally do not act simply by preventing the guest from absorbing UV or visible radiation; instead, complexation may modify the fate of the excited molecule and the pathways available after excitation [25]. Depending on the system, cyclodextrin complexation may restrict molecular motion, alter aggregation or the accessibility of reactive species, or modify the photochemical pathways available after excitation. Importantly, these effects are not always protective. This variability is evident across different guest–CD systems: closely related curcuminoids can respond differently to the same CD environment, different CDs can produce opposite effects for the same guest, and stabilization against one degradation pathway may coexist with enhanced degradation through another [26,27,28,29]. Thus, evidence of inclusion-complex formation alone is insufficient to predict the photochemical outcome.

This variability in photochemical response is also becoming increasingly relevant as CD-based systems move beyond conventional binary inclusion complexes. Cross-linked networks, nanosponges, liposomal combinations, covalent conjugates and mechanically interlocked structures can provide more persistent or spatially defined guest confinement, whereas photoresponsive host–guest systems deliberately use light to control supramolecular assembly, release or molecular reactivity. In such materials, cyclodextrins may serve not only to suppress unwanted photochemical degradation, but also to enable or regulate useful light-driven processes [17,30,31,32,33,34]. The distinction between photoprotection and controlled photoactivity is becoming increasingly important as the structural complexity and functional scope of CD-based systems expand.

Previous reviews have addressed substantial parts of this field from different perspectives. Pharmaceutical photostability has been considered in the context of degradation mechanisms, formulation strategies and regulatory testing, with cyclodextrins discussed among several approaches to stabilization [1,2,35]. More focused reviews have examined CD-mediated photoprotection, photosensitive 1,4-dihydropyridines, sunscreen filters, topical formulations and broader effects of CDs on drug stability [9,36,37,38]. In parallel, supramolecular and photoactive CD systems have been reviewed in relation to excited-state processes, aggregation, photosensitization and light-responsive behavior [39,40,41]. These contributions are summarized in Supplementary Table S1.

Despite this considerable body of work, a broader analysis linking molecular determinants of CD-mediated photostability with the quality of the experimental evidence across different application areas remains limited. Studies differ markedly in irradiation source and dose, physical state, formulation composition, analytical endpoint, and the controls used to distinguish a genuine CD effect from other sources of stabilization [4,42]. A particular concern is the absence of parallel dark controls in some published studies. Without otherwise identical, temperature-matched non-irradiated samples, degradation caused by thermal processes, hydrolysis, or other solvent-mediated reactions may be incorrectly attributed to light exposure, leading to an overestimation or misinterpretation of photodegradation. Consequently, reported photoprotective effects are not always directly comparable across studies. Moreover, absent or adverse effects are informative, as they help identify conditions in which cyclodextrin complexation provides little or no protection. Spectroscopic changes, retained biological activity, and formulation-level stability may all provide useful information, but they do not provide the same level of evidence as matched irradiation experiments that quantitatively compare the intact guest in free and CD-associated systems [43]. These considerations formed the basis for the inclusion criteria and the subsequent classification of studies in this review.

The relevant literature was identified through searches of Web of Science, Scopus, and Google Scholar. The searches were updated through September 2026. Search strategies combined terms related to cyclodextrins and their derivatives with terms describing light-induced processes, including photostability, photodegradation, photolysis, UV, visible light, photoisomerization, photobleaching, photosensitization, photoresponsive, and photodynamic behavior. Reference lists of relevant primary studies and previous reviews were also screened to identify additional publications. Original studies were considered when they investigated native or modified cyclodextrins, CD-based polymers or networks, covalent CD conjugates, or supramolecular CD architectures and provided experimental evidence relevant to guest stability or mechanistic insight into photochemical behavior under irradiation. Studies concerned exclusively with solubility, complex formation, or non-photochemical stability were excluded unless they provided information directly supporting the interpretation of a light-induced process.

Studies were classified according to both application area and evidential scope. Studies reporting photostabilization are summarized in Tables 1–5. Representative cases of absent, adverse, marginal or strongly condition-dependent effects are highlighted in Table 6, while a more comprehensive compilation is provided in Supplementary Table S2, whereas studies in which photostability was assessed indirectly or the specific contribution of cyclodextrin could not be isolated with sufficient confidence are summarized in Supplementary Table S3. Photoactive and photoresponsive systems in which light is deliberately used to control molecular or supramolecular function are presented separately in Supplementary Table S4.

The present review considers cyclodextrins not simply as photoprotective carriers, but more broadly as modulators of photostability and photochemical behavior. Experimental evidence from pharmaceuticals, natural bioactives, nutraceuticals and vitamins, sunscreen filters, dyes and photosensitizers, and agrochemicals is examined in relation to guest structure, cyclodextrin type, guest–CD geometry, molecular environment, physical state and formulation architecture. Attention is given to the distinction between direct evidence of photostability, absent or adverse effects, and studies in which photostability was assessed indirectly or the specific contribution of CD complexation could not be clearly distinguished from other effects. Photoactive and photoresponsive systems are considered separately to distinguish suppression of unwanted photodegradation from the deliberate use of light to control molecular or supramolecular function. The aim is to identify the conditions that favor cyclodextrin-mediated photoprotection, to understand why this effect may be weak, absent, or reversed in other systems, and to define the experimental evidence required for its reliable assessment.

## 2. Molecular Mechanisms Underlying Cyclodextrin Effects on Photostability

Cyclodextrin-mediated photostabilization is commonly described in terms of encapsulation or molecular shielding, but these concepts alone do not fully explain the observed photochemical effects. Complexation does not necessarily prevent the guest from absorbing UV or visible radiation. Instead, when protection occurs, it may result from changes in the molecular environment and in the reaction pathways available after excitation [44,45,46]. For avobenzone, the characteristic UVA absorption is retained in the presence of HP-β-CD despite a marked reduction in photodegradation [25]. Cyclodextrins should therefore be regarded mainly as modifiers of processes occurring after light absorption, although steric or conformational restriction may also contribute when the photolabile region of the guest is favorably positioned within the cavity [47,48].

**Figure 1:**
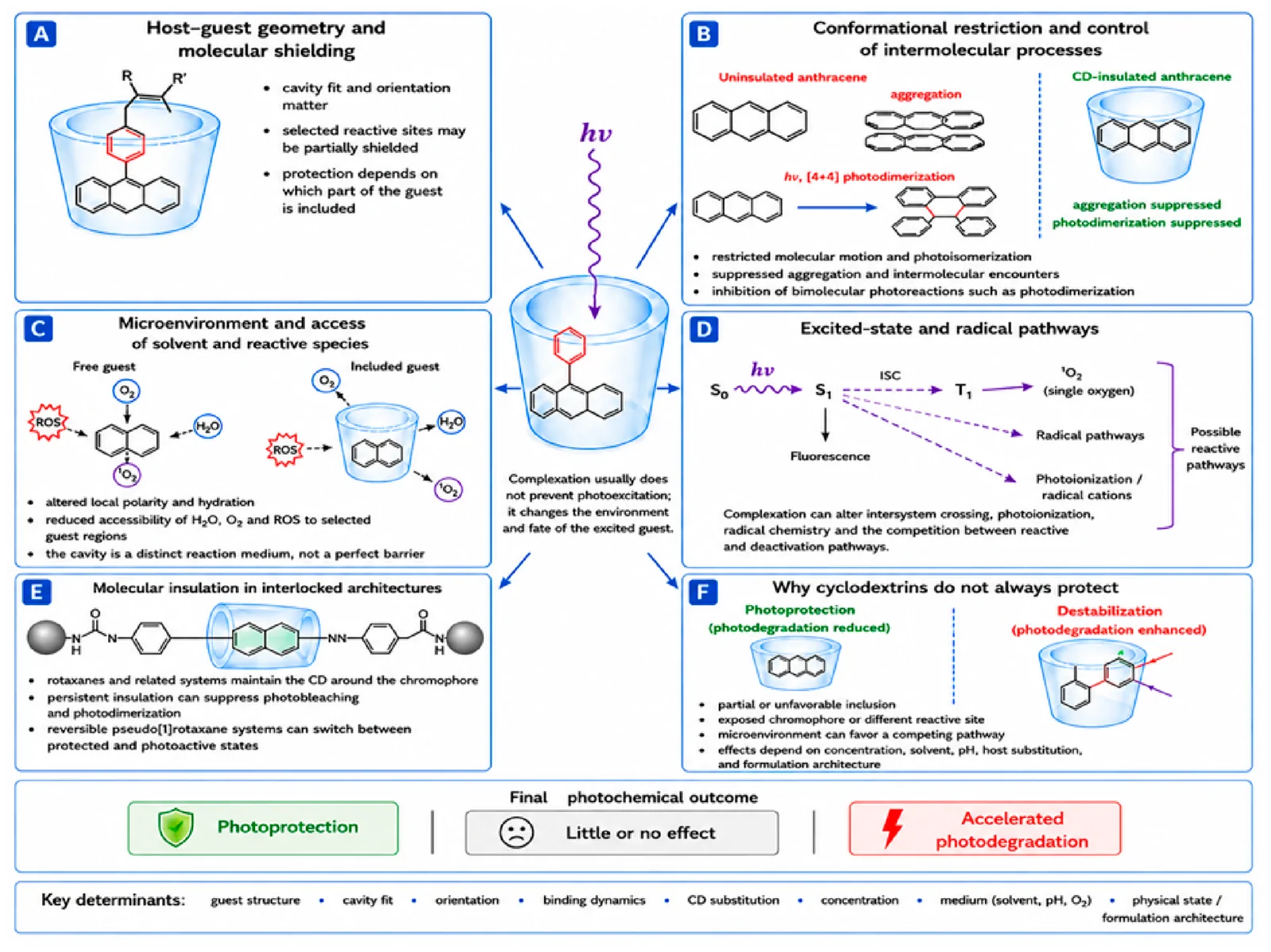
Main mechanisms by which cyclodextrins modify the photochemical behavior of a guest. Depending on guest structure, inclusion geometry, host properties and the surrounding environment, complexation may reduce, leave unchanged or increase photodegradation.

These post-absorption effects may arise through several, often concurrent, mechanisms. Inclusion may restrict molecular motion, alter aggregation and intermolecular contact, change local polarity and hydration, modify the accessibility of solvent, molecular oxygen and reactive species, or affect excited-state and radical pathways. The relative importance of these effects depends on the structure of the guest, the cavity size and substitution pattern of the host, the geometry and dynamics of the complex, and the surrounding medium [12,44,45,48,49,50,51]. These effects do not necessarily result in photoprotection, as a microenvironment that suppresses one degradation pathway may have little influence on another or may promote an alternative reaction [28,50]. The observed photostability therefore depends on the combined influence of the guest, the cyclodextrin, and the surrounding environment rather than on an intrinsic photoprotective property of the host. The main mechanisms involved are summarized in Figure 1.

### 2.1. Host–Guest Geometry, Cavity Fit, and Molecular Shielding

The extent of cyclodextrin-mediated photoprotection depends strongly on the geometry of the host–guest complex and on the position of the photolabile region relative to the CD cavity. Many pharmaceutical and bioactive guests are only partially accommodated within the host, so complex formation does not necessarily isolate the entire chromophore or reactive group from the surrounding medium. A stable complex may therefore leave the photolabile bond exposed to the surrounding medium or accessible to reactive partners [28,47]. This dependence on guest geometry is illustrated by imidazoline-derived drugs complexed with β-CD: photostability increased markedly for naphazoline and xylometazoline, whereas the protective effect was much less pronounced for antazoline, whose larger molecular structure was predicted to leave a larger part of the molecule outside the cavity [52].

In the isotretinoin/HP-β-CD system, molecular modeling indicated that the extended retinoid chain, including the region involved in light-induced isomerization, was at least partly included within the CD cavity. Using Lineweaver–Burk analysis, Yap et al. [47] estimated a limiting degradation rate constant (k_c_) of 0.0612 h^−1^ for the fully complexed 13-cis-RA, compared with 0.4286 h^−1^ for the uncomplexed drug (k_free_). Importantly, the approximately sevenfold difference refers to the estimated limiting rate constants rather than to the degradation rates necessarily observed at a given CD concentration. Assuming rapid 1:1 complexation equilibrium and pseudo-first-order degradation kinetics, the observed rate constant can be expressed as k_obs_ = f_free_ × k_free_ + f_bound_ × k_c_, where f_free_ and f_bound_ represent the fractions of uncomplexed and complexed drug, respectively (f_free_ + f_bound_ = 1). Consequently, the actual photoprotective effect depends on the extent of complexation under the experimental conditions. The authors attributed the improved photostability to steric restriction of the photolabile chain imposed by the CD cavity. This example illustrates why guest orientation, fractional cavity occupancy and the position of the photolabile region should be considered together with binding strength.

Cavity size is important, but a larger cavity does not necessarily provide better protection. Karpkird et al. investigated covalently modified cyclodextrins bearing methoxycinnamoyl chromophores and found that photostability improved when the chromophore was appropriately matched to the CD cavity [53]. Spectroscopic and ROESY data provided evidence of differences in self-inclusion among the conjugates, suggesting that the extent and geometry of chromophore accommodation, rather than cavity size alone, were important factors determining the protective effect.

The chemical nature of the host can also change the outcome for the same guest. Differences in the degree of photoprotection among β-CD, HP-β-CD and DM-β-CD complexes of nifedipine show that host identity and substitution can influence the photochemical outcome [49]. Substitution can alter not only aqueous solubility but also cavity shape, steric accessibility and electrostatic interactions at the cavity rims. Consequently, derivatives of the same parent cyclodextrin may create different environments around the included chromophore [50]. These differences also indicate that host performance cannot be predicted from binding strength alone.

Binding affinity should also be interpreted with caution in relation to photostability. A high association constant increases the fraction of guests present in the complexed state, but it does not indicate whether that state is photochemically favorable. The example of curcumin illustrates this distinction clearly. In the study conducted by Tønnesen et al., several cyclodextrins markedly increased its aqueous solubility and showed substantial affinity for the guest, yet these interactions did not translate into improved photostability under the tested conditions [50]. Binding strength determines how much of the guest is present in the cyclodextrin environment, whereas the properties of that environment determine its photochemical effect.

### 2.2. Conformational Restriction and Control of Intermolecular Processes

Cyclodextrin complexation can influence photostability by restricting molecular motions involved in excited-state transformation. This is relevant for molecules undergoing twisting, cis-trans isomerization, internal rotation or other conformational rearrangements following excitation. Confinement within the host cavity can restrict these motions and thereby shift the balance between radiative relaxation, non-radiative decay and photochemical reaction [12,47,48]. For example, reduced conformational freedom of the cyanine chromophore in an α-CD-insulated rotaxane was associated with enhanced fluorescence and photostability, whereas β-CD confinement of IAD dyes suppressed intramolecular motion and non-radiative relaxation [12,48].

**Figure 2:**
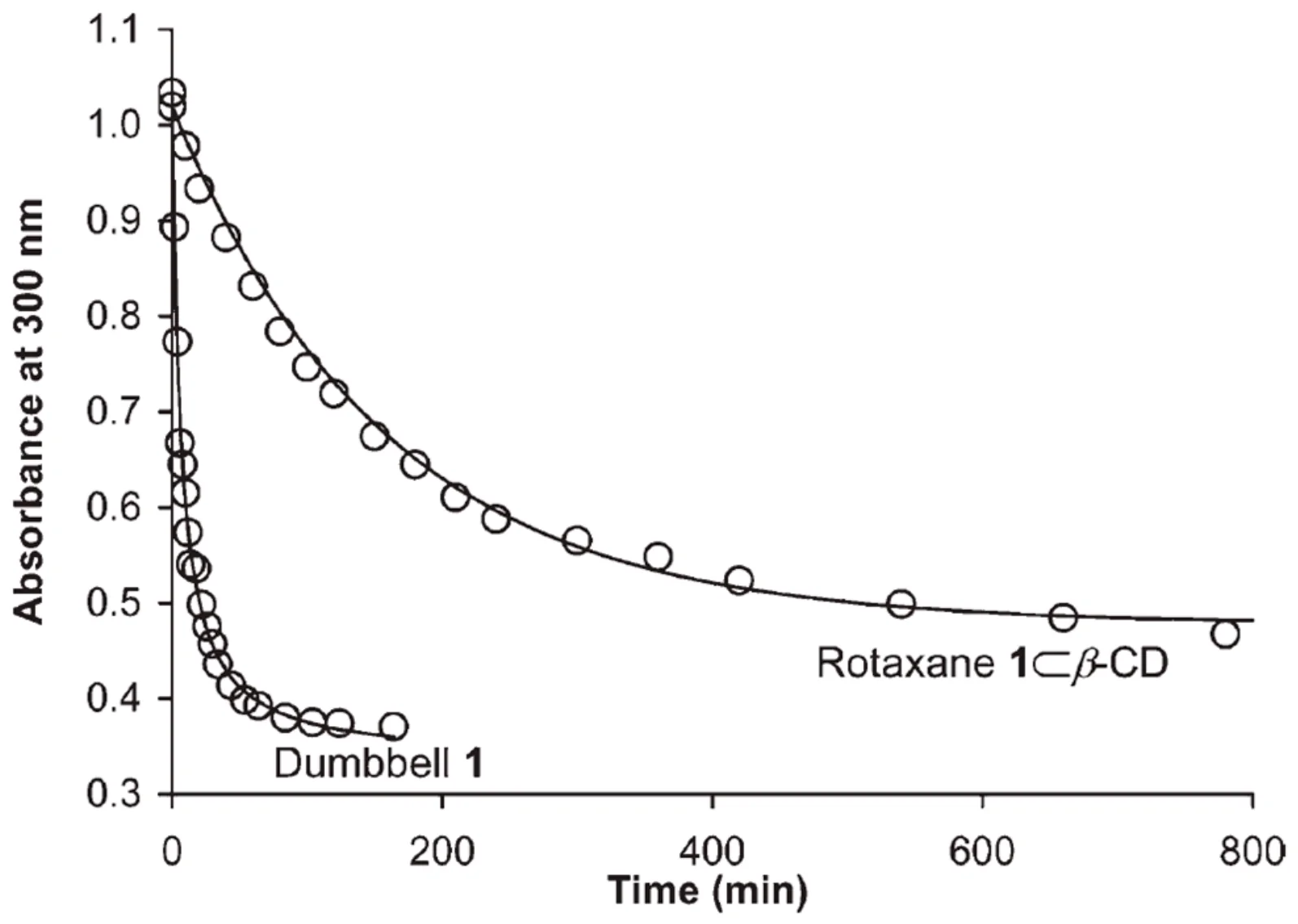
Photobleaching of the β-CD-insulated anthracene rotaxane and its uninsulated dumbbell analogue during irradiation at 380 nm. “Dumbbell 1” denotes the anthracene-containing molecular axle with bulky stopper groups but without β-CD, whereas in rotaxane 1⊂β-CD the cyclodextrin is mechanically retained around the anthracene moiety. The reported photobleaching half-lives were 109 ± 4 min for the β-CD-insulated rotaxane (pseudo-first-order fit) and 13 ± 0.4 min for the uninsulated dumbbell (second-order fit), demonstrating substantially greater photobleaching resistance upon mechanical insulation. Reproduced, with permission, from Stone and Anderson (2007) [54].

Photostability may also depend on intermolecular organization. For many chromophores, intermolecular processes also contribute to photodegradation. Aggregation, excitonic interactions and photodimerization may become important when molecules approach one another in an excited state. By spatially separating chromophores or restricting their relative orientation, cyclodextrin inclusion can reduce such interactions and modify both photophysical behavior and photoproduct formation [12,43,54]. In the β-CD-insulated anthracene rotaxane reported by Stone and Anderson, mechanical isolation strongly altered the intermolecular behavior of the chromophore. The uninsulated anthracene derivative showed concentration-dependent spectral changes consistent with aggregation, whereas these effects were largely absent in the mechanically interlocked system. Under 380 nm irradiation, the uninsulated anthracene dumbbell exhibited a photobleaching half-life of 13 ± 0.4 min, whereas the β-CD-insulated rotaxane showed a substantially longer half-life of 109 ± 4 min [54]. Importantly, these values were obtained using different kinetic models: second-order kinetics for the uninsulated dumbbell and pseudo-first-order kinetics for the rotaxane, consistent with the suppression of intermolecular photodimerization upon mechanical insulation. The difference in photobleaching behavior between the rotaxane and its uninsulated anthracene analogue is shown in Figure 2. At low concentrations, however, the two compounds showed similar fluorescence quantum yields, suggesting that the improved photostability was mainly related to reduced intermolecular interactions and photodimerization rather than to increased fluorescence efficiency. Mechanically interlocked structures therefore provide a more fixed form of CD-mediated insulation, in which the cyclodextrin remains positioned around the chromophore rather than continuously associating and dissociating as in conventional inclusion complexes [48,54].

Comparable effects can also occur in conventional inclusion complexes. Orfanou and co-workers showed that oxolinic acid became progressively less photostable as its initial concentration increased, while NMR measurements indicated self-association in solution. HP-β-CD provided approximately 94% photostabilization, and disruption of guest self-association was proposed as one contributor to the protective effect [43]. These observations illustrate that concentration can influence the relative populations of monomeric, aggregated, and complexed species and thereby alter the dominant photochemical pathway. A related effect was observed in IAD dye/β-CD nanoassemblies, where β-CD suppressed bimolecular exciton-exciton annihilation under intense excitation [12].

### 2.3. Microenvironment and Access of Reactive Species

The cyclodextrin cavity provides a physicochemical environment that differs from the surrounding aqueous phase in polarity, hydration, hydrogen-bonding capacity and steric accessibility. Inclusion can therefore alter the exposure of selected regions of a guest molecule to water, molecular oxygen and reactive oxygen species. At the same time, the cavity is not an impermeable barrier; small molecules and reactive intermediates may still reach the included guest, depending on the depth of inclusion, cavity occupancy and the dynamics of the complex.

**Figure 3:**
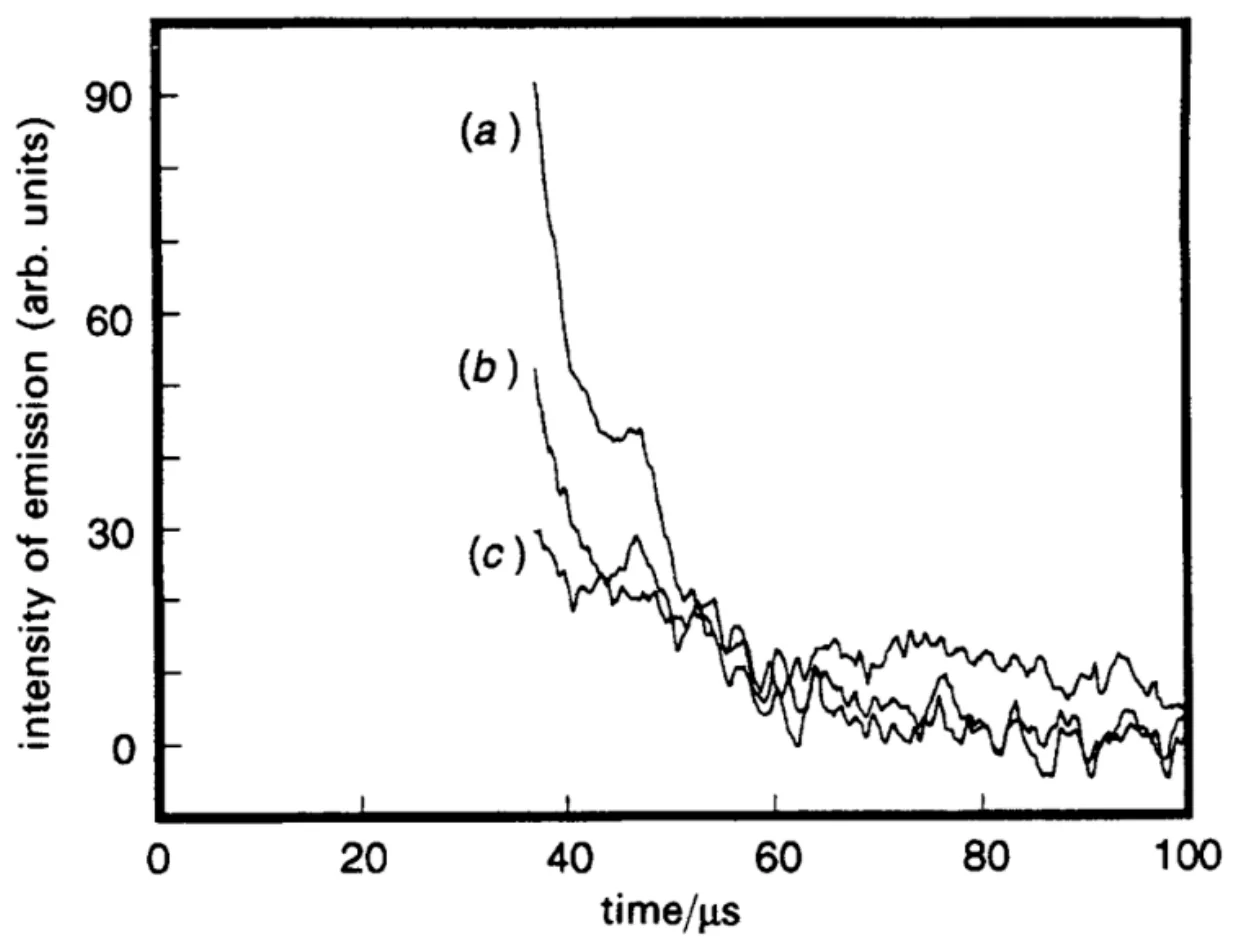
Effect of β-CD on singlet-oxygen emission in the HITC system. Time-resolved decay of singlet-oxygen (^1^O_2_) emission from HITC solutions (a) without β-CD, (b) at a β-CD:HITC molar ratio of 4.3 × 10^1^:1, and (c) at a ratio of 2.8 × 10^2^:1. Increasing β-CD concentration reduced the initial ^1^O_2_ emission intensity and prolonged the singlet-oxygen lifetime, consistent with both reduced ^1^O_2_ generation and weaker interaction of ^1^O_2_ with HITC upon complex formation. Reproduced, with permission, from Matsuzawa et al. (1994) [44].

A clear example is provided by the cyanine dye HITC complexed with β-CD. HITC photobleaching involves singlet oxygen generated by energy transfer from the excited dye to molecular oxygen. In the presence of β-CD, the photodegradation quantum yield decreased, ^1^O_2_ formation was reduced, and its subsequent reaction with HITC was also inhibited [44]. Direct monitoring of singlet oxygen through its characteristic near-infrared emission allowed two different effects to be separated: reduced formation of the reactive species and reduced accessibility of that species to the substrate. The effect of β-CD on singlet-oxygen emission and decay in the HITC system is illustrated in Figure 3.

Related effects have also been reported in pharmaceutical systems. SBE-β-CD reduced the photodegradation rate of bufexamac and strongly suppressed superoxide generation during UVA/UVB exposure [55]. In the case of chlorpromazine, both β-CD and DM-β-CD reduced UVA-induced radical formation, photobinding of chlorpromazine-derived species to bovine serum albumin and penetration into the skin, with DM-β-CD producing the stronger overall effect [56]. The consequences of complexation may extend beyond preservation of the parent compound to the formation, diffusion and interaction with biological targets of photoactivated species.

Microenvironmental effects may also influence different degradation pathways of the same molecule in opposite directions. RM-β-CD protected β-lapachone against hydrolytic degradation in darkness but accelerated its decomposition under irradiation [28]. This contrasting behavior may be rationalized by the partial inclusion geometry proposed on the basis of molecular modeling. In such an arrangement, the CD cavity could preferentially reduce solvent accessibility to the hydrolysis-susceptible pyran-ring region, while leaving the photoactive quinone moiety relatively exposed at or near the cavity opening. Consequently, the molecular regions involved in hydrolytic degradation may be more effectively shielded than those participating in light-induced reactions. Although exposure of the quinone chromophore could facilitate photochemical pathways, including potentially radical-mediated processes, the available evidence does not establish a specific radical mechanism responsible for the accelerated photolysis. This example demonstrates that selective molecular shielding can suppress one degradation pathway without preventing, and potentially even favoring, another.

The local environment created by the CD cavity can also modify the energetics of photochemical processes through polarity and specific intermolecular interactions. Tønnesen and co-workers proposed that hydrogen bonding within CD-containing curcumin systems contributed to changes in excited-state behavior [50]. A particularly instructive example was reported by Masai and co-workers, who demonstrated that the hydrophobic environment of permethylated α-CD reduced the photoreactivity of a coumarinylmethyl ester by limiting the polar stabilization of a contact ion-pair (CIP) intermediate involved in photocleavage [34]. From a physical-organic perspective, the relatively low dielectric permittivity of the CD cavity is expected to provide less favorable electrostatic stabilization of charge-separated species than a polar solvent environment. This reduced stabilization may render the ion-pair-forming pathway energetically less favorable and modify the effective activation free energy (ΔG‡) associated with photocleavage. However, neither the local dielectric permittivity nor the corresponding activation free-energy barrier was directly quantified in the cited study. Importantly, this example demonstrates that cyclodextrins do not merely provide steric shielding against external reactants or incident radiation, but can actively modulate photochemical reaction pathways by altering the relative energetics of reactive intermediates.

### 2.4. Excited-State and Radical Pathways

Beyond changing the local environment and access of external reactants, cyclodextrins can also influence the pathways available to an excited guest molecule. For many photolabile compounds, cyclodextrin complexation exerts its main effect after light absorption by altering excited-state processes. Following excitation, the guest may return to the ground state through radiative or non-radiative processes, enter triplet-state or electron-transfer pathways, generate radicals or reactive oxygen species, or undergo irreversible photochemical reactions. By modifying molecular mobility, local polarity and interactions with reaction partners, a cyclodextrin can shift the balance among these competing pathways without necessarily affecting the initial absorption of light [25,45].

This type of photochemical modulation was examined in detail by Sortino and co-workers in a diflunisal-cyclodextrin system. They combined steady-state photolysis, laser-flash photolysis and photoproduct analysis to study the effects of diflunisal complexation with a cationic amphiphilic cyclodextrin. Complexation reduced both triplet-state formation and photoionization efficiencies by approximately 50%, altered subsequent radical reactions and suppressed the major toxic photoproduct. At the same time, overall photodegradation decreased by about fourfold [45]. Cyclodextrin complexation thus affected not only the rate of parent-compound loss, but also the reactions occurring after excitation.

Individual photophysical parameters should be interpreted in relation to the full set of competing excited-state pathways. An increase in triplet lifetime, for example, may provide more time for reaction with molecular oxygen or other substrates, whereas a reduction in triplet yield may decrease the number of molecules entering this pathway. This distinction is illustrated by promazine and chlorpromazine, for which increasing complexation with HP-β-CD progressively altered triplet-state decay kinetics [57]. However, these changes were not accompanied by a matched quantitative measurement of parent-compound loss under irradiation and therefore do not, by themselves, demonstrate improved chemical photostability. Similarly, enhanced fluorescence may reflect reduced non-radiative decay without necessarily indicating greater chemical stability. Photostability is ultimately determined by the balance between relaxation and reactive processes rather than by any single excited-state parameter.

### 2.5. Why Does Cyclodextrin Complexation Not Always Confer Photoprotection?

The same factors that can improve photostability may, under different conditions, have little effect or even promote photodegradation. Inclusion changes several properties simultaneously, including guest orientation, conformational freedom, solvation, aggregation and access to reactive partners. The overall outcome depends on which of these factors governs the dominant degradation pathway before and after complexation. Formation of an inclusion complex should therefore not be regarded as evidence of photoprotection.

Relatively small changes in guest structure can reverse the effect of the same host. In a series of substituted 1,4-dihydropyridines, β-CD protected nitro-substituted derivatives but accelerated the photodegradation of several halogen- and cyano-substituted analogues [58]. Closely related curcuminoids also responded differently to HP-β-CD; some derivatives were stabilized, whereas others showed shorter photochemical half-lives after addition of the cyclodextrin [26]. Photoprotection therefore cannot be assigned as a general property of a particular cyclodextrin independently of guest structure.

Host structure can be equally important in determining the photochemical outcome. Nicardipine showed protective, neutral and adverse responses to different native and substituted cyclodextrins [27]. The methoxycinnamoyl-CD systems discussed above provide a related example, as protection depended on the match between the chromophore and the host cavity [53]. Together, these observations show that host selection should consider the geometry and local environment of the photoactive region rather than binding affinity alone.

Concentration can also change the balance between different forms of the guest in solution. Voulgari and co-workers examined the effect of β-CD on tenoxicam photodegradation and reported a concentration-dependent reversal of its influence: at low initial drug concentrations, complexation modestly retarded photodegradation, whereas at higher concentrations it accelerated the process [29]. NMR measurements provided evidence of tenoxicam self-association, suggesting that CD-induced changes in the equilibrium between monomeric, dimeric and complexed species may influence the photochemical pathways available after excitation. This behavior also complicates kinetic interpretation. If the relative populations of these species change appreciably during irradiation, the apparent degradation kinetics may deviate from a simple pseudo-first-order model, potentially producing nonlinear logarithmic concentration–time plots. However, self-association alone does not establish a change in reaction order, and the applicability of a particular kinetic model should be verified experimentally. Thus, the same guest–host pair may exhibit protective or adverse effects depending on the initial concentration and the resulting distribution of molecular species.

Solvent composition introduces another important source of variability. Pajoubpong and co-workers investigated the effect of β-CD on avobenzone photostability in ethanol-water mixtures and found a strong dependence on solvent composition. β-CD substantially improved avobenzone recovery in 10% ethanol/water, whereas in 30% ethanol the free filter was already considerably more photostable and cyclodextrin complexation provided no significant additional benefit [59]. In a separate study, Kuroda et al. showed that the photoprotection also depended on host concentration. Avobenzone showed optimal UVA1 photoprotection within an intermediate β-CD concentration range, whereas higher host concentrations produced spectral changes attributed to aggregation of AVB/β-CD-containing species [60]. Formulation architecture can also influence the outcome. Karpkird and co-workers examined cross-linked CD polymers and showed that changing the crosslinker altered the photostability of the same guest molecules, resulting in stabilization, little benefit, or increased degradation [61].

Cyclodextrin-mediated photostability is not an intrinsic consequence of inclusion. It depends on the combined influence of guest structure, inclusion geometry, host chemistry, concentration, self-association, solvent composition and excited-state processes. The key question is not simply whether a guest forms a stable complex, but whether the resulting supramolecular environment suppresses the photochemical pathway responsible for degradation or redirects excitation toward an alternative reaction.

## 3. Analytical Assessment of Cyclodextrin-Mediated Photostability

**Figure 4:**
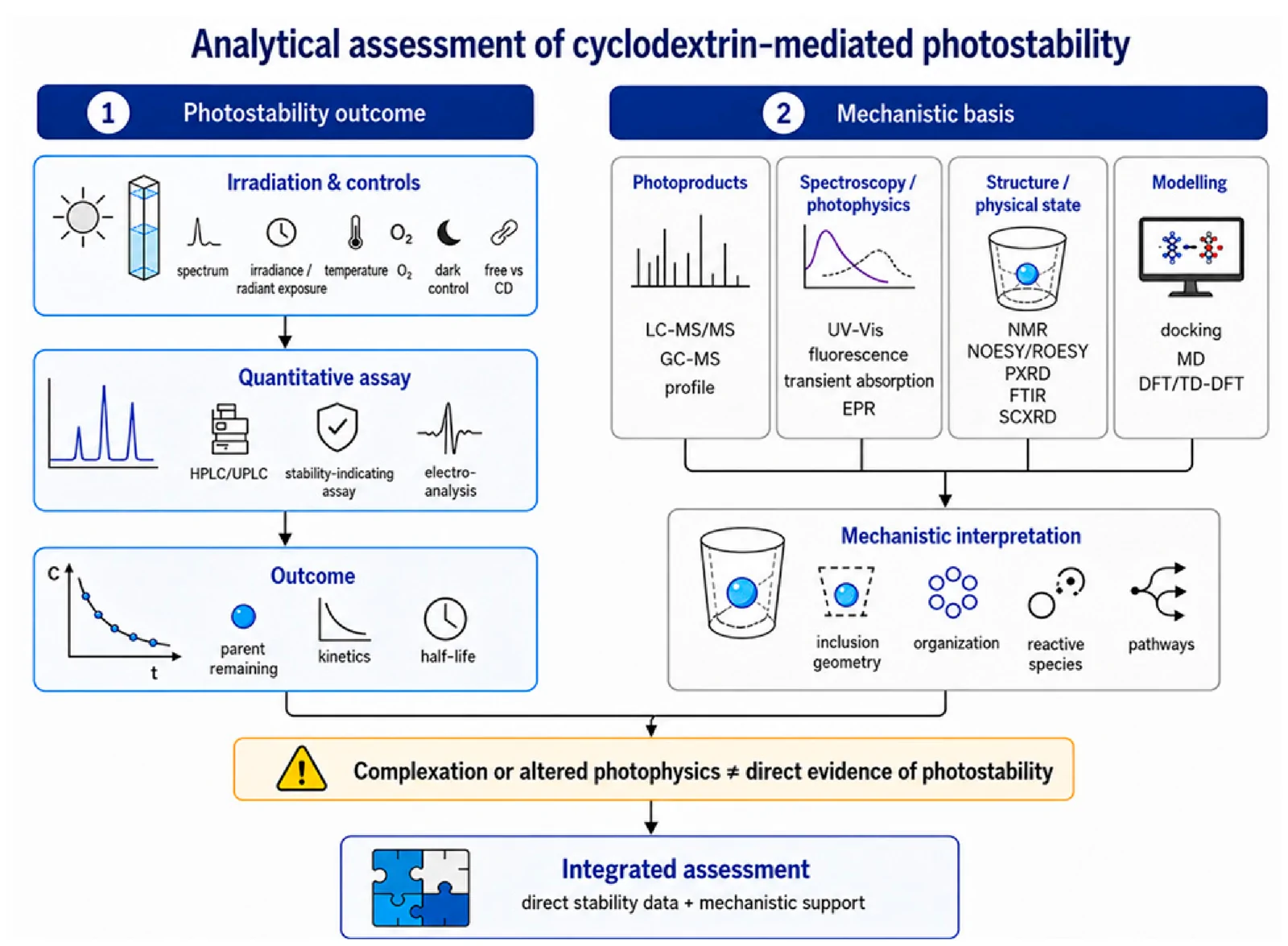
Analytical framework for assessing cyclodextrin-mediated photostability.

Reliable evaluation of cyclodextrin-mediated photostability requires more than monitoring an optical signal during irradiation. The key questions are whether complexation reduces degradation of the parent compound, whether it alters the pattern of photoproducts, and whether the observed effect can be linked to the CD-containing system under suitable control conditions. Direct quantitative measurements show whether chemical photostability has changed, whereas spectroscopic, photophysical, structural and computational methods help interpret the observed changes. The strongest evidence combines controlled irradiation with quantitative analysis of the parent compound and, where relevant, additional information on photoproducts, host–guest organization or excited-state processes [43,45,62]. This analytical framework is summarized in Figure 4.

### 3.1. Irradiation Conditions and Experimental Controls

A well-defined irradiation protocol is essential for reliable photostability assessment. Photochemical response depends on the wavelength range and intensity of the irradiation source, total radiant exposure, sample configuration, temperature and oxygen availability. Irradiation time alone is therefore insufficient for reproducibility or meaningful comparison between studies. For pharmaceutical systems, ICH Q1B provides a useful reference framework: confirmatory testing requires exposure to at least 1.2 million lux·h of visible light and 200 Wh m^−2^ of near-UV radiation using defined light sources [4,63]. Studies focused on photochemical mechanisms need not follow this protocol, but irradiation conditions should be reported in sufficient detail to define the exposure conditions.

Parallel dark controls should be regarded as a fundamental methodological requirement in photostability studies. For each irradiated formulation, an otherwise identical non-irradiated sample should be maintained under matched conditions of temperature, composition, solvent, pH and exposure duration. Temperature matching is particularly important because irradiation may cause sample heating, potentially accelerating thermal degradation independently of photochemical processes. Without such controls, losses arising from thermolysis, hydrolysis, solvolysis or excipient-induced degradation cannot be reliably distinguished from genuine light-induced decomposition. This distinction is particularly important when cyclodextrin complexation affects the stability of the guest differently in darkness and under irradiation. The β-lapachone/RM-β-CD system provides a clear example: complexation stabilized the drug in darkness but accelerated degradation under irradiation [28]. Without appropriate dark controls, these opposing effects could be misinterpreted. Similarly, in the bufexamac/SBE-β-CD system, drug loss was observed upon irradiation, whereas the corresponding dark samples remained essentially unchanged [55]. These examples demonstrate that attributing degradation specifically to light exposure requires comparison with appropriately matched non-irradiated samples.

Appropriate matched controls are essential for distinguishing photochemical effects of cyclodextrin complexation from changes caused by other experimental factors. Free and CD-containing samples should therefore be irradiated under the same conditions, with dark controls included when non-photochemical degradation may also contribute. This distinction is particularly important when cyclodextrin complexation affects the stability of the guest differently in darkness and under irradiation. The β-lapachone/RM-β-CD system discussed earlier provides a clear example: complexation stabilized the drug in darkness but accelerated degradation under irradiation [28]. Without a non-irradiated control, these opposing effects could be misinterpreted. The importance of matched irradiated and non-irradiated controls is also demonstrated by the bufexamac/SBECD system, in which drug loss was observed upon irradiation whereas the corresponding dark samples remained essentially unchanged [55].

In complex formulations, the same external light exposure does not necessarily mean that the chromophore receives the same amount of radiation. Sample configuration, layer thickness, container properties and formulation composition can influence the effective light exposure of the drug [4,63,64,65]. In solid and multicomponent dosage forms, excipients, coatings, opacity and light scattering may further reduce the amount of radiation reaching the chromophore and thereby contribute to apparent photoprotection [66,67]. Such optical or formulation-level effects should therefore be distinguished, where possible, from molecular protection arising from host–guest association. This distinction becomes particularly important in gels, particulate carriers, nanosponges, liposomal systems and solid matrices.

### 3.2. Quantifying Parent Loss and Photoproduct Formation

Direct evidence of photostabilization requires quantitative determination of the intact guest after irradiation. HPLC and UPLC are particularly useful because they can separate the parent compound from photoproducts and formulation components. Lower degradation rates, longer half-lives or greater parent-compound recovery in CD-containing samples therefore provide direct evidence of reduced chemical loss. Although separation-based methods are most widely used, electrochemical methods can also provide direct quantitative measurements for suitable analytes, as demonstrated by square-wave voltammetric monitoring of MCPA and mecoprop [68].

The way photostability is quantified also influences how the results should be interpreted. Reporting the percentage of intact compound remaining after a single irradiation period (e.g., after 2 h) provides an endpoint measure of chemical loss but does not establish the underlying degradation kinetics. Such measurements cannot reveal induction periods, biphasic degradation profiles, changes in apparent reaction order or time-dependent effects arising from photoproduct accumulation, including potential inner-filter screening of incident radiation. Consequently, single-point measurements should not be used to infer kinetic mechanisms or calculate degradation rate constants without additional supporting data. Replicate measurements at multiple irradiation time points, covering a sufficient range of parent-compound conversion, should be regarded as a minimum requirement for reliable kinetic analysis. The suitability of the proposed kinetic model should be evaluated using appropriate goodness-of-fit statistics, including R^2^ where applicable, together with inspection of residual plots, since a high correlation coefficient alone does not establish mechanistic validity. Apparent rate constants and half-lives are meaningful only within the kinetic model and irradiation conditions from which they were derived and should not be compared quantitatively across studies using different light sources, exposure conditions or sample environments [4,42]. Interpretation becomes more complex in dynamic CD systems, where the guest continuously associates with and dissociates from the cyclodextrin. The measured degradation rate may therefore reflect contributions from both free and complexed guest molecules rather than from a single photochemical species [47].

**Figure 5:**
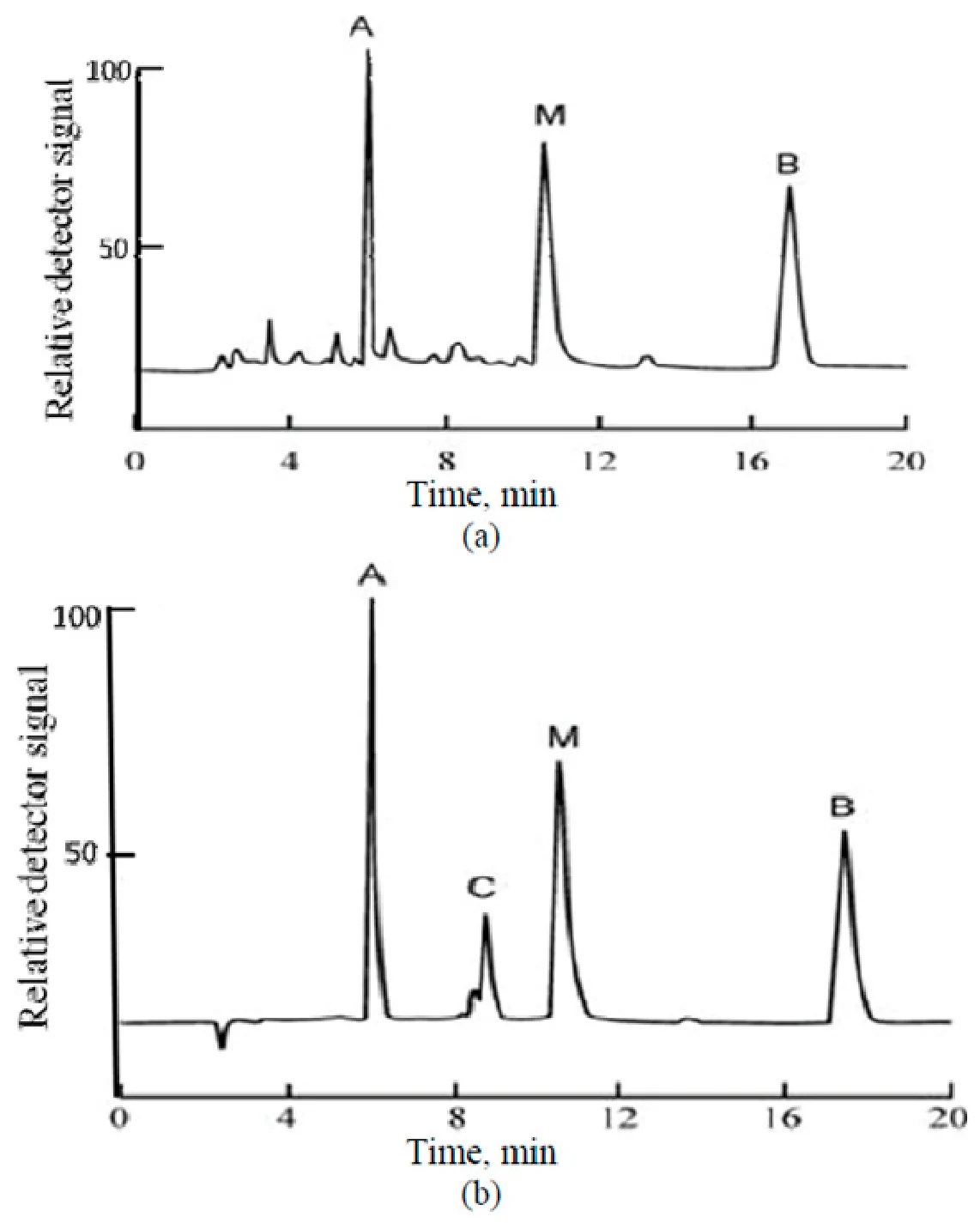
Effect of RM-β-CD on the photodegradation profile of midazolam. HPLC chromatograms obtained after 8 h of irradiation: (**a**) midazolam in the absence of RM-β-CD and (**b**) midazolam in the presence of RM-β-CD. M denotes the parent drug, whereas A, B and C denote major photodegradation products; product C was detected only in the RM-β-CD-containing system. The chromatograms demonstrate changes in the observed photoproduct profile upon complexation with RM-β-CD but do not establish a complete quantitative mass balance of the degradation products. Reproduced from Agatonovic-Kustrin et al. (2016) [69] under CC BY 4.0.

The assay must, however, remain selective in the presence of both the cyclodextrin and irradiation products. Chromatography is not inherently stability-indicating unless adequate resolution and method performance have been demonstrated. In tenoxicam/β-CD and oxolinic acid/HP-β-CD systems, discussed earlier, chromatographic methods were used to separate the parent compound from photoproducts before degradation kinetics were evaluated [29,43]. Analysis of the parent compound establishes whether degradation is slowed, whereas photoproduct analysis is needed to determine whether the degradation pathway itself has changed. The diflunisal system provides a clear example: LC-DAD-ESI-MS showed that complexation affected both parent-compound loss and photoproduct formation [45]. A contrasting example is provided by midazolam, where RM-β-CD slightly reduced photostability while simultaneously altering the photoproduct profile, including the formation of an additional intermediate (Product C) and a proposed dimeric degradation product (Figure 5) [69]. Although the authors employed HPLC and LC-MS/MS to characterize the major photoproducts and reported satisfactory analytical recovery of the parent drug, a comprehensive quantitative mass balance accounting for all degradation products was not demonstrated. This distinction is important because conventional reversed-phase HPLC may fail to detect highly retained, poorly soluble or higher-molecular-weight photoproducts, particularly when their chromatographic recovery and detector responses have not been established. Consequently, chromatographic identification of selected photoproducts does not necessarily demonstrate complete recovery of the degraded material. Nevertheless, this limitation does not invalidate quantitative determination of parent-compound loss when the parent assay is adequately validated. Comprehensive photodegradation studies should therefore combine selective quantification of the intact compound with appropriate evaluation of photoproduct recovery, using complementary analytical techniques when necessary. When volatile photoproducts are formed, complementary techniques may also be required; in ranitidine systems, for example, headspace SPME–GC–MS provided information on volatile degradation products [70].

### 3.3. Spectroscopic and Complementary Analysis of Photochemical Behavior

UV–Vis absorption and fluorescence spectroscopy are valuable for monitoring irradiation-induced changes and probing the environment of the chromophore. Complexation may alter absorption, fluorescence intensity or lifetime, aggregation-dependent spectral features, and non-radiative relaxation. Such changes can provide evidence of host–guest interactions or altered photophysical behavior, but they do not necessarily reflect the amount of intact compound [29]. The Stone and Man systems described earlier illustrate this distinction: fluorescence-based measurements can provide valuable information on photophysical behavior and resistance to photobleaching without directly measuring chemical degradation [12,54]. In fluorescence imaging, for example, sustained emissions during repeated excitation may itself represent a relevant functional measure, even when it does not directly indicate greater chemical stability.

**Figure 6:**
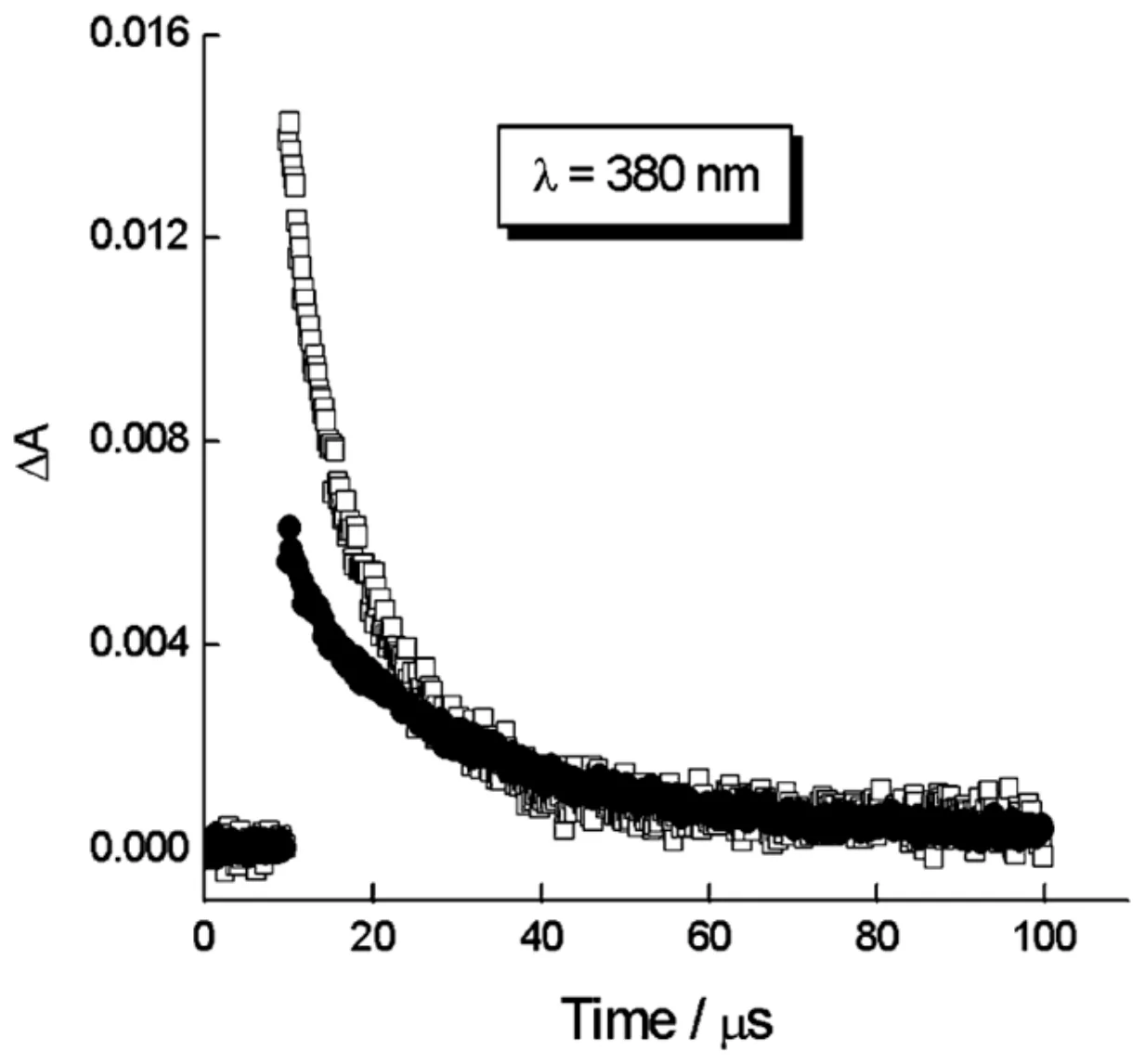
Effect of cyclodextrin association on the triplet-state kinetics of diflunisal. Transient absorption decay at 380 nm in the absence (open squares) and presence (filled circles) of SC6CDNH_2_ shows reduced triplet-state generation but a longer triplet-state lifetime after complexation. These changes were accompanied by an approximately fourfold decrease in overall photodegradation and suppression of the major toxic photoproduct. Reproduced, with permission, from Sortino et al. (2003) [45].

Time-resolved techniques provide complementary information on short-lived excited states and reactive intermediates that precede chemical degradation. Laser-flash photolysis and transient absorption can detect triplet states, radical ions and other intermediates and reveal how their formation and decay are affected by cyclodextrin complexation. The diflunisal system described earlier illustrates the value of combining time-resolved measurements with direct analysis of chemical degradation (Figure 6) [45]. By contrast, the promazine and chlorpromazine study showed changes in triplet-state behavior without a matched quantitative measurement of long-term parent-compound loss [57]. These examples show that time-resolved measurements provide important information on excited-state processes, but changes in these parameters alone do not establish photoprotection.

Reactive-species measurements provide additional information on how cyclodextrins affect ROS-related photochemical pathways. Singlet-oxygen phosphorescence, ESR and chemical ROS assays can be used to monitor reactive-species formation and, in some cases, their subsequent reactions, as illustrated by the HITC, chlorpromazine and bufexamac systems described earlier [44,55,56]. The interpretation, however, depends on the intended application. For photosensitizers, reduced photobleaching is beneficial only if the required ROS-generating activity is retained, as shown for CD-insulated porphyrin rotaxanes [33]. These measurements should therefore be interpreted in relation to the intended photochemical function and, when improved chemical photostability is claimed, together with direct quantitative analysis of the parent compound.

### 3.4. Host–Guest Organization and Computational Support

Structural and computational methods provide information that complements direct photostability measurements by showing how the guest interacts with the cyclodextrin and how its physical environment changes upon complexation. In solution, ^1^H NMR together with NOESY or ROESY can provide through-space information on host–guest proximity and constrain possible inclusion geometries. In the doxycycline/β-CD system, ROESY data were combined with docking and molecular dynamics to compare the proposed orientations [62]. The methoxycinnamoyl-CD systems introduced earlier provide another example of the structural information available from ROESY. In 4-methoxycinnamoyl-α-CD, cross-peaks between protons of the cinnamoyl chromophore and the internal H3 and H5 protons of α-CD supported self-inclusion of the photoactive moiety within the cavity [53]. In the same study, improved photostability was observed for appropriately size-matched CD-chromophore combinations, linking the ROESY-supported spatial arrangement to the photochemical outcome. Because NOESY and ROESY contacts indicate spatial proximity rather than a unique rigid geometry, they are most informative when supported by complementary structural, thermodynamic or computational evidence.

**Figure 7:**
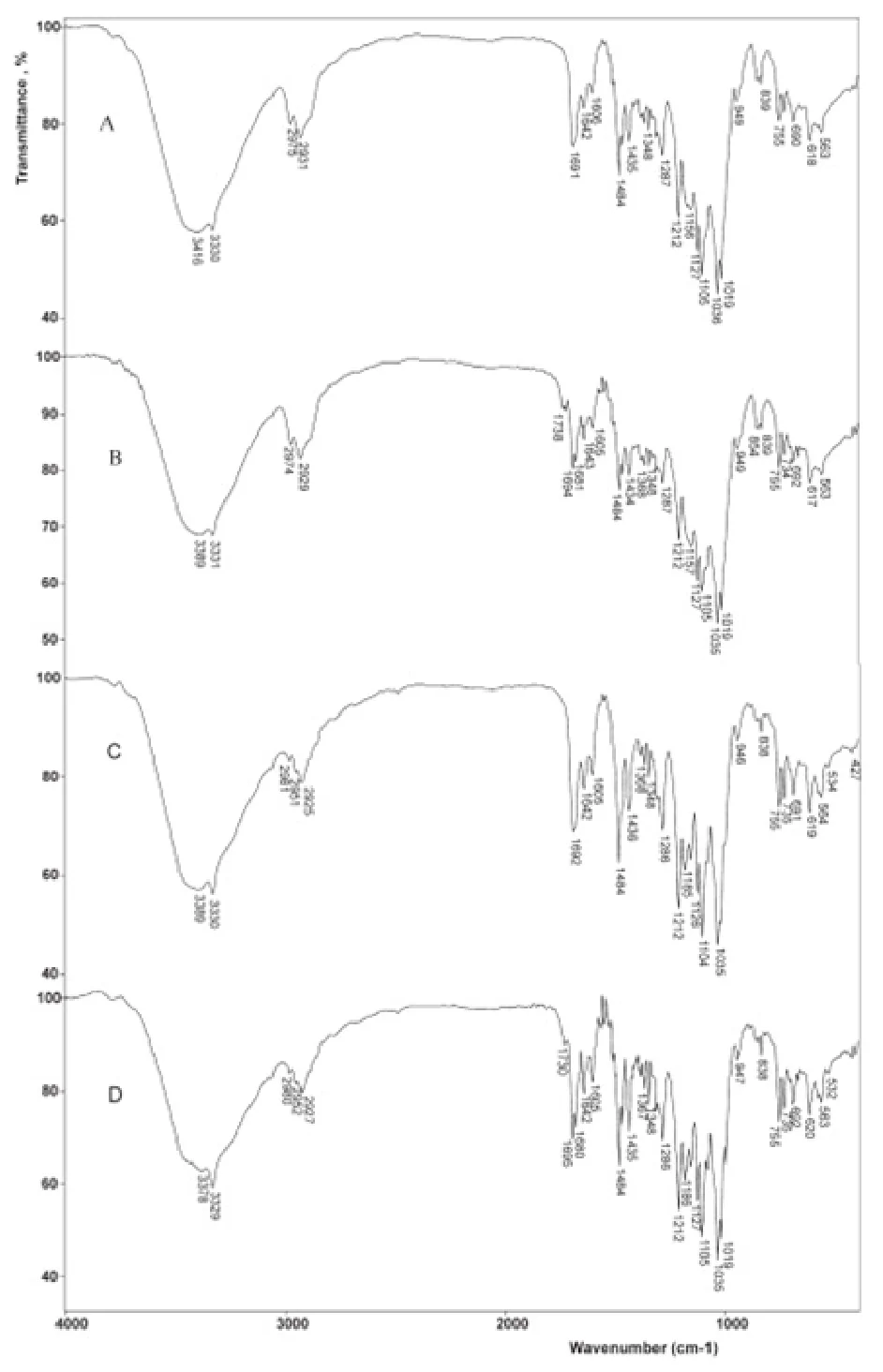
FTIR monitoring of the solid-state photostability of amlodipine/CD inclusion complexes. FTIR spectra of the amlodipine/β-CD complex before (**A**) and after (**B**) eight days of visible-light irradiation, and of the amlodipine/HP-β-CD complex before (**C**) and after (**D**) irradiation. The limited FTIR spectral changes after irradiation suggest reduced photoinduced structural alterations in the CD-containing solids; however, these observations do not quantitatively establish parent-drug retention or distinguish the effects of molecular inclusion from differences in solid-state organization. Reproduced, with permission, from Kapor et al. (2010) [72].

In solid formulations, analytical interpretation is more complex because crystallinity, hydration, molecular dispersion and amorphization may themselves affect photoreactivity. PXRD, DSC, TGA and FTIR are therefore useful for defining physical-state changes, but disappearance of a melting endotherm or diffraction peaks is not specific evidence of cavity inclusion. In methotrexate/CD formulations, DSC and PXRD revealed substantial loss of crystallinity and amorphization, while FTIR and solution NMR provided complementary evidence of host–guest interaction and HPLC independently quantified photodegradation [23]. Solid-state techniques can also be applied directly to irradiated samples to follow light-induced changes. In indomethacin/β-CD systems, thermal and vibrational measurements indicated smaller irradiation-induced changes in inclusion complexes than in corresponding physical mixtures, with the response depending on CD proportion [71]. In solid amlodipine/CD systems, FTIR spectra of the β-CD and HP-β-CD complexes remained largely unchanged after eight days of light exposure (Figure 7) [72]. In the same study, free amlodipine showed degradation-related spectral changes consistent with dehydrogenation and aromatization of the 1,4-dihydropyridine ring. These examples also highlight an important limitation of solid-state photostability comparisons. Amorphous drug phases generally possess higher Gibbs free energy than their crystalline counterparts and may exhibit increased molecular mobility, depending on temperature, moisture content and the glass-transition characteristics of the material. Furthermore, differences in crystallinity, particle size and powder packing can modify diffuse optical reflectance, light scattering and the effective irradiation dose reaching the drug. Consequently, comparing an amorphous or structurally disordered CD-containing formulation directly with an unformulated crystalline active pharmaceutical ingredient may conflate genuine host–guest inclusion effects with changes arising from amorphization and other solid-state properties. A more rigorous experimental design should therefore include a CD-free amorphous drug control with a comparable physical state, prepared and irradiated under matched conditions, whenever feasible. Without such a control, the contribution of molecular inclusion to the observed photoprotection cannot be quantitatively separated from that of solid-state disorder and formulation-dependent optical effects.

Solid-state NMR can provide more detailed molecular information when diffraction methods are limited by amorphous or disordered samples. In ATRA/HP-β-CD pellets, solid-state ^13^C NMR revealed clear changes in resonances assigned to the retinoid side chain, supporting inclusion of the region extending from the carboxyl terminus toward the C9=C10 segment. This interpretation was supported by DSC, PXRD and FTIR data and was consistent with independently measured photostability: after 10 days of illumination, 84.08 ± 1.98% of ATRA remained in the HP-β-CD formulation compared with 77.02 ± 0.21% of free ATRA [73]. This combination of structural and quantitative stability data is especially useful because it links evidence of inclusion with the observed behavior under irradiation.

When suitable crystals are available, single-crystal X-ray diffraction can provide direct information on crystal packing and host–guest organization. Its usefulness, however, depends on how well the guest can be resolved. In the β-CD/avobenzone system reported by d’Agostino et al., single-crystal data confirmed the unit cell, space group and channel-like β-CD packing, but severe static and dynamic disorder of avobenzone prevented an unambiguous determination of its inclusion geometry [10]. This example illustrates that even high-resolution structural methods may not fully define guest orientation in disordered systems.

**Figure 8:**
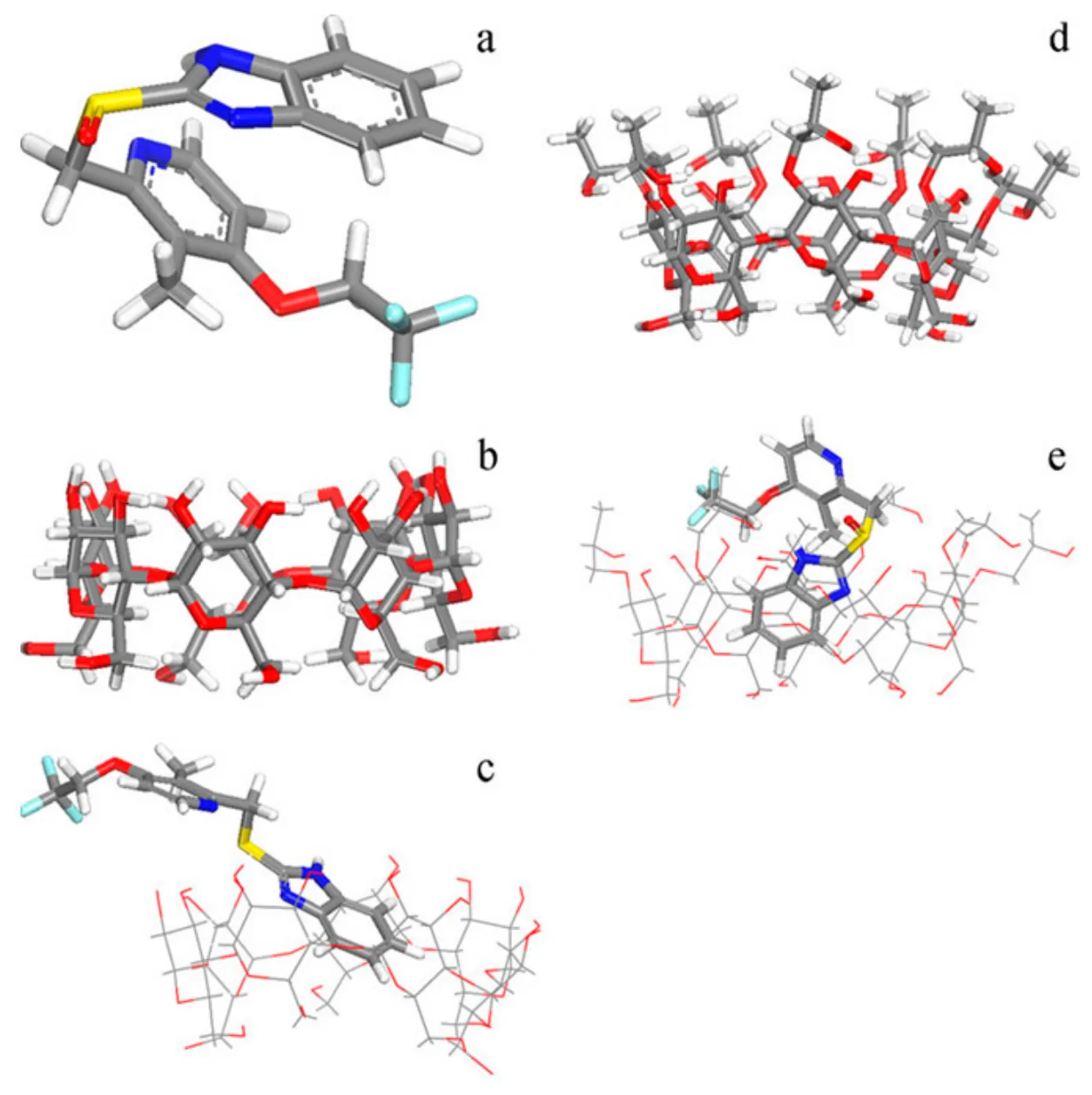
Molecular modeling of lansoprazole inclusion in β-CD and HPCD as a structural explanation for cyclodextrin-dependent photostability. (**a**) Lansoprazole (LSP), (**b**) β-CD, (**c**) modeled LSP/β-CD inclusion complex, (**d**) HPCD, and (**e**) modeled LSP/HPCD inclusion complex. In the β-CD complex, the photolabile sulfinyl moiety remains close to the cavity opening, whereas in the HPCD complex it is more deeply shielded by the hydroxypropyl-substituted host. This difference in inclusion depth is consistent with the experimentally observed higher photostability of LSP/HPCD compared with LSP/β-CD. Reproduced, with permission, from Lu et al. (2012) [75].

Computational methods can complement experimental structural data by suggesting inclusion geometries and examining guest orientation, interaction energies, solvent exposure, or molecular mobility. In methoxy cinnamic acid/CD systems, optimized structures showed differences in cavity fit, guest orientation, and hydrogen bonding [74]. In lansoprazole/CD systems, modeling helped interpret experimentally observed differences between β-CD and HPCD complexes (Figure 8) [75]. These examples show the value of modeling for interpreting experimental observations, but computational results alone do not demonstrate photostability. They are most informative when supported by structural data and direct measurements of degradation under irradiation.

These analytical approaches provide different types of evidence. Direct quantitative measurement of the parent compound under matched irradiation conditions gives the clearest evidence of chemical photostability, whereas spectroscopic, photophysical, structural, functional, and computational methods provide complementary information on the observed changes and their possible causes. In complex formulations, appropriate controls are also needed to distinguish CD-specific effects from protection provided by the formulation itself. The strength of a conclusion therefore depends not on whether the observed effect is positive or negative, but on how directly experimental evidence supports it.

## 4. Cyclodextrins as Modulators of Photostability

Experimental studies of cyclodextrin-mediated photostability span chemically and functionally diverse guests, including pharmaceuticals, natural bioactives, UV filters, molecular probes, photosensitizers and agrochemicals [1,36,42]. Across these application areas, CD-containing systems can substantially reduce light-induced degradation, but negligible, condition-dependent and adverse effects are also well documented [1,37,42]. The following sections examine the range of outcomes reported across these application areas and highlight representative examples of both effective and limited photoprotection. Studies reporting photostabilization are summarized in Table 1, Table 2, Table 3, Table 4 and Table 5, whereas representative examples of absent, adverse and strongly condition-dependent effects are highlighted in Table 6 to provide a more balanced presentation of the available evidence. A comprehensive compilation of such findings is provided in Supplementary Table S2, while studies based on indirect photostability assessments or those in which the specific contribution of cyclodextrin could not be clearly distinguished are summarized in Supplementary Table S3. To connect these application-specific findings with the mechanistic framework established in Section 2, each subsection concludes with a synthesis relating representative outcomes from Table 1, Table 2, Table 3, Table 4 and Table 5 to the proposed molecular processes. Where the available evidence does not identify a unique dominant pathway, mechanistic assignments are treated as tentative rather than definitive.

### 4.1. Pharmaceuticals

Pharmaceuticals constitute one of the largest and most diverse groups of photosensitive guests investigated in CD-containing systems. The literature encompasses anti-inflammatory, antibacterial, cardiovascular, anticancer, dermatological and psychotropic drugs in solution, solid products and formulated dosage forms [1,2,35,36]. Reported outcomes range from marked stabilization to weak, absent or adverse effects, even among related pharmaceutical compounds [37,38,42]. The pharmaceutical evidence is summarized in Table 1.

**Table 1:** Photostability of pharmaceutical compounds in cyclodextrin-containing systems.

Guest/Application	Cyclodextrin System	Irradiation/Assessment	Photostability Outcome	Supporting Evidence	Interpretation	Ref.
Analgesic and anti-inflammatory agents						
Bufexamac—topical NSAID with phototoxic potential	SBE-β-CD; 1:1, K = 620 M^−1^; aqueous inclusion complex	Suntest CPS^+^, 750 W m^−2^, 25 °C, 120 min; UPLC/ESI-MS + ROS assays	28.2% complexed vs. 18.3% free remained; k ↓ ~30%; superoxide generation reduced	UV–Vis/Scott analysis; ^1^O_2_/superoxide assays; DNA-binding/photocleavage studies	Protection extends beyond parent retention to suppression of ROS-mediated photochemistry	[55]
Diclofenac—topical NSAID	β-CD selected (α-CD, methyl-β-CD, γ-CD screened); ≈1:1, K_b_ = 1361 M^−1^; β-CD gel	Suntest CPS^+^ xenon, ICH/ID65, 300–800 nm, 450 W m^−2^; UV–Vis/MCR-ALS	t_0.1_: 25.01 min with β-CD vs. 3.90 min control gel and 4.38 min Voltaren Emulgel^®^ 1% (~6-fold vs. commercial 1% gel)	Phase solubility/binding; validated extraction; chemometric photoproduct analysis; UV-filter comparators	β-CD provided strong formulation-level protection and outperformed the tested UV absorbers	[76]
Diflunisal—phototoxic NSAID	SC6CDNH_2_, cationic amphiphilic β-CD derivative; association shown spectroscopically; self-assembled nanoaggregates	UVA 310–390 nm; 266 nm laser-flash photolysis; LC-DAD-ESI-MS	~4-fold lower photodegradation; triplet/photoionization efficiencies ↓ ~50%; major toxic photoproduct completely suppressed	ICD, fluorescence, transient spectroscopy and LC-MS photoproduct analysis	A strong mechanistic example in which CD alters excited-state/radical pathways, not only parent loss	[45]
Indomethacin—NSAID	β-CD; IND:β-CD 1:1, 1:0.5 and 1:0.1; freeze-dried complexes and physical mixtures	Suntest CPS^+^ xenon/ICH, 500 W m^−2^, 45 °C, 12–48 h; UV-Vis, FTIR/ATR-FTIR, DSC	1:1 and 1:0.5 inclusion products showed greater spectral/thermal stability than the low-CD 1:0.1 system and physical mixtures	Fluorescence, Job/Benesi–Hildebrand analysis, DSC and matched dark controls	CD-content-dependent solid-state stabilization was observed, although the endpoint was mainly spectroscopic/thermal	[71]
Naproxen—NSAID	HP-β-CD; 1:1 preparation ratio; coprecipitated solid complex	Natural daylight, 15–30 d; FTIR and UV-Vis	FTIR changes consistent with photodegradation appeared in free naproxen after 15 days of daylight exposure, whereas the HP-β-CD complex remained spectrally essentially unchanged through 30 days	PXRD, ^1^H NMR, FTIR; physical-mixture comparison	Solid inclusion delayed daylight degradation; structural and physical-mixture controls support complex formation	[77]
Piroxicam—oxicam NSAID	HP-β-CD; 1:1; coprecipitated complex with physical-mixture comparator	Daylight, up to 30 days; FTIR	Free drug showed degradation after 5 days; complex remained spectrally stable up to 30 days	PXRD, ^1^H NMR and FTIR	Suggests solid-state photoprotection, but chemical specificity is limited by an FTIR-only photostability endpoint	[78]
Sufentanil-potent opioid analgesic	HP-β-CD; 1:1, K_a_ = 515.2 ± 1.2 M^−1^; freeze-dried inclusion complex	1.8 kW xenon lamp, 30 cm, 30 °C, 0–6 h; HPLC	Free drug decreased and formed additional degradant peaks; only minor chromatographic changes in the complex	^1^H/DOSY NMR, PXRD, DSC, FTIR; release and biological safety/activity studies	Chromatographic evidence indicates reduced parent loss and degradant formation after inclusion	[79]
Antibacterial and antifungal agents						
Doxycycline hyclate—photosensitive tetracycline antibiotic	β-CD; 1:1; freeze-dried inclusion complex	UV exposure, 20 cm, 30 °C, 3–6 h; HPLC-UV; wavelength/irradiance not reported	After 6 h: 96.8 ± 0.9% complexed vs. 68 ± 1% free remained; antibacterial activity better preserved	^1^H NMR/ROESY, FTIR, DSC/TGA, docking, MD/MM-PBSA, microbiological assay	Direct HPLC and functional retention support protection; incomplete irradiation characterization is the main limitation	[62]
Doxycycline hyclate—tetracycline antibiotic	α-CD, γ-CD, HP-β-CD, RM-β-CD; drug:CD 1:2–1:20; freeze-dried complexes	Short-wave UV, long-wave UV and visible light for 2 weeks; UV-Vis at 274 nm	All CDs improved retention; γ-CD 1:20: 69.93 ± 2.4% vs. 14.97 ± 1.6% free; γ-CD > RM-β-CD > HP-β-CD > α-CD	Drug-content assay, SEM, PXRD and membrane-permeation studies	Host identity and CD excess strongly affected the magnitude of protection	[80]
Natamycin—polyene antifungal/food preservative	Me-β-CD selected (β-CD, HP-β-CD, maltosyl-β-CD screened); 1:1 modeled; aqueous system	365 nm, 50 W, 30 cm; UV-Vis retention and first-order kinetics	T_1/2_ increased from 59.2 to 157.5 min; antifungal activity retained after UV	Solubility/pH stability, Aspergillus niger assay, 30 ns MD/PMF	Me-β-CD combined photoprotection with preservation of antifungal function	[81]
Oxolinic acid—quinolone antibacterial	HP-β-CD; 1:1 aqueous inclusion complex	Suntest CPS xenon, 750 W m^−2^, ICH; HPLC-PDA kinetics; dark control	Photodegradation reduced by ~94%	UV–Vis, ^1^H/2D ROESY NMR, Job/Scott analysis and binding constant	A strong direct study; disruption of guest self-association was proposed as part of the protective mechanism	[43]
Sulfanilamide—bacteriostatic sulfonamide	β-CD and HP-β-CD; 1:1, K ≈ 320–330 M^−1^; coprecipitated solids	UVB, λmax 300 nm, 12 W m^−2^, 0–300 s; UV-Vis at 258 nm	After 300 s: 41.88% free, 46.44% β-CD and 57.67% HP-β-CD remained	Phase solubility, FTIR, ^1^H NMR, PXRD and DSC	HP-β-CD gave greater protection than native β-CD despite similar binding constants	[82]
Anticancer and immunosuppressive agents						
Doxorubicin—anthracycline anticancer drug	HP-β-CD; 1:1 by fluorescence titration; aqueous complex	15 W fluorescent lamp, 20 cm, up to 150 min; UV-Vis kinetics	k = 0.0329 h^−1^ complexed vs. 0.111 h^−1^ free (~3-fold reduction)	Fluorescence titration, FTIR, DSC, PXRD and PM3 modeling	Complexation substantially slowed photodegradation in solution	[83]
6-Mercaptopurine monohydrate—antimetabolite	β-CD; 1:1 by UV-Vis/Job; solid complex from 10% ethanol/water	Visible sunlight, 120 min; UV-Vis at 324 nm	Degradation: 90.60% free vs. 75.94% complexed	FTIR, ^1^H NMR, PXRD, DSC/TGA, SEM, DFT and bioassays	Only modest apparent protection; non-standardized sunlight and UV-Vis-only quantification weaken the evidence	[84]
Methotrexate—antimetabolite/anticancer drug	β-CD; 1:7 preparation ratio; freeze-dried complex, later incorporated into SMEDDS	Daylight 3 months and forced UV/visible light 10 days; UV–Vis	Complex remained essentially unchanged; free MTX lost ~40% with precipitation/color change	Phase solubility, DSC, FTIR; SMEDDS characterization and pharmacokinetics	Strong apparent stabilization despite a spectroscopic endpoint; SMEDDS was introduced only subsequently	[85]
Methotrexate—antimetabolite/anticancer drug	β-CD, HP-β-CD, M-β-CD, DM-β-CD; 1:1; spray-dried amorphous complexes	25 °C/60% RH, 20 d, 1.2 million lux·h; HPLC + color assessment	Free MTX loss 36.8 ± 1.7% vs. ~6.7 ± 1.9% for CD complexes	Phase solubility, DSC/PXRD/FTIR, ^1^H/NOESY NMR, SEM; dissolution and permeability/PK	HPLC confirms improved photostability of CD-containing solid formulations; the contributions of molecular inclusion, amorphization and formulation-dependent optical effects were not independently resolved.	[23]
Antihistamines and decongestants						
Loratadine—second-generation H_1_ antihistamine	α-CD, β-CD, γ-CD; preparation ratios 1:1–1:3; coevaporated products	ICH Q1B option 2, 12 h, 25 °C; HPLC-DAD	At 1:1: 99.15% γ-CD, 96.36% α-CD, 93.63% β-CD recovery; higher CD ratios generally less protective	DSC, TG/DTG, factorial design and ANOVA/response-surface analysis	More CD was not necessarily better; stoichiometric/formulation ratio strongly influenced protection	[86]
Naphazoline, xylometazoline, antazoline—nasal/ophthalmic imidazolines	β-CD; 1:1 association from previous inclusion studies; aqueous systems	150 W mercury UV lamp; UV-Vis kinetics and HPLC	HPLC recovery: NP 81.1 → 98.3%, XM 40.5 → 57.7%, AZ 11.9 → 20.0%	UV-Vis, HPLC and molecular-mechanics modeling	Magnitude of protection varied markedly among closely related guests	[52]
Antiparasitic agents						
Benznidazole—Chagas-disease antiparasitic	RM-β-CD (MS 0.5); 1:1 by Job/^1^H NMR; aqueous complex	254 nm, 22 W, 15 cm, 28 °C, 0–6 h; HPLC	Reported k_obs_: 9.17 × 10^−1^ (free) vs. 6.49 × 10^−1^ min^−1^ (complexed); the reported units are inconsistent with the experimental time scale and remain unverified.	Phase solubility, UV-Vis, HPLC, ^1^H NMR/Job; trypanocidal and MTT assays	Apparent kinetic protection was observed, but the reported rate units are inconsistent with hour-scale sampling	[87]
Hydroxymethylnitrofurazone (NFOH)—anti-Chagas prodrug candidate	HP-β-CD (DS ≈ 4.2); 1:1, K ≈ 6–8 M^−1^; aqueous complex	1.8 kW xenon, >~310 nm, 30 °C, 0–6 h; HPLC	Reported k_obs_ decreased from 5.19 × 10^−1^ to 3.83 × 10^−1^ min^−1^; the reported units appear inconsistent with the 0–6 h sampling protocol.	RP-HPLC complexation, phase solubility, Job plot, ^1^H NMR/T_1_ and MTT	The abstract describes a destabilizing effect, whereas the HPLC-based results report a lower degradation rate constant after complexation and conclude that HP-β-CD improves photostability.	[88]
Cardiovascular agents						
Amlodipine besylate—calcium-channel blocker	β-CD, HP-β-CD; 1:1 coprecipitated complexes; physical mixtures	Daylight, 0–8 days; FTIR in KBr pellets	Free drug showed DHP → pyridine spectral changes after 8 days; none detected in CD complexes	^1^H NMR, PXRD, DSC and FTIR	Solid-state complexes suppressed detectable photooxidative spectral conversion; chemical quantification was not used	[72]
Eleven 1,4-dihydropyridines—antihypertensives	Methyl-β-CD; 1:1 inclusion complexes	Suntest CPS^+^ xenon, ICH/D65, 300–800 nm, 350 W m^−2^, 25 °C; UV-Vis kinetics	Rates decreased for amlodipine, felodipine, nisoldipine and nitrendipine; little change for lercanidipine/manidipine	Job method and inclusion-efficiency analysis; liposome comparator	A class-level example showing that protection tracks guest fit/inclusion efficiency rather than drug class alone	[89]
Barnidipine—photosensitive DHP calcium-channel blocker	α-, β-, γ-CD, methyl-β-CD, HP-β-CD screened; ≈1:1; CD, liposome and drug-in-CD-in-liposome systems	Suntest CPS^+^ xenon, ICH/D65, 300–800 nm, 250 W m^−2^; derivative UV	After 225 kJ m^−2^: 29.81% ethanol, 42.90% liposomes, 72.03% methyl-β-CD, 90.78% β-CD-in-liposome	Phase solubility/K, inclusion efficiency, microscopy and gel filtration	Hierarchical carrier design provided greater protection than either CD or liposomes alone	[64]
Carvedilol—β-blocker/heart-failure drug	β-CD and HP-β-CD; 1:2; coprecipitated solids	UVB 300 nm, 14.5 W m^−2^, up to 3600 s; UV-Vis	After 1500 s: ~12% loss free, 6.56% β-CD, 9.17% HP-β-CD	Phase solubility, FTIR, ^1^H NMR and PXRD	Native β-CD outperformed HP-β-CD despite the latter’s higher reported association parameter	[90]
Cilnidipine—L-/N-type calcium-channel blocker	HP-β-CD; 1:1, K_1:1_ = 50,116 M^−1^; freeze-dried complex	4500 lx, up to 24 h; HPLC; temperature inconsistently reported (25 vs. 40 °C)	After 24 h: >25% loss free vs. ~3–4% loss complexed; initial lag phase	Phase solubility, UV–Vis, HPLC, DSC, PXRD, FTIR, ^1^H NMR	HPLC supports improved photostability, but inconsistent temperature reporting (25 vs. 40 °C) limits reproducibility and prevents reliable separation of complexation-dependent and thermal effects.	[91]
Isradipine—DHP calcium-channel blocker	Methyl-β-CD (DS 1.8); 1:1; aqueous Britton-Robinson-buffer complex	ICH/HBO-200, up to 200 min; UV–Vis, RP-HPLC and HPTLC	k 12.02 → 6.52 × 10^−3^ min ^−1^; t_1/2_ 57.65 → 106.2 min; photoproduct profile unchanged	Phase solubility and validated UV–Vis/HPLC/HPTLC methods	CD mainly slowed the same photochemical pathway rather than changing product identity	[92]
Manidipine—DHP antihypertensive	γ-CD; 1:1, K = 3.39 × 10^4^ M^−1^; aqueous complex	HBO-50, 300–400 nm (λ_max_ 365 nm), up to 180 min; HPLC-PDA/ESI-MS	k 8.94 → 6.92 × 10^−3^ min^−1^; t_1/2_ 77.6 → 100.1 min; photoproduct profile unchanged	Benesi-Hildebrand analysis, actinometry and HPLC-ESI-MS photoproduct identification	Moderate protection occurred without altering the principal photoproduct pathway	[93]
Nifedipine—calcium-channel blocker	β-CD, HP-β-CD, DM-β-CD; 1:1 coprecipitated complexes	Fluorescent light or sunlight; HPLC	Degradation rates 8.5–33-fold lower under fluorescent light and 4.4–12.3-fold lower under sunlight; DM-β-CD > HP-β-CD > β-CD	DSC, PXRD, FTIR and dissolution	Host substitution and irradiation source both strongly modulated protection	[49]
Nifedipine—calcium-channel blocker	β-CD; 1:1; coprecipitated solid complex, aqueous complex also tested	Daylight 15 days and UVA 350 nm, 18 W m^−2^; FTIR + HPLC-DAD	Free solid degraded ~4–5× faster; β-CD also slowed UV photoproduct formation	PXRD, ^1^H NMR, FTIR and DSC	Protection was demonstrated in both parent-loss and photoproduct formation, with strong solid-state benefit	[94]
Propranolol hydrochloride—non-selective β-blocker	β-CD in ~107-fold molar excess; aqueous system	80 W mercury lamp, 7 cm, up to 30 min; UV-Vis and fluorescence kinetics	Degradation-rate constant reduced by ~54% by both absorbance and fluorescence	UV-Vis and fluorescence kinetic analysis	Consistent optical kinetics suggest protection, but the very large CD excess and lack of chromatographic assay limit mechanistic attribution	[95]
Propranolol hydrochloride—non-selective β-blocker	HP-β-CD (MS 0.6); 1:1/1:2 species; physical-mixture and co-ground systems	Xenon-arc UVA/UVB, 15–300 min; UV-Vis at 289.6 nm	1:2 co-ground system: degradation rate ↓ 73.1% after 1 h and 55.3% over 5 h; greater HP-β-CD content improved protection	Job plot, DSC, PXRD and 6-month storage stability	Preparation method and host ratio influenced the protective effect	[96]
Central nervous system and neurological agents						
Chlorpromazine—phenothiazine antipsychotic/photosensitizer	α-CD, β-CD, γ-CD; CPZ:CD ≈ 1:2 aqueous systems	305 and 365 nm, 90 min; GC photoproducts + guinea-pig skin response	Skin irritation decreased: β-CD > γ-CD ≫ α-CD; β-/γ-CD shifted photolysis toward less toxic promazine	GC, stability constants, Draize scoring and histology	CDs altered photoproduct distribution and biological phototoxicity, not merely parent-drug stability	[97]
Chlorpromazine phenothiazine antipsychotic/photosensitizer	β-CD and DM-β-CD; application ratios 1:1–1:3; topical/in vitro systems	UVA 320–400 nm, 10.2 J cm^−2^; skin challenge, ESR and CPZ-BSA photobinding	Both CDs reduced photoallergy, skin penetration, radicals and BSA photobinding; DM-β-CD strongest and abolished response at 1:3	GC-FID, ESR/EPR, histology; K ≈ 8300–8800 M^−1^	A biologically important example linking CD association to reduced radical chemistry and photoallergy	[56]
DY-9760e—experimental cytoprotective stroke agent	SBE-β-CD (DS 6.2); predominantly 1:1; aqueous injectable system	Fluorescent light, 2500 lx, 20 days, 25 °C; clear/amber ampoules; HPLC	SBE-β-CD reduced drug loss and color formation in clear ampoules; amber glass minimized degradation with or without CD	Circular dichroism, phase solubility, ^1^H/^13^C NMR and HPLC	Packaging can dominate the overall effect; CD protection is most relevant when the product remains light-exposed	[98]
Ergotamine tartrate—antimigraine alkaloid	TDI-crosslinked HP-β-CD nanosponges (NSP3; with caffeine)	ICH Q1B, ≥1.2 million lux·h; UV-Vis drug content; dark controls	Drug loss decreased from ~14% free to ~2.5% in NSP3	FTIR, DSC, ^1^H NMR, SEM, DLS/PDI/ζ-potential and release	Advanced crosslinked architecture produced strong formulation-level photoprotection	[99]
Mephenesin—centrally acting muscle relaxant	β-CD; 1:1, K_a_ = 2146.5 M^−1^; coprecipitated solid complex	Visible sunlight; UV-Vis at 270 nm; dose/duration not reported	Degradation 21.78% free vs. 2.92% complexed	Job plot,^1^H NMR, FTIR, PXRD, DSC/TGA, docking	Large apparent protection, but poorly characterized sunlight exposure limits quantitative comparability	[100]
Serotonin hydrochloride -neurotransmitter/CNS-active model	2-HP-β-CD; 1:1; aqueous complex; deoxygenated/N_2_ conditions	UVC 254 nm, 2 × 8 W, up to 16 min; UV-Vis kinetics	k 24.10 → 8.81 × 10^−3^ min^−1^; t_1/2_ 28.5 → 78.3 min	Phase solubility, ^1^H NMR, ICD, PXRD, thermal analysis, docking and 50 ns MD	Protection was demonstrated under oxygen-depleted conditions, emphasizing the role of the irradiation environment	[101]
Gastrointestinal agents						
Lansoprazole—proton-pump inhibitor	β-CD and HP-β-CD (MS 0.9); 1:1; fluid-bed-coated inclusion-complex pellets	4500 ± 500 lx, 5 d; residual drug by HPLC-UV	Residual drug: HP-β-CD > β-CD > free lansoprazole	DSC, PXRD, FTIR, heat/humidity stability and molecular modeling	Deeper inclusion of the photolabile sulfinyl region was associated with stronger protection	[75]
Ranitidine hydrochloride—H_2_-receptor antagonist	β-CD; 1:1 and 1:2; freeze-dried complexes and physical mixtures	Suntest CPS^+^ xenon, solar ID65/ICH, 500 W m^−2^, 48 h; HS-SPME-GC-MS	Complexes and physical mixtures reduced volatile photoproduct emission; physical mixtures showed the lowest emission/color/odor change	^1^H/^2^D NMR, FTIR, DSC/TGA, microscopy and SEM	The best performance of physical mixtures indicates that solid-state/matrix effects can outweigh cavity inclusion	[70]
Teprenone—gastroprotective isoprenoid	α-, β-, γ-CD; 1:2 preparation ratio; kneaded powdered complexes	White fluorescent lamp, ~55 lx, 25 °C/60% RH; first-order kinetics	k: γ-CD 1.5 × 10^−2^, β-CD 3.7 × 10^−2^, control 9.7 × 10^−2^, α-CD 1.2 × 10^−1^	TG, PXRD, FTIR, ss ^13^C NMR, SCXRD and MM2; MCC comparator	Host identity reversed the effect: γ-/β-CD protected, whereas α-CD slightly destabilized	[102]
Teprenone—gastroprotective isoprenoid	β-/γ-CD pseudorotaxane-like supramolecular complexes; guest:CD ≈ 1:2.1–2.3	6000 lx under N_2_; HPLC-UV	Both PLSCs more stable than physical mixtures; γ-CD system showed the lower degradation rate	PXRD, ^1^H NMR, PLSC yield/stoichiometry and DLS	Persistent supramolecular organization and host size influenced protection under deoxygenated conditions	[103]
Metabolic and endocrine agents						
Glimepiride—sulfonylurea antidiabetic	HP-β-CD (DS 5.4); 1:1, K = 778.3 L mol^−1^; ball-milled inclusion compound	4500 lx for 5 and 10 days; HPLC	After 10 days: 95.65 ± 0.61% complexed vs. 90.49 ± 0.78% free and 92.59 ± 0.33% physical mixture	Phase solubility, RSM, PXRD, DSC, SEM, FTIR; dissolution and PK/PD	A statistically useful but relatively modest absolute improvement over an already fairly stable free drug	[104]
6-Propyl-2-thiouracil—antithyroid drug	α-CD; 1:1, K_a_ = 3297.57 M^−1^; coprecipitated solid complex	Sunlight; UV-Vis; dose/duration not reported	Degradation: 21.7% free vs. 15.2% complexed	Job plot, thermodynamics, ^1^H NMR, FTIR, PXRD, DSC/TGA, docking	Modest apparent protection; poorly characterized sunlight limits quantitative interpretation	[105]
Respiratory agents						
Dyphylline—xanthine bronchodilator	β-CD; 1:1, K_a_ = 2.93 × 10^3^ M^−1^; aqueous complex	Visible sunlight, 4 h; UV-Vis at 206 nm	Degradation: 8.70% free vs. 4.80% complexed	^1^H NMR, Job plot, conductivity/surface tension and DFT-related studies	Small absolute loss and non-standardized sunlight mean the protective signal should be interpreted cautiously	[106]
Roflumilast—PDE-4 inhibitor for COPD	α-CD, β-CD, γ-CD, HP-β-CD, Mβ-CD; 1:1 aqueous systems	290 nm; UV-Vis/fluorescence kinetics; HPLC-MS after 30 min	CD addition slowed the photoreaction; fluorescence and degradation were suppressed under N_2_	Phase solubility/thermodynamics, docking and in vitro digestion	Photochemistry was strongly O_2_-dependent; host-specific photoprotection could not be ranked reliably	[107]
Retinoids and dermatological agents						
Acitretin, systemic retinoid for severe psoriasis	HP-β-CD and RM-β-CD; 1:1 aqueous complexes	Fluorescent light (6 × 6 W), pH 9, 37 °C, up to 6 h; HPLC	k 0.457 h^−1^ free → 0.251 (HP-β-CD) and 0.238 h^−1^ (RM-β-CD)	Phase solubility, FTIR, DSC, PXRD and MMFF94s modeling	Both substituted β-CDs provided comparable protection against retinoid photodegradation	[108]
Clobetasol propionate—topical corticosteroid	DPC-crosslinked β-CD nanosponges; β-CD:DPC 1:2–1:10, optimized 1:4	UV fluorescence cabinet, 10 cm, 60 min; UV-Vis at 239 nm; wavelength/irradiance not reported	Optimized nanosponge showed lower photolysis rate than free drug	DLS, SEM/TEM, FTIR, PXRD, DSC/TGA, ^1^H NMR; Carbopol hydrogel used later	Protection is attributable to the complete crosslinked nanosponge environment rather than a simple binary inclusion complex	[109]
Dithranol (anthralin)—photolabile topical antipsoriatic	DPC-crosslinked β-CD nanosponges; optimized 1:4 (solvent evaporation) and 1:6 (melt)	UVA lamp, 10 cm, 60 min; UV-Vis every 10 min	Degradation: 97.4% free vs. 36.14% in optimized nanosponge; melt-prepared NS slightly better	Solubility/loading, DLS, FTIR, DSC, PXRD, SEM/AFM and DPPH	Crosslinking and nanosponge preparation method materially affected photoprotection	[110]
Isotretinoin (13-cis-retinoic acid)—severe-acne retinoid	HP-β-CD (α-CD also studied); 1:1 at pH 7.4–8.1; aqueous complexes	Fluorescent lamps (3 × 6 W), 30 cm, 37 °C, up to 6 h; HPLC	Estimated limiting rate constants: k_free_ = 0.4286 h^−1^ and k_c_ = 0.0612 h^−1^ (~7-fold difference); actual k_obs_ depends on the fraction of complexed drug.	Phase solubility, FTIR, DSC, XRD and MMFF94s modeling	Guest geometry supports shielding/restriction of the photoisomerization-prone polyene chain	[47]
Isotretinoin—oral retinoid	α-CD/soybean-oil freeze-dried beads; no direct isotretinoin/α-CD inclusion stoichiometry	Artificial daylight ~100,000 lx, up to 48 h, air vs. N_2_; HPLC isomer analysis	t_1_/_2_ 2.4 → 4.2 h in air and 2.6 → 6.0 h under N_2_	Bead size/loading, storage stability and pharmacokinetics; soybean oil + N_2_	Protection arose mainly from the bead/oil matrix and oxygen control, not demonstrated cavity inclusion.	[111]
Isotretinoin—topical anti-acne retinoid	HP-β-CD complex loaded into elastic liposomes	366 nm, 30 W, 10 cm, 25 °C, up to 6 h; HPLC	After 6 h degradation: 98.5% free, 47.5% liposomes, 27.9% CD-complex-loaded liposomes	FTIR, TEM, vesicle size/EE, release and Franz-cell permeation/deposition	Drug-in-CD-in-liposome architecture provided incremental protection over liposomes alone	[31]
Tretinoin (all-trans-retinoic acid)—anticancer retinoid	HP-β-CD; 1:1 and 1:2 species; aqueous formulation	Fluorescent light (6 × 6 W), 40 °C, up to 8 h; HPLC; dark controls	Photodegradation slower in HP-β-CD formulation than in methanolic ATRA	Phase solubility, complexation constants and physical-stability studies	Methanolic free-drug and aqueous HP-β-CD formulations were compared; the solvent mismatch prevents attribution of the observed stabilization specifically to CD inclusion.	[112]
Tretinoin (all-trans-retinoic acid)—topical anti-acne retinoid	β-CD; 1:1 complex indicated; 1:1 and 1:3 freeze-dried products	366 nm UV or fluorescent light, up to 30 days; HPLC	After 30 d: 19.7–25.8% complexed vs. ~1% free under UV; 55.2–73.3% vs. 6.6% under fluorescent light	Phase solubility, ^1^H NMR and FTIR	Marked long-term protection, with the magnitude dependent on irradiation regime and preparation ratio	[51]
Tretinoin (all-trans-retinoic acid) -anticancer retinoid/vitamin A metabolite	HP-β-CD; 1:1; fluid-bed-coated inclusion-complex pellets	4500 ± 500 lx for 5 and 10 days; HPLC-UV	After 10 days: 84.08 ± 1.98% complexed vs. 77.02 ± 0.21% free remained	DSC, PXRD, FTIR and solid-state ^13^C NMR; heat/humidity stability	Moderate protection was linked to solid-state shielding of the photosensitive polyene chain	[73]

Several studies provide particularly clear quantitative evidence of pharmaceutical photoprotection. HP-β-CD reduced photodegradation of oxolinic acid by approximately 94% under simulated solar irradiation [43]. In the doxycycline/β-CD system, 96.8 ± 0.9% of complexed drug remained after 6 h of irradiation compared with 68 ± 1% of free doxycycline, and antibacterial activity was better preserved [62]. A similarly clear chromatographic pattern was observed for sufentanil: after 6 h of xenon-light exposure, free drug showed substantial parent loss and additional degradation peaks, whereas the HP-β-CD inclusion complex remained largely unchanged [79].

Preservation of the parent drug, however, represents only one aspect of the potential benefit of CD association. In some systems, complexation also alters photoproduct formation or undesirable biological effects. SBE-β-CD moderately increased bufexamac stability while strongly suppressing photoinduced superoxide generation. Diflunisal provides another example in which reduced parent-drug degradation was accompanied by suppression of a major toxic photoproduct [45]. Biological relevance is particularly clear for chlorpromazine. In an early α-/β-/γ-CD comparison, β-CD most strongly reduced photosensitized skin irritation and shifted photolysis toward the less toxic product promazine [97]. A later study with β-CD and DM-β-CD linked reduced photoallergy to lower skin penetration, radical formation and photochemical binding to albumin, with DM-β-CD showing the strongest overall effect [56]. Thus, the benefit of CD association may extend beyond slower parent-drug loss to reduction in photochemical processes responsible for undesirable biological effects.

Comparative studies also show substantial differences between closely related pharmaceutical guests. The 1,4-dihydropyridine calcium-channel blockers are especially informative. For nifedipine, protection depended strongly on both host identity and irradiation source: DM-β-CD was more effective than HP-β-CD and native β-CD, and all three complexes afforded substantially greater protection under fluorescent light than under sunlight [49]. A broader comparison of eleven DHP anti-hypertensives revealed pronounced guest dependence. Clear photostabilization in the methyl-β-CD systems was observed for amlodipine, felodipine, nisoldipine and nitrendipine, whereas the remaining compounds showed more limited protection; lercanidipine and manidipine also showed particularly low inclusion in the CD system [89]. More focused studies showed that methyl-β-CD approximately doubled the photostability of isradipine without changing the detected photoproduct pattern [92], whereas γ-CD produced only moderate stabilization of manidipine while leaving the principal photodegradation pathway essentially unchanged [93]. HP-β-CD produced a much larger effect for cilnidipine, for which more than 25% of the free drug was lost after 24 h compared with only minor degradation of the complexed drug [91]. However, the original study contains inconsistent temperature information (25 °C vs. 40 °C), which complicates interpretation of the reported photoprotective effect. Temperature can influence both the host–guest association constant (K_a_) and the kinetics of degradation pathways involving thermally activated steps. In equilibrating solutions, temperature-dependent changes in K_a_ may alter the relative fractions of free and complexed drug, thereby affecting the apparent photodegradation rate independently of changes in intrinsic photochemical reactivity. In solid formulations, temperature may additionally influence molecular mobility and other physical-state-dependent processes. Consequently, without clearly documented and controlled sample temperatures during irradiation, the contribution of thermal effects to the observed stabilization cannot be reliably distinguished from that of cyclodextrin complexation. The DHP series therefore illustrates the wide range of responses that can occur even among structurally related drugs.

A similar, though smaller-scale, guest-dependent pattern was observed for the closely related imidazoline drugs naphazoline, xylometazoline and antazoline. β-CD increased HPLC recovery in all three systems, but the magnitude of protection varied markedly, and in the case of antazoline the protective effect was evident chromatographically but not by spectrophotometric kinetics [52].

Host–guest affinity itself is not a reliable predictor of photoprotection. For sulfanilamide, β-CD and HP-β-CD showed similar 1:1 stability constants (~320–330 M^−1^), yet HP-β-CD provided greater drug retention after UVB exposure (57.7% vs. 46.4% with β-CD and 41.9% for free sulfanilamide) [82]. A similar disconnect between association strength and photoprotection was observed for carvedilol: although HP-β-CD showed the higher reported complex-stability parameter, the β-CD complex provided greater protection during UVB exposure [90]. Acitretin provides an additional example: RM-β-CD formed substantially stronger complexes than HP-β-CD, yet the degradation rate constants of the CD-bound drug were nearly identical (0.238 and 0.251 h^−1^, respectively), compared with 0.457 h^−1^ for free acitretin [108].

**Figure 9:**
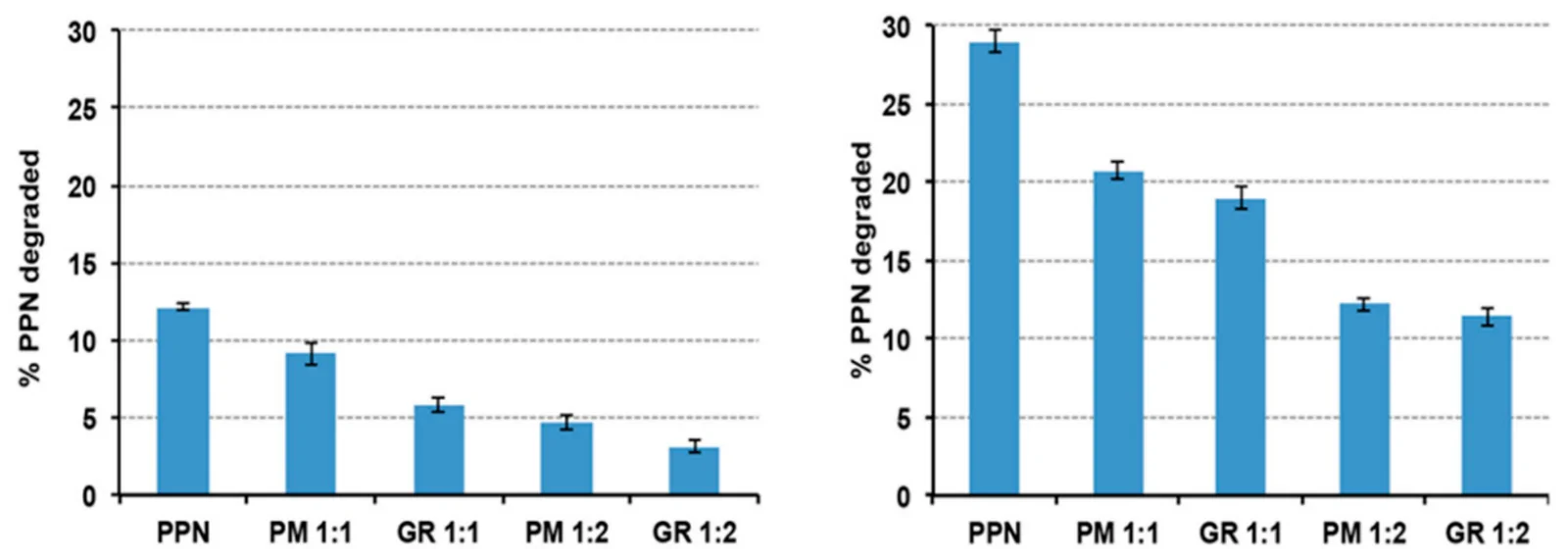
Effect of HP-β-CD proportion and preparation method on propranolol photodegradation. Percentage of propranolol hydrochloride (PPN) degraded after 1 h (**left**) and 5 h (**right**) of UVA/UVB exposure in free PPN and in 1:1 or 1:2 PPN:HP-β-CD physical mixtures (PM) and co-ground systems (GR). Reproduced from Cirri et al. (2023) [96] under CC-BY 4.0.

**Figure 10:**
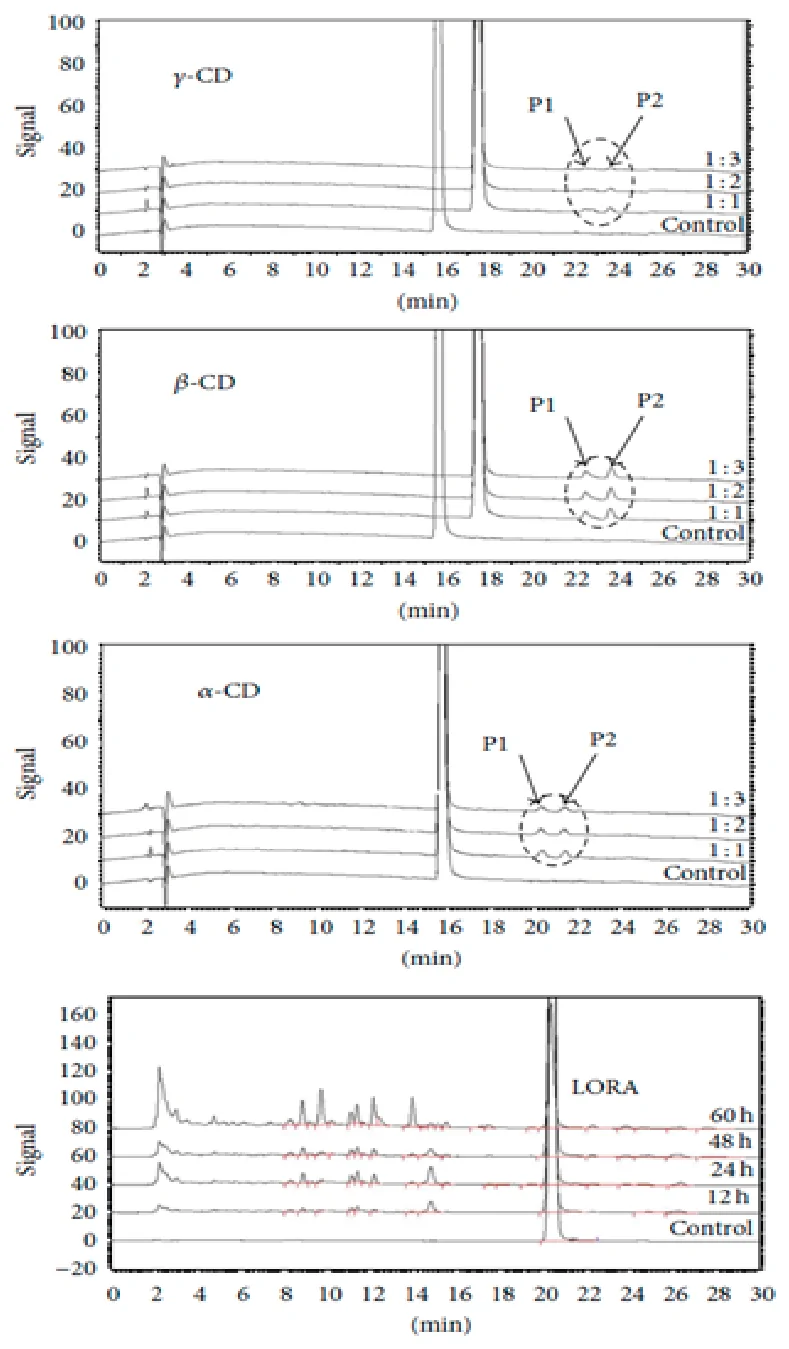
Chromatographic evidence of cyclodextrin-dependent photoprotection of loratadine. HPLC profiles of loratadine and its complexes with α-, β-, and γ-CD after irradiation. For each cyclodextrin, chromatograms of the 1:1, 1:2, and 1:3 drug:CD systems are shown together with the corresponding dark control; P1 and P2 denote photodegradation products detected after irradiation. The lower panel illustrates progressive photodegradation of free loratadine during prolonged light exposure. Reproduced from Rivas-Granizo et al. (2015) [86] under CC BY 4.0.

Photoprotection also depends on CD proportion, preparation method and the resulting organization of the guest–host system. Propranolol illustrates the influence of these variables across two studies. β-CD reduced the apparent photolysis rate by approximately 53–54% in an earlier optical-kinetic study, whereas a later HP-β-CD formulation study showed that both CD proportion and preparation method influenced protection: the 1:2 co-ground system reduced the degradation rate by 73.1% during the first hour, although differences between co-ground and physical-mixture systems largely disappeared over 5 h (Figure 9) [95,96]. Because the two studies used different CD systems and experimental conditions, the absolute effects are not directly comparable. Still, together they show that the observed protection depends on how the guest–CD system is prepared. A broader comparison of doxycycline complexes likewise showed strong dependence on both host identity and CD excess, with γ-CD providing the greatest stabilization and the 1:20 γ-CD system giving the highest drug retention [80]. Loratadine showed particularly clearly that increasing CD content is not necessarily beneficial: 1:1 complexes were consistently more protective than the corresponding 1:2 and 1:3 preparations, with γ-CD 1:1 giving 99.15% drug recovery after 12 h of irradiation (Figure 10) [86]. For indomethacin, β-CD-rich 1:1 and 1:0.5 inclusion products showed fewer irradiation-induced spectroscopic and thermal changes than the low-CD 1:0.1 system and corresponding physical mixtures, although photostability was assessed primarily by non-separative methods [71]. Thus, once a suitable host has been selected, neither increasing the CD proportion nor promoting complex formation guarantees a proportional gain in photostability; preparation and physical organization can be equally important.

Host size can even change the direction of the photostability effect. In kneaded teprenone powders, γ-CD and β-CD reduced the apparent degradation rate relative to the control, whereas α-CD slightly increased it [102]. In a later study, β- and γ-CD pseudorotaxane-like complexes were more photostable than the corresponding physical mixtures under 6000 lx in N_2_, with the γ-CD system providing the stronger protection [103]. Thus, the same guest can shift from slight destabilization to substantial protection depending on host size and supramolecular organization.

**Figure 11:**
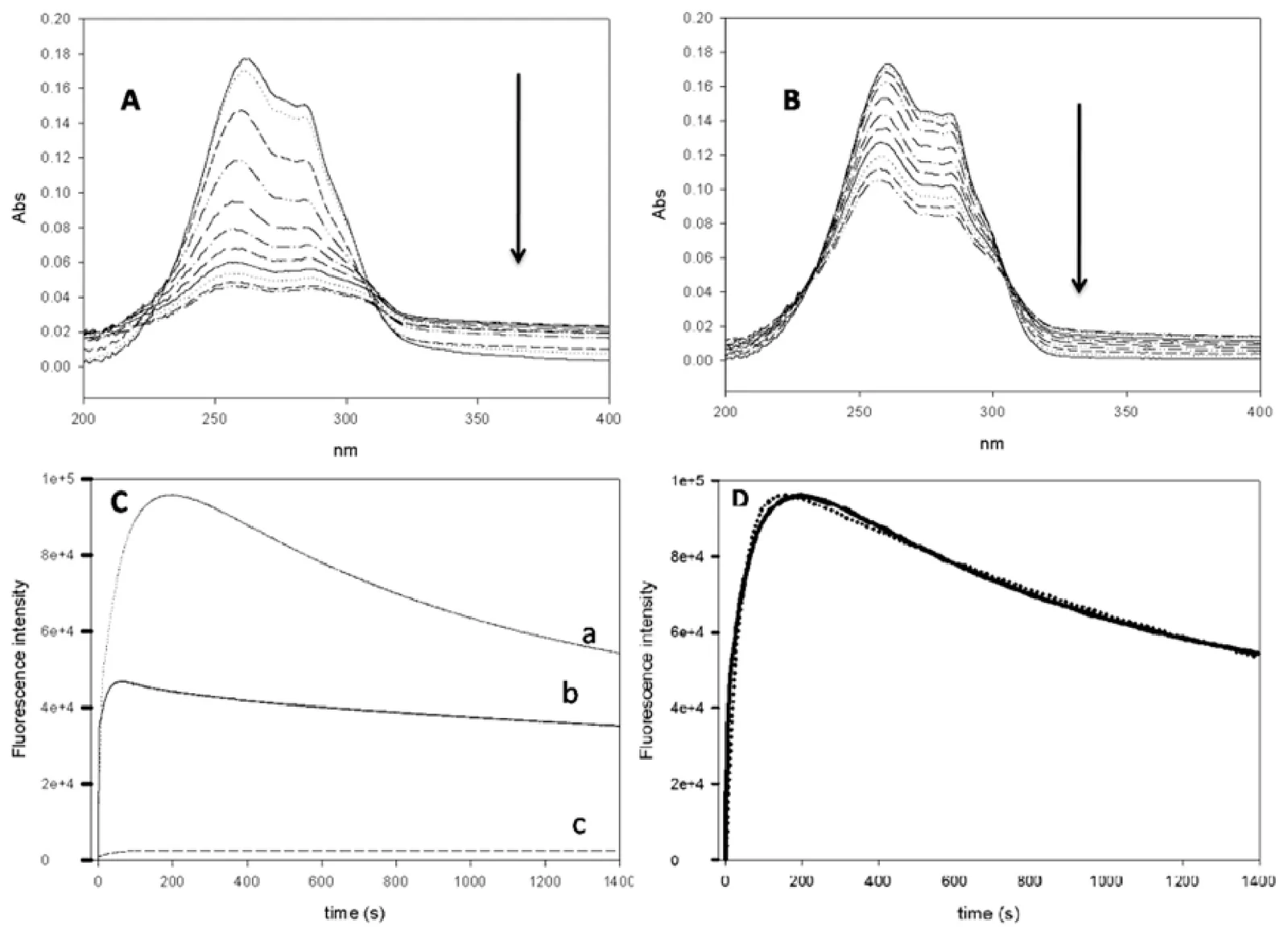
Effect of cyclodextrin association and oxygen availability on roflumilast photochemistry. Time-resolved absorption spectra of roflumilast recorded during irradiation in the absence (**A**) and presence (**B**) of cyclodextrin, together with fluorescence time courses under CD-containing, CD-free and N_2_ conditions (**C**) and kinetic fitting of the fluorescence response (**D**). CD association slowed the irradiation-induced spectral changes, while the fluorescence-generating pathway was strongly dependent on molecular oxygen. Reproduced from Matencio et al. (2019) [107] under CC BY 4.0.

Experimental conditions can further modify the observed effect. As already illustrated for nifedipine, the magnitude of CD-mediated protection can differ substantially with the irradiation source, with greater stabilization reported under fluorescent light than under sunlight [49]. Oxygen availability can also be important. Roflumilast provides an example in which the photochemical response was strongly oxygen-dependent: CD addition slowed irradiation-induced spectral changes, whereas the associated fluorescence response was suppressed under N_2_ (Figure 11) [107]. Serotonin was likewise studied under oxygen-depleted conditions. Under deoxygenated PBS and 254-nm irradiation, 2-HP-β-CD reduced the apparent serotonin photodegradation rate constant from 24.10 to 8.81 × 10^−3^ min^−1^ and increased the half-life from 28.5 to 78.3 min [101]. The substantial effect observed under these oxygen-depleted conditions therefore cannot be assumed to persist to the same extent in air. Irradiation source and oxygen availability can therefore materially influence the apparent photostability outcome.

**Figure 12:**
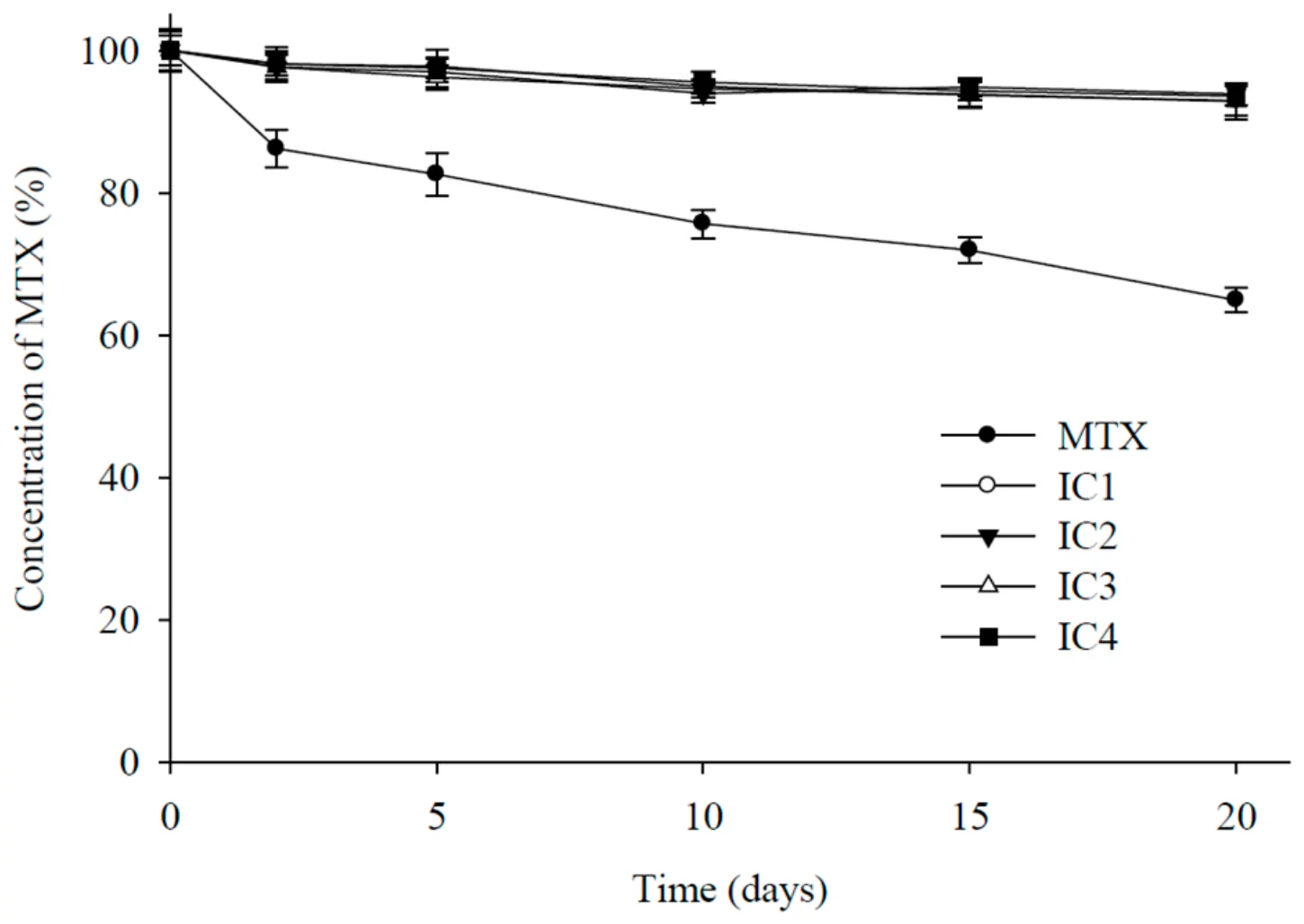
Solid-state photoprotection of methotrexate by β-CD and its derivatives. Residual MTX content during 20 days of forced light exposure for free MTX and spray-dried inclusion complexes with HP-β-CD (IC1), β-CD (IC2), M-β-CD (IC3), and DM-β-CD (IC4). Free MTX showed progressive degradation, whereas all four CD-containing systems retained approximately 93% of the initial drug content after 20 days. Reproduced from Giri et al. (2022) [23] under CC BY 4.0.

Anticancer and immunosuppressive agents include several well-characterized examples of CD-mediated photostabilization. In aqueous solution, HP-β-CD slowed doxorubicin photodegradation, with Lineweaver–Burk analysis yielding an estimated degradation rate constant of 0.0329 h^−1^ for the included drug compared with 0.111 h^−1^ in the absence of HP-β-CD [83]. For methotrexate, a freeze-dried β-CD system remained essentially unchanged during both three months of daylight exposure and a 10-day forced-light study, whereas free MTX lost approximately 40% and showed precipitation and color changes [85]. A later HPLC-based study under controlled light exposure extended this observation across several β-CD derivatives: after 20 days of controlled light exposure, free MTX lost 36.8 ± 1.7%, whereas spray-dried complexes with β-CD, HP-β-CD, M-β-CD and DM-β-CD showed only about 6.7 ± 1.9% loss (Figure 12) [23]. By contrast, β-CD produced only modest apparent protection of 6-mercaptopurine under visible sunlight, with 90.6% degradation of free drug and 75.9% degradation of the complex after 120 min; this result was based on non-standardized sunlight exposure and UV–Vis monitoring [84]. Thus, even within this therapeutic group, reported effects range from robust chromatographically demonstrated stabilization to more modest changes supported by less controlled analytical approaches.

Evidence for several other therapeutic groups is less extensive, and some reported effects are modest or methodologically less secure. For glimepiride, HP-β-CD increased drug retention after 10 days of controlled light exposure from 90.49 ± 0.78% for the free drug to 95.65 ± 0.61%, with HPLC providing direct quantitative evidence [104]. α-CD also produced modest apparent protection of 6-propyl-2-thiouracil, although interpretation is limited by non-standardized sunlight exposure and UV-Vis monitoring [105]. The smaller antiparasitic literature requires similar caution. RM-β-CD slowed the photodegradation of benznidazole while preserving trypanocidal activity and reducing mammalian-cell cytotoxicity [87]. However, the quantitative kinetic data reported for this system require particular caution. The apparent degradation rate constants were given as 9.17 × 10^−1^ and 6.49 × 10^−1^ min^−1^ for free and RM-β-CD-complexed benznidazole, respectively [87]. If interpreted according to first-order kinetics, these values correspond to half-lives of approximately 0.76 and 1.07 min, which are difficult to reconcile with the reported irradiation and sampling period of up to 6 h. This discrepancy suggests a possible error in the reported units or numerical exponents, although the correct interpretation cannot be established unambiguously from the available information. Consequently, while the study suggests a protective effect of RM-β-CD, the absolute kinetic parameters should not be used for quantitative comparisons without further verification. This example illustrates the importance of checking reported rate constants against experimental time scales and the underlying degradation profiles. HP-β-CD similarly slowed the HPLC-monitored photodegradation of hydroxymethylnitrofurazone and reduced its cytotoxicity [88]. However, the original publication contains an internal inconsistency: its abstract describes a destabilizing effect of HP-β-CD on NFOH photodecomposition, whereas the chromatographic results and the accompanying discussion indicate reduced apparent degradation rate constants and improved photostability after complexation [88]. The interpretation of this study should therefore be based primarily on the experimental findings rather than the contradictory statement in the abstract.

Formulation introduces additional variables, particularly when CD-containing systems are transferred from solution into solid products and multicomponent dosage forms. Physical state alone can substantially modify the apparent benefit of complexation. In the nifedipine/β-CD system, protection was observed both in solid samples exposed to daylight and in irradiated solutions, although the stabilizing effect was substantially greater in the solid state [94]. Lansoprazole showed a similar host-dependent effect in fluid-bed-coated pellets, with HP-β-CD providing greater photoprotection than β-CD, consistent with deeper modeled shielding of the photolabile sulfinyl region [75]. In solid ranitidine preparations, β-CD-containing systems reduced volatile photoproduct formation and irradiation-induced color and odor changes, yet some physical mixtures performed as well as or better than freeze-dried complexes [70]. These results indicate that the solid-state environment can contribute substantially to stabilization and may, in some cases, rival the effect of well-defined molecular inclusion.

Retinoids provide a useful cross-formulation example of this interplay. Photoprotection has been reported across all-trans-retinoic acid, acitretin and isotretinoin systems [47,51,108,112], although the underlying comparisons differ considerably in formulation context. Early studies of all-trans-retinoic acid (ATRA) reported improved photostability in HP-β-CD- or β-CD-containing formulations under fluorescent and UV irradiation [51,112]. However, a critical methodological limitation arises in the study comparing free ATRA dissolved in methanol with an aqueous HP-β-CD formulation [112]. This solvent-mismatched experimental design does not permit the observed differences in photodegradation to be attributed specifically to cyclodextrin inclusion. Methanol and water differ substantially in polarity, oxygen solubility and the quenching behavior of photoexcited species. They also provide different singlet-oxygen lifetimes, although the involvement of singlet oxygen in the reported ATRA degradation was not directly established. These solvent-dependent factors may independently alter photochemical reaction pathways, degradation kinetics and photoisomerization behavior. Consequently, the reported comparison demonstrates differences in the photostability of two formulation environments rather than an isolated molecular shielding effect of HP-β-CD. Reliable evaluation of the specific contribution of cyclodextrin would require appropriate solvent- and pH-matched controls, with comparable oxygen availability and irradiation conditions. Where the poor aqueous solubility of free ATRA prevents such matching, the contribution of cyclodextrin inclusion cannot be unambiguously separated from solvent and formulation effects. In solid ATRA/HP-β-CD pellets, the effect was more modest but directly quantifiable: after 10 days of illumination, 84.08% of the complexed drug remained compared with 77.02% of free ATRA, while solid-state ^13^C NMR supported shielding of the photolabile polyene side chain [73]. Similar protective outcomes across the retinoid class can therefore arise under quite different molecular and formulation conditions.

Beyond physical state, the final dosage form and its immediate environment can either reinforce CD-mediated stabilization or reduce its practical importance. Incorporation of a β-CD system into a topical diclofenac gel substantially delayed photodegradation and outperformed both the control gel and the commercial 1% formulation under matched irradiation conditions [76]. Packaging can be equally decisive. For DY-9760e, SBE-β-CD markedly reduced HPLC-defined drug loss and discoloration in clear ampoules, whereas amber glass largely prevented photodegradation even in the absence of cyclodextrin [98]. The apparent contribution of the CD therefore depends on the protection already provided by the complete product.

**Figure 13:**
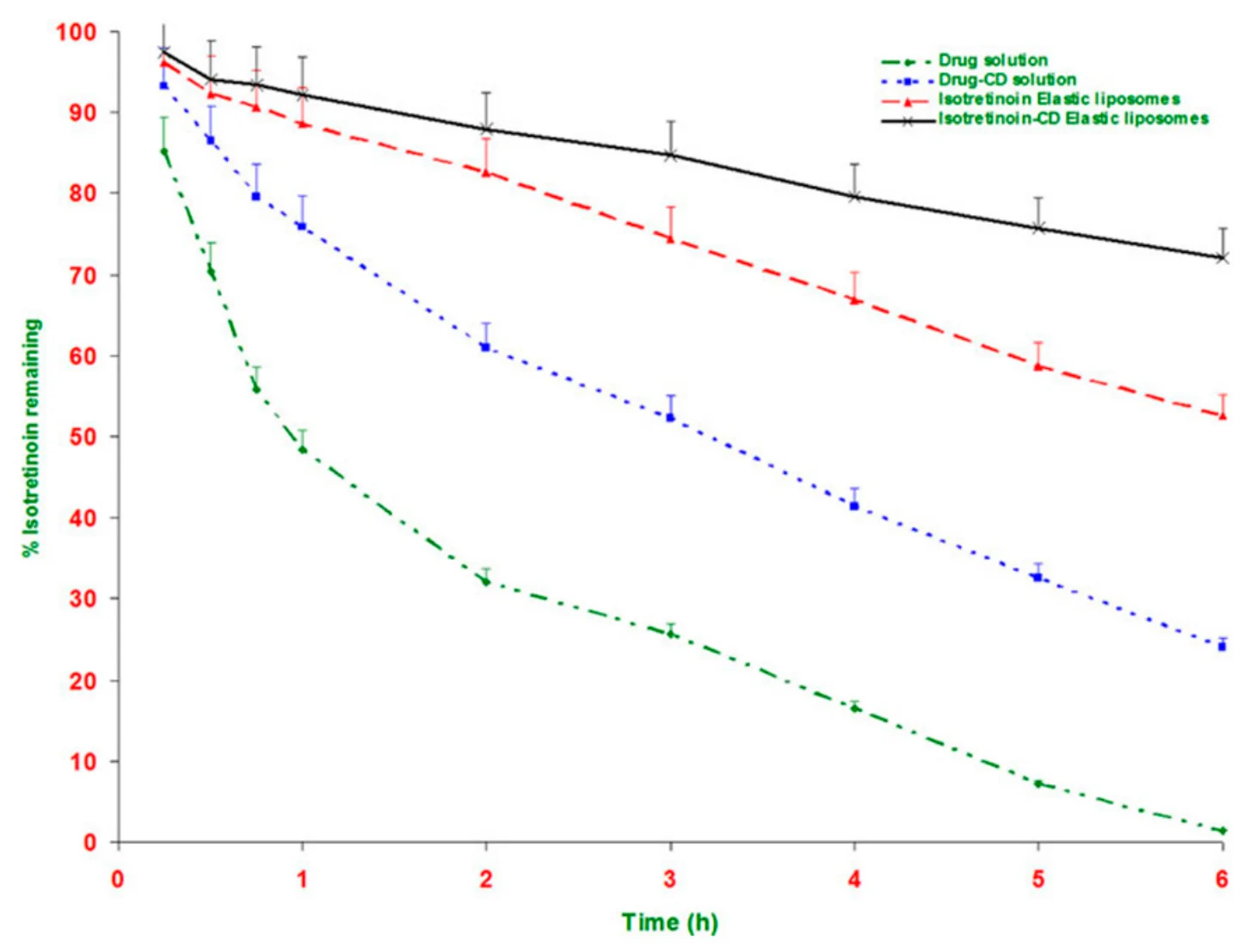
Incremental photoprotection of isotretinoin in a drug-in-CD-in-liposome system. Photodegradation profiles of isotretinoin in solution, elastic liposomes, and HP-β-CD-complex-loaded elastic liposomes during UV exposure. After 6 h, isotretinoin degradation reached 98.5% in solution and 47.5% in elastic liposomes, but only 27.9% in the dual-carrier formulation, demonstrating additional protection when the CD complex was incorporated into the vesicles. Values are reported as mean ± SD (n = 3). Reproduced, with permission, from Kaur et al. (2010) [31].

Advanced carrier systems combine CD association with compartmentalization and other forms of molecular confinement. The optimized caffeine-containing HP-β-CD nanosponge formulation reduced light-induced ergotamine loss from approximately 14% to 2.5% [99]. More elaborate drug-in-CD-in-liposome systems produced additional stabilization of barnidipine and isotretinoin relative to simpler carriers [31,64]. For isotretinoin, the incremental contribution of the combined architecture was particularly clear: after 6 h of UVA exposure, degradation reached 98.5% for the free drug and 47.5% in elastic liposomes, but only 27.9% when the HP-β-CD complex was incorporated into the liposomes, illustrating the additional protection achievable with a drug-in-CD-in-vesicle architecture (Figure 13) [31]. Crosslinked CD nanosponges provide a related approach. Clobetasol propionate encapsulated in β-CD/DPC nanosponges underwent slower UV-induced photolysis than the free corticosteroid [109]. An even larger effect was reported for dithranol, for which degradation after 60 min decreased from 97.4% for the free drug to 36.14% in the optimized nanosponge formulation; importantly, the magnitude of protection also varied with the nanosponge preparation method [110]. In such systems, photostability reflects the combined effects of inclusion, compartmentalization and carrier architecture rather than a simple binary host–guest equilibrium.

Protection can also occur without direct inclusion of the drug in the CD cavity. In α-CD/soybean-oil beads, isotretinoin—too large for the α-CD cavity—showed approximately twofold longer photodegradation half-lives than in soybean oil alone, indicating that the CD-based matrix and oil microdomains can act as a physical light-protective environment [111]. This finding marks an important distinction between molecular inclusion and formulation-level photoprotection: as carrier complexity increases, the observed stabilization increasingly becomes a property of complete architecture. Such systems are considered more broadly in Section 5.

The pharmaceutical examples summarized in Table 1 illustrate how the mechanisms discussed in Section 2 can act individually or in combination. Differences in guest accommodation and exposure of photolabile groups (Section 2.1) help rationalize the variable protection observed for imidazoline derivatives [52] and the modeled retinoid complexes [47]. Changes in intermolecular association and conformational freedom (Section 2.2) have been proposed for oxolinic acid [43], whereas modification of reactive-species accessibility (Section 2.3) and excited-state or radical pathways (Section 2.4) are supported by observations for bufexamac, chlorpromazine and diflunisal [45,55,56]. Conversely, the absence of significant protection for carprofen and ofloxacin [113,114], together with the altered degradation behavior of ketoprofen and midazolam [69,115], illustrates the limitations discussed in Section 2.5. These examples demonstrate that neither strong host–guest association nor inclusion-complex formation guarantees photoprotection. In many pharmaceutical systems, however, the available evidence does not permit identification of a single dominant mechanism, and the proposed explanations should therefore be interpreted with appropriate caution.

### 4.2. Vitamins, Natural Bioactives and Food-Related Compounds

Vitamins, natural bioactives and food-related compounds form a chemically diverse group in which photosensitivity often coincides with poor aqueous solubility, oxidation susceptibility or loss of biological activity during processing and storage. Cyclodextrin complexation can offer several formulation benefits simultaneously, including improved solubility, dispersibility and resistance to light. However, improved solubilization or strong association does not necessarily translate into improved photostability. The available studies cover vitamins, carotenoids, flavonoids, phenylpropanoids, stilbenes, essential-oil constituents and other natural antioxidants and are summarized in Table 2. Across these groups, the most informative studies are those that distinguish protection of the intact compound from changes in isomeric composition or preservation of biological function, because these endpoints do not always respond to CD complexation in parallel.

**Table 2:** Photostability studies of vitamins, natural bioactives, nutraceuticals and food-related compounds in cyclodextrin-containing systems.

Guest/Application	Cyclodextrin Systems	Irradiation/Assessment	Photostability Outcome	Supporting Evidence	Interpretation	Ref.
Vitamins, vitamin analogues and coenzyme-related antioxidants						
Ascorbic acid—vitamin C/water-soluble antioxidant	HP-β-CD; ternary AA/TEA/HP-β-CD system; 1% HP-β-CD with variable TEA	Mercury arc 312–577 nm or fluorescent daylight 400–600 nm, 25 °C; UV-Vis kinetics; validated RP-HPLC/UV assay	Photostability increased ~11–35-fold; the TEA/HP-β-CD system generally outperformed TEA alone	Validated UV-Vis/RP-HPLC; pH and stress studies; TEA comparator/component	A multicomponent example in which CD enhanced stabilization beyond the effect of the auxiliary ligand alone	[116]
Coenzyme Q10—nutraceutical cofactor/antioxidant	β-CD and γ-CD; 1:1; hydrated/lyophilized inclusion complexes with physical-mixture controls	UVC 254 nm, 15 cm, 25 or 80 °C, 120 min; HPLC-APCI-MS; parallel dark thermal controls	At 25 °C, complexes showed little loss vs. 27.7% loss free; at 80 °C + UV, 79.2% (β-CD) and 64.9% (γ-CD) remained vs. 27.7% free	Phase solubility, HPTLC, FTIR, PXRD, DSC/TGA; temperature/pH solubility studies	Dark controls separate thermal from photochemical loss and show host-dependent protection under combined heat/UV stress	[117]
Riboflavin—vitamin B_2_/photosensitive nutrient	β-CD and γ-CD; 1:1 complexes entrapped in dehydration-rehydration vesicle (DRV) liposomes	365 nm UV; fluorescence kinetics and half-life determination	T_1/2_: 0.48 h free → 2.9 h with γ-CD → 9.63 h after γ-CD complex entrapment in DRV; full multicomponent DRV system: 128.3 h	^1^H NMR, fluorescence, derivative UV-Vis, entrapment efficiency; DRV lipids and UV absorbers/β-carotene in full system	Protection increased stepwise with hierarchical organization, but the largest effect belonged to the complete multicomponent carrier	[118]
α-Tocopherol (α-TQ) and α-tocopheryl acetate (α-TQA)—vitamin E forms	β-CD; 1:1 preparation ratio; freeze-dried inclusion complexes; corn-starch complexes as comparators	Visible light and UVC 254 nm; residual compounds by UV-Vis	Both β-CD complexes retained >99%; free α-TQ retained 19.4–61.2% and α-TQA 76.5–89.1%, depending on state/exposure	FTIR, TG/DTG, DSC and molecular docking	β-CD strongly protected both vitamin E forms, while the free compounds showed markedly different intrinsic photosensitivity	[119]
α-Tocopherol—vitamin E antioxidant/topical and food applications	β-CD; α-TC:β-CD 2:1 preparation ratio, A_N-type profile (K_s_ ≈ 102 M^−1^); complex in electrospun PCL nanofibers	UV, 300 W, 65 min; post-irradiation α-TC recovery by HPLC release assay	Post-UV recovery: 97 ± 0% with β-CD vs. 91 ± 1% in matched PCL/α-TC fibers without β-CD	Phase solubility, XRD, SEM, TGA, HPLC release and DPPH; carrier-matched PCL control	The modest but direct incremental benefit over a matched CD-free nanofiber control supports a CD-specific contribution	[120]
α-Tocopherol—lipophilic vitamin/antioxidant	β-CD and β-CD-6-EDA/D400/D2000 derivatives; 2:1 or 1:1 host–guest systems with temperature-responsive release	365 nm UV, 12 h; fluorescence; natural light for 1 month	CD/VE fluorescence was essentially unchanged after UV while free VE decreased; host–guest solutions showed no color change after 1 month	Variable-temperature ^1^H NMR, ROESY, UV-Vis, fluorescence/CD, MS, TEM, DLS, XRD and DPPH	Extended host architectures preserved optical stability, although the principal irradiation endpoint was fluorescence rather than direct chemical assay	[121]
Trolox—water-soluble vitamin E analogue/antioxidant	HP-β-CD; 1:2 preparation ratio, K_s_ = 155 M^−1^; freeze-dried complex in water, HEC gel and O/W emulsions	UVB irradiation; HPLC kinetics	In water, k decreased from 1.13 to 0.46 × 10^−7^ M s^−1^; lower rates also observed in gel/emulsions and TiO_2_ photocatalysis was reduced	Phase solubility, UV-Vis, DSC, membrane/skin permeation and TBARS/MDA; TiO_2_ comparator/component	Protection persisted across several matrices and also moderated TiO_2_ -associated photochemical loss	[122]
Trolox—water-soluble vitamin E analogue/cosmetic antioxidant	Methyl-β-CD (Kleptose Crysmeb^®^); 1:4 preparation ratio; freeze-dried complex in HEC, O/W and W/O/W systems	UVB, 2.6 × 10^−4^ W cm^−2^, up to 120 min; HPLC kinetics	k decreased 0.58 → 0.32 × 10^−7^ M s^−1^ in HEC and 0.19 → 0.10 × 10^−7^ M s^−1^ in O/W; lower k also with TiO_2_	Phase solubility, docking, DSC, membrane diffusion and HPLC; TiO_2_ included as photocatalyst/UV screen depending on matrix	The magnitude of protection depended on formulation environment and interaction with an additional photoactive component	[123]
Vitamin A palmitate—fat-soluble vitamin	β-CD; mainly 1:1 at low CD concentration, possible 1:2 at higher concentration; aqueous and freeze-dried complexes	UVA, λ_max_ ≈ 360 nm, 125 W, 40 cm, 25 °C, 6 h; UV-Vis	UV–Vis absorbance decreased by >60% for free VAP within 5 min and became undetectable after 30 min; the β-CD complex retained ~80% of its initial absorbance after 1 h, with little subsequent change up to 6 h.	Phase solubility, FTIR, TGA/SDTA, oxidative DSC and surface-tension/emulsion studies; squalane in proof-of-concept emulsion	Improved UV–Vis absorbance retention suggests apparent photoprotection, but the non-separative assay cannot distinguish intact all-trans-VAP from geometric photoisomers or other absorbing photoproducts.	[7]
Carotenoids and other lipid-soluble antioxidants						
Astaxanthin—carotenoid antioxidant/topical anti-photoaging	SBE-β-CD; 1:1, K_c_ ≈ 1250 ± 85 M^−1^; AX@SBE-β-CD incorporated into a CS/4-PA/DCA UVA-responsive precursor	UVA 365 nm, 10.0 ± 0.2 mW cm^−2^, up to 8 h; residual AX by HPLC	After 4 h: 14.4% free, 61.3% as AX@SBE-β-CD and 78.4% in the multicomponent precursor	Phase solubility, FTIR, PXRD, DSC, SEM, docking, rheology/Franz diffusion and cell/in vivo studies; chitosan/4-PA/DCA/LAP components	Direct HPLC distinguishes a substantial CD contribution from an additional protective effect of the precursor matrix	[124]
β-Carotene—functional-food carotenoid/antioxidant	Succinate-modified β-CD (SACD); BSAF:SACD 2:1 mass ratio; Pickering emulsion with BSAF-SACD interfacial network	UVC 254 nm, 6 h; extracted β-carotene by UV-Vis at 450 nm	Retention: 90.5% BSAF-SACD emulsion vs. 84.5% BSAF-only and 75.0% Tween emulsion	ζ-potential, FTIR, XRD, TGA, SEM/TEM, microscopy and rheology; BSA fibrils/corn oil	The matched BSAF control shows a modest incremental benefit of SACD within a complex interfacial formulation.	[125]
β-Carotene—carotenoid antioxidant/vitamin A precursor	β-CD nanosponges crosslinked with EPI or HMDI; optimized crosslinker: β-CD 2:1 or 4:1; freeze-dried loaded nanosponges	UVA 360 nm, 15 cm, 3 h; UV-Vis sampling every 30 min	Both nanosponge formulations slowed UVA degradation vs. free β-carotene; quantitative values were reported graphically	Loading, FTIR, DSC, XRD, FE-SEM, DLS, solubilization/release, storage and MTT	Protection was formulation-level and depended on crosslinked architecture; quantitative comparison is limited by graphical reporting	[126]
γ-Oryzanol—rice-bran antioxidant/sunscreen component	DPC-crosslinked β-CD nanosponges; GO:NS 1:1–1:4 *w*/*w*; loaded nanosponges in HEC gel and O/W emulsion	UVA, 6.0 × 10^−4^ W cm^−2^, 10 cm, 60 min; UV-Vis kinetics	k decreased 11.8 → 3.1 × 10^−3^ min^−1^ in HEC and 5.3 → 0.6 × 10^−3^ min^−1^ in O/W emulsion	DSC, XRPD, HPLC, membrane diffusion, DPPH, TBARS/MDA and skin accumulation; DPC/HEC/emulsion matrices	The nanosponge effect remained strong in formulation matrices, showing that architecture and vehicle jointly determine protection	[127]
Flavonoids, curcuminoids and other phenolic bioactives						
Alkannin/shikonin enantiomers—wound-healing/anti-inflammatory bioactives	HP-β-CD; 1:1, K_s_ = 260.5 M^−1^; aqueous inclusion complex	Fluorescent light, 30 cm; HPLC first-order kinetics	k decreased 0.329 → 0.156 h^−1^; maximal stabilization at 15–20% HP-β-CD	Phase solubility, HPLC, Lineweaver-Burk analysis and Franz diffusion	Protection showed an optimum with host concentration rather than a simple unlimited concentration dependence	[128]
Chrysin—natural flavone antioxidant/anticancer bioactive	DPC-crosslinked β-CD nanosponges; β-CD:DPC 1:2, 1:4, 1:8; freeze-dried loaded nanosponges	UVA 315–400 nm, 10 cm, 3 h; UV-Vis at 351 nm	After 120 min: 89.1% (1:8), 84.1% (1:4), 78.9% (1:2) vs. 64.6% free	Loading/release, DPPH, metal chelation, DLS/ζ, DSC, XRPD, FTIR, TEM and MTT; β-CD complex comparator	Increasing crosslinker content strengthened formulation-level protection, emphasizing network architecture rather than cavity binding alone	[129]
Curcumin—natural polyphenol antioxidant/anticancer bioactive	β-CD nanosponges crosslinked with DPC or PMDA; β-CD:crosslinker 1:2–1:8; nanoporous carriers	UVA 320–400 nm, 10 cm; residual curcumin by UV-Vis	PMDA-CDNS increased photostability ~1.7-fold and outperformed DPC-CDNS	DLS/ζ, DSC/TGA, FTIR, PXRD, ^13^C NMR, SEM/AFM, BET, swelling/dissolution and MCF-7 assay	Crosslinker chemistry materially altered photoprotection within otherwise related β-CD nanosponge systems	[130]
Curcumin—natural polyphenol/anticancer bioactive	β-CD nanoparticles crosslinked with tetrafluoroterephthalonitrile; loading ≈ 2.3 CUR per β-CD unit	Light exposure for 5 days; UV-Vis; light source/dose not reported; parallel 170 h dark storage	Loaded CD nanoparticles showed little spectral change while free curcumin nanoparticles lost substantial absorbance; aged formulation retained cytotoxicity	FT-Raman, DLS, SEM, porosity/loading and live-cell/cytotoxicity studies	The advanced carrier appears protective, but incomplete irradiation characterization limits quantitative comparison and CD-specific attribution	[131]
Curcumin—natural polyphenol/nutraceutical	HP-β-CD in zein nanoparticles; optimal zein:HP-β-CD 1:1 mass ratio	UV, 0.15 W m^−2^, up to 120 min; residual curcumin by UV–Vis at 428 nm	Little loss during the first 30 min; after 120 min, Cur-ZHNPs retained more curcumin than free Cur	DLS/ζ, EE/LC, FTIR, XRD, fluorescence, TG/DTG, DPPH, storage/heating and digestion; zein matrix	Component controls support an added benefit of the combined zein/HP-β-CD architecture	[6]
Curcumin and resveratrol—natural polyphenols/transdermal delivery	PMDA-crosslinked β-CD nanosponges in Carbomer 934P hydrogel; β-CD:PMDA 1:2 (CUR) and 1:4 (RES)	UVA 320–400 nm, 10 cm; compound content monitored over time	CDNS hydrogel increased t_1/2_ ~5-fold for curcumin and ~7-fold for resveratrol vs. non-CDNS hydrogel	DLS/ζ, TEM, HPLC, Franz release/permeation, rheology and MCF-7 studies; matched Carbomer hydrogel control	The carrier-matched comparison provides relatively strong evidence for the incremental contribution of the CD nanosponge network	[132]
Ellagic acid—polyphenolic antioxidant/food and nutraceutical use	DPC-crosslinked β-CD nanosponges; β-CD:DPC 1:4/1:6; melt- or microwave-prepared loaded systems	Short-wave UV, 10 cm, 60 min; UV-Vis at 264 nm	Both CDNS slowed loss; melt-prepared EANS1 was more stable than microwave-prepared EANS3	Solubilization, FTIR, FE-SEM, drug association, DLS/ζ and DPPH	Preparation method changed the protective performance of nominally similar crosslinked CD architectures	[133]
Myricetin—flavonoid antioxidant/topical photoprotective bioactive	HP-β-CD/PVP electrospun nanofibers; MYR:HP-β-CD:PVP 1:20:12 *w*/*w*/*w* in optimized formulation	UV, 560 µW cm^−2^, up to 9 days; residual myricetin by HPLC	After 9 days: 94.32 ± 1.94% in dry nanofibers vs. 71.60 ± 2.18% raw powder; dry fibers more stable than solution forms	HPLC, solubility/EE, SEM/DLS/TEM, PXRD, FTIR, ^1^H NMR, Franz and antioxidant/cell assays; PVP matrix	Physical state and polymeric confinement strongly influenced the observed benefit, beyond host–guest association alone	[134]
Quercetin—flavonoid antioxidant	Hypercrosslinked β-CD nanosponges; no discrete stoichiometry	UVA 320–400 nm, 3 h; residual quercetin by UV–Vis	~89–91% remained in nanosponges vs. ~79% free	DLS, TEM, FTIR, Raman, DSC and PXRD; diphenyl-carbonate crosslinked matrix	A moderate protective effect was obtained in a crosslinked carrier rather than a simple binary inclusion system	[135]
Quercetin—flavonoid antioxidant	HP-β-CD; 1:1, A^L^ phase solubility, K ≈ 2.27 × 10^4^ M^−1^; coprecipitated complex	UVB 300 nm, up to 90 min; HPLC-DAD at 370 nm	After 90 min: 85.6% complexed vs. 79.6% free remained	Phase solubility, FTIR, ^1^H NMR, PXRD, DSC and UV-Vis	Direct chromatographic evidence shows a real but modest photoprotective effect despite strong complexation	[136]
Quercetin—flavonoid antioxidant	Et-β-CD; 1:1, K = 9234 M^−1^; freeze-dried inclusion complex	UVA 360 nm, 125 W, up to 24 h; UV-Vis	After 18 h: ~90% complexed vs. ~75% free remained	Phase solubility, DSC, FTIR, FE-SEM, ^1^H/ROESY NMR, docking and ORAC	Structural evidence supports inclusion, with a moderate improvement in long-exposure photostability	[137]
Quercetin—flavonoid antioxidant	Multiple CDs screened; SBE-β-CD complex incorporated into drug-in-CD-in-liposomes (DCLs); predominantly 1:1 complexes	UVC 254 nm, up to 72 h; HPLC	Free quercetin fully degraded by 72 h; >70% remained in DCLs, with ~90% after 8 h	Phase solubility, AFM, particle size, encapsulation/loading and storage stability; Lipoid E80 liposomes	Hierarchical CD-in-liposome organization produced much stronger retention than expected from simple inclusion alone	[138]
Quercetin—flavonoid antioxidant	β-CD and γ-CD aqueous systems; Dextran–solanesol micelles as comparators	UVC 254 nm, 0.12 mW cm^−2^, 23 °C; UV-Vis kinetics	T_1/2_: 4.1 h free, 4.8 h β-CD, 9.0 h γ-CD; Dex18-b-Sol comparator 10.4 h	SEC, FTIR, ^1^H/HSQC/DOSY NMR, TEM, MADLS, SAXS, solubility and ABTS	Host identity was decisive: γ-CD approximately doubled half-life, whereas β-CD provided little benefit	[139]
Rutin trihydrate—flavonoid glycoside/antioxidant	β-CD and HP-β-CD; 1:1, K ≈ 508 and 489 M^−1^; coprecipitated complexes	UVB 300 nm, 14.5 W m^−2^, up to 7200 s; UV-Vis	Loss after 7200 s: 13.6% free, 5.44% β-CD and 2.52% HP-β-CD	Phase solubility, FTIR, ^1^H NMR, PXRD and DPPH; BHT reference	HP-β-CD outperformed β-CD despite very similar association constants, again separating affinity from photoprotection	[140]
Rutin—flavonoid antioxidant	HP-β-CD-stabilized nanocrystals; selected rutin:HP-β-CD 30:60 mg; later HPMC hydrogel	Natural sunlight, aqueous dispersions, 4 weeks; residual rutin by UV-Vis	After 4 weeks: 95.1 ± 3.4% in HP-β-CD nanocrystals vs. 42.7 ± 0.7% free	DLS/ζ, EE, FTIR, DSC, PXRD, SEM, solubility/dissolution, skin and in vivo assays; PEG 200 co-stabilizer	Strong formulation-level protection was achieved, although HP-β-CD, PEG and nanocrystal effects cannot be fully separated	[141]
Sesamol—phenolic antioxidant/food, nutraceutical and cosmetic bioactive	DPC-crosslinked β-CD nanosponges; loaded and lyophilized formulation	UV lamp, 10 cm, 0–60 min; UV-Vis; wavelength/irradiance not reported	After 60 min: 68.46% in CDNS vs. 17.59% free remained	FTIR, DSC, ^1^H NMR, FE-SEM, DLS/ζ, EE, DPPH/anti-tyrosinase and storage studies	Large apparent protection was observed, but incomplete characterization of the irradiation source limits reproducibility	[142]
Silymarin—flavonoid mixture/antioxidant bioactive	DPC-crosslinked β-CD nanosponges; no discrete stoichiometry	UV, 40 W, up to 60 min; residual silymarin by UV-Vis	After 60 min: 76% in nanosponges vs. 63% free remained	FTIR, ^1^H NMR, PXRD, FE-SEM, DLS/ζ and loading/release studies	The nanosponge provided a modest absolute benefit for a multicomponent natural extract measured by a non-separative endpoint	[143]
Stilbenes and hydroxycinnamic-acid derivatives						
trans-*p*-Coumaroyl-secologanoside (comselogoside)—secoiridoid/nutraceutical candidate	β-CD; 2:1 COM:β-CD by Job plot with 1:1/2:1 ions; freeze-dried products across several preparation ratios	UVC 254 nm, up to 24 h; trans/cis isomers and total COM by HPLC-DAD	Trans–cis conversion and total loss slowed in 1:2, 1:1 and 2:1 complexes; total COM remained higher than free	Phase solubility, EE/loading, ESI-UHR-QTOF-MS, ^1^H NMR, Job plot, docking and DFT	CD association altered both isomerization and total degradation, with the magnitude dependent on formulation ratio	[144]
Ferulic acid— phenolic antioxidant/sunscreen agent	α-CD selected (β-, HP-β-, γ-, HP-γ-CD screened); 1:1, K = 1162 ± 139 M^−1^; complexes in O/W emulsion	UVB, 10 MED (300 mJ cm^−2^); HPLC	α-CD gave the highest photostability among tested hosts; no degradation products detected after irradiation	DSC, PXRD, ^1^H NMR, ORAC and release study; O/W matrix	Host screening identified α-CD as the best match, underscoring guest–cavity specificity rather than a generic β-CD advantage	[145]
Ferulic acid—phenolic antioxidant/active food packaging	HP-β-CD; 1:1; inclusion complex incorporated into electrospun gliadin nanofibers	UVC 253.7 nm, 75 W, 60 min; residual FA by UV-Vis	After 60 min: ~76% in gliadin/FA-HP-β-CD fibers vs. ~43% in gliadin/free-FA fibers and ~9% unprotected	SEM, fluorescence microscopy, EE, FTIR, DSC/TGA, XRD, release and DPPH; matched gliadin control	The three-level comparison separates a clear incremental CD effect from additional protection supplied by the fiber matrix	[146]
Ferulic acid—phenolic antioxidant/food preservative/UV absorber	Mono-6-O-feruloyl-β-CD covalent conjugate; 1:1 FA/β-CD inclusion complex as comparator	UVB 290–320 nm, 8 W, 15 cm, up to 36 h; HPLC at 313 nm	After 6 h: ~77% conjugate, ~42% inclusion complex and ~33% free FA remained	UV-Vis, FTIR, fluorescence, ^1^H/^13^C NMR, ROESY, HRMS, FE-SEM, PXRD/TGA	Persistent covalent positioning around the CD scaffold provided stronger protection than reversible inclusion	[147]
Ferulic acid—phenolic antioxidant/food and nutraceutical use	β-CD; FA:β-CD preparation ratios 1:2, 1:1 and 2:1; freeze-dried powders	Simulated sunlight, xenon 500 W m^−2^, 25 °C, 6 days; UV-Vis at 330 nm	Loss after 6 d: 8.53% (1:2), 9.35% (1:1), 11.06% (2:1) vs. 13.69% free	XRD, FTIR, SEM,^1^H NMR, MD, EE/LE, solubility and functional assays	A modest but ratio-dependent benefit shows that formulation stoichiometry can matter even for the same host–guest pair	[148]
trans-Polydatin—stilbene glycoside/antioxidant	β-CD, Me-β-CD and HP-β-CD; 1:1, K_s_ = 798, 1106 and 1308 M^−1^; freeze-dried complexes	UV irradiation, 25 °C, up to 180 min; trans-polydatin by UV-Vis	Loss after 180 min: 54% free, 42% β-CD, 36% Me-β-CD and 28% HP-β-CD	Phase solubility, PXRD, TGA/DSC, SEM, ^1^H NMR/ROESY and antioxidant assays	Protection increased across substituted β-CDs in parallel with the reported association constants in this specific system	[149]
trans-Resveratrol—stilbene antioxidant/nutraceutical	α-CD; resveratrol:α-CD concentration ratio 1:5; aqueous analytical buffer containing SDS and 15% acetonitrile	Broad-spectrum UV 200–400 nm, up to 100 min; UV-Vis	Higher A_320_ with α-CD and absence of the 260-nm degradation band; ANOVA *p* = 0.0662, not statistically significant at α = 0.05 (interpreted as significant by the original authors at 90% confidence).	pH-dependent UV-Vis, photoproduct monitoring and one-way ANOVA; SDS/acetonitrile buffer	Suggestive UV–Vis evidence, but *p* = 0.0662 does not meet the conventional α = 0.05 threshold; prespecification of the 90% confidence level was not documented, raising concerns about potential selective interpretation.	[150]
trans-Resveratrol—stilbene antioxidant/topical chemopreventive bioactive	HP-β-CD; 1:2 preparation ratio, 1:1 complex assumed (K ≈ 3189 M^−1^); freeze-dried complex in HEC/gel/O/W matrices	UVA 320–400 nm, 2.9 × 10^−4^ W cm^−2^, 60 min; HPLC	k decreased in HEC (1.66 → 1.36), gel emulsion (0.71 → 0.65) and O/W emulsion (1.16 → 0.80) × 10^−3^ s^−1^	Docking, phase solubility, DSC, DPPH, Cu^2+^ chelation, TBARS and Franz-cell studies; BHT/liposome comparators	Photoprotection was modest and strongly vehicle-dependent, illustrating the importance of the final formulation matrix	[151]
trans-Resveratrol—polyphenol antioxidant	Tetradecyl-γ-CD used as a solid-lipid-nanoparticle component; no discrete host–guest stoichiometry	UVA, 40 W, 2.8 W m^−2^, 30 min; HPLC first-order kinetics	In aqueous SLN, k decreased 14.8 → 7.42 × 10^−4^ s^−1^; lower rates also observed in HEC and O/W emulsions	DLS/PDI/ζ, DSC, encapsulation efficiency, Franz-cell uptake and MDA/TBA; lipid/surfactant matrix	Amphiphilic CD contributed within a lipid carrier, but the observed stability is a property of the complete SLN formulation	[152]
trans-Resveratrol, trans-pterostilbene and trans-pinosylvin—stilbene antioxidants	HP-β-CD and HP-γ-CD; 1:1 for resveratrol/pterostilbene and 1:2 for pinosylvin; freeze-dried complexes	Fluorescent light, 76 days at 4 or 20 °C; HPLC-DAD; dark controls	At 4 °C, HP-CDs improved trans-isomer retention; HP-β-CD benefit was most evident through day 14; pinosylvin complexes still lost ~30% by day 21	Phase solubility, validated HPLC-DAD, FTIR-ATR, DSC, PXRD and ^1^H NMR; bile-salt micelle comparators	Protection was compound-, temperature- and time-dependent rather than uniformly sustained during long-term exposure	[153]
trans-Resveratrol—stilbene antioxidant/nutraceutical	Carboxymethyl-β-CD (DS ≈ 6.8); covalent Res–CM-β-CD conjugate vs. noncovalent 1:2 inclusion system	UVA 365 nm, 10 cm, up to 360 min; UV-Vis	Absorbance loss after 360 min: 12.54% conjugate, 24.05% inclusion system, 32.15% free Res	^1^H/^13^C NMR, FTIR and UV–Vis	Covalent attachment improved durability beyond reversible inclusion, consistent with more persistent host–guest organization	[154]
trans-Resveratrol—stilbene antioxidant/nutraceutical	β-CD nanosponges crosslinked with DPC (NS-I) or PMDA (NS-II); β-CD:crosslinker 1:4	UVA 320–400 nm, 10 cm, up to 8 h; residual Res by UV-Vis at 302 nm	After 8 h: 31.57 ± 1.00% free, 62.26 ± 1.42% NS-I and 75.35 ± 3.07% NS-II remained	Solubility, CDOCKER, DSC, PXRD, FTIR, SEM, DLS/ζ, dissolution and pharmacokinetics	PMDA-CDNS showed greater apparent photoprotection than DPC-CDNS, highlighting the influence of crosslinker-dependent network properties rather than isolated CD cavity inclusion alone	[155]
Essential oils, flavors and volatile bioactives						
trans-anethole, estragole, eugenol and isoeugenol—flavors/preservatives/antifungal bioactives	α-CD, β-CD, HP-β-CD, RAMEB and CRYSMEB; mainly 1:1; aqueous and freeze-dried complexes with physical-mixture controls	UVC 254 nm, 10 × 15 W lamps, 5–60 min; SH-GC first-order kinetics	Photostability increased 18–44-fold (trans-anethole), 6–18-fold (estragole), 1.5–2-fold (eugenol) and 1.6–3-fold (isoeugenol)	UV-Vis solubility, loading/EE, SH-GC and antifungal assays	Large differences among closely related phenylpropanoids show strong guest-structure dependence of CD effect	[8]
Babchi oil (Psoralea corylifolia)—phytotherapeutic essential oil	DPC-crosslinked β-CD nanosponges; optimized β-CD:DPC 1:4; freeze-dried loaded formulation	UVA 320–400 nm, 40 W, 60 min; UV-Vis at 265 nm; first-order kinetics	k decreased 6.909 → 2.303 × 10^−3^ min^−1^ (~3-fold slower)	GC-MS, loading/solubilization, DLS/ζ, FTIR, thermal/PXRD, microscopy and biological assays	A crosslinked nanosponge substantially slowed loss of a multicomponent essential oil, but the effect is formulation-level	[156]
trans-Cinnamaldehyde—flavor/fragrance compound	Ethylcarbonate-γ-CD and octylcarbonate-γ-CD; 1:1, K = 73 and 31 M^−1^; freeze-dried complexes	UVB 2.6 × 10^−4^ W cm^−2^ in HEC gel, O/W and gel emulsions; HPLC	Both γ-CD alkylcarbonates slowed cinnamaldehyde photodegradation in the tested formulations	Phase solubility, UV-Vis, FTIR, DSC/TGA, membrane diffusion and skin permeation	Modified γ-CD hosts provided protection across multiple topical matrices despite relatively modest association constants.	[157]
Estragole—flavor/fragrance, pure or in basil/tarragon oil	α-, β-, γ-CD, HP-β-CD, RAMEB and CRYSMEB; 1:1 in solution, solid CD:ES 1:1–6:1; freeze-dried complexes	UVC 254 nm, 10 × 15 W lamps, up to 60 min; SH-GC	Free ES undetectable by ~40 min; solid complexes retained 54–78% after 60 min and slowed loss 14–26-fold vs. physical mixtures	SH-GC, UV–Vis, ^1^H NMR, Kf, DSC, FTIR, loading/EE, DPPH and release; essential-oil matrices	Strong protection depended on solid complexation and host ratio, with natural matrices providing an additional realistic context	[158]
Muscone—natural musk bioactive/oral-delivery candidate	γ-CD-based MOF; MUS:γ-CD ≈ 1:10 loading ratio; solvent-free loaded γ-CD-MOF	Light exposure for 7, 14 and 30 d; residual muscone by GC-MS; spectrum/intensity not reported	After 30 d: 82.1 ± 1.1% in γ-CD-MOF vs. 58.5 ± 0.7% free remained	GC-MS, SEM, PXRD, FTIR, TG-DSC, porosity, solubility/release, docking/MD; potassium-coordinated MOF	The porous γ-CD framework improved long-term retention, but incomplete light characterization limits cross-study comparison	[159]
Nerolidol (cis/trans/mixture) and cabreuva oil—sesquiterpene flavor/antimicrobial bioactive	α-, β-, γ-CD, HP-β-CD, RAMEB, CRYSMEB, SBE-β-CD; predominantly 1:1; aqueous and freeze-dried systems	UVB 310 nm, up to 8 h; HPLC first-order kinetics	After 120 min: ~17% without CD, ~60% with 0.5 mM and ~90% with 10 mM HP-β-CD; at 1 mM: SBE-β-CD > HP-β-CD > β-CD > α-CD	Phase solubility, UV-Vis, TOC, ^1^H/ROESY NMR and modeling; cabreuva-oil matrix	Both host identity and CD concentration strongly controlled the magnitude of photoprotection	[160]
(+)-Nootkatone—flavor/fragrance/insect-repellent bioactive	β-CD (HP-β-CD screened); 1:1 by ITC; photostability at β-CD:NO 1:1, 2:1 and 5:1	UVB 280 nm, 10 × 15 W lamps, 60 min; SH-GC first-order kinetics	T_1/2_ increased 13 → 18, 26 and 46 min at 1:1, 2:1 and 5:1; after 60 min, 37% remained at 5:1 vs. ~4% free	Phase solubility, ITC, ^1^H/DOSY/ROESY NMR, modeling, loading/EE, TG-DTA and release	Increasing CD excess progressively increased protection, consistent with a larger protected guest fraction	[161]
Star anise alcohol extractives (SAAE; mainly trans-anethole)—flavor/preservative extract	HP-β-CD selected (α-, β-, γ-CD screened); host:guest preparation ratio 1:1.5; freeze-dried complex	UV 8 W, fluorescent light 8 W or dark; 24 h at 30 °C; retention assay	Complex retention ~95% dark, ~62% UV and ~74% fluorescent light; free SAAE was almost completely lost	Phase solubility, GC–MS, UV-Vis, FTIR,^1^H NMR, DSC/TGA, SEM/TEM, electronic nose and modeling	Formulation strongly retained a multicomponent extract, although no unique host–guest stoichiometry can be assigned to the mixture	[162]
Star anise essential oil (mainly trans-anethole)—flavor/antioxidant/food-preservation agent	HP-β-CD; 1:1, K_c_ ≈ 724.5 L mol^−1^; freeze-dried inclusion complex; xanthan coating studied separately	UV, natural light and dark controls; free oil assessed at 2 h, complex at 24 h; UV-Vis	Relative to dark controls, free oil retained ~40% under UV and ~30% under natural light after 2 h; complex retained 82.9% and 100.8% after 24 h	Phase solubility/thermodynamics, microscopy/TEM, TG/DTA, storage/release and food-coating assays	The complex appears highly protective, but unequal exposure durations for free and complexed oil preclude a strictly matched quantitative comparison	[163]

Vitamins provide several well-defined examples of substantial stabilization. For riboflavin, γ-CD increased the UV-induced half-life from 0.48 to 2.9 h, with further protection obtained after incorporation of the complex into dehydration–rehydration vesicles [118]. Entrapment of the γ-CD complex in these vesicles increased the half-life to 9.63 h, whereas the complete multicomponent system containing additional light absorbers and β-carotene reached 128.3 h. This stepwise increase helps distinguish the contribution of inclusion from the additional protection provided by the surrounding carrier. Vitamin A palmitate (VAP) also showed markedly improved retention of its characteristic UV–Vis absorption following β-CD complexation: the signal of the free vitamin decreased by more than 60% within the first 5 min of UVA irradiation and became undetectable after 30 min, whereas the complex retained approximately 80% of its initial absorbance after 1 h, with little subsequent change during the remainder of the 6 h experiment [7]. However, this result should not be interpreted as direct quantitative evidence of preservation of the intact parent ester. Conventional, non-separative UV–Vis measurements at the absorption maximum cannot reliably distinguish all-trans-VAP from geometric photoisomers, including potential 9-cis and 13-cis species, or other structurally related photoproducts with overlapping absorption bands. Consequently, retention of the characteristic absorption signal may reflect preservation of the retinoid chromophore rather than chemical integrity of the original molecule. The formation and relative abundance of individual photoproducts were not established by the reported spectrophotometric measurements. Confirmation of genuine chemical photoprotection would therefore require a stability-indicating chromatographic method capable of resolving the parent ester from its photoisomers and other degradation products.

**Figure 14:**
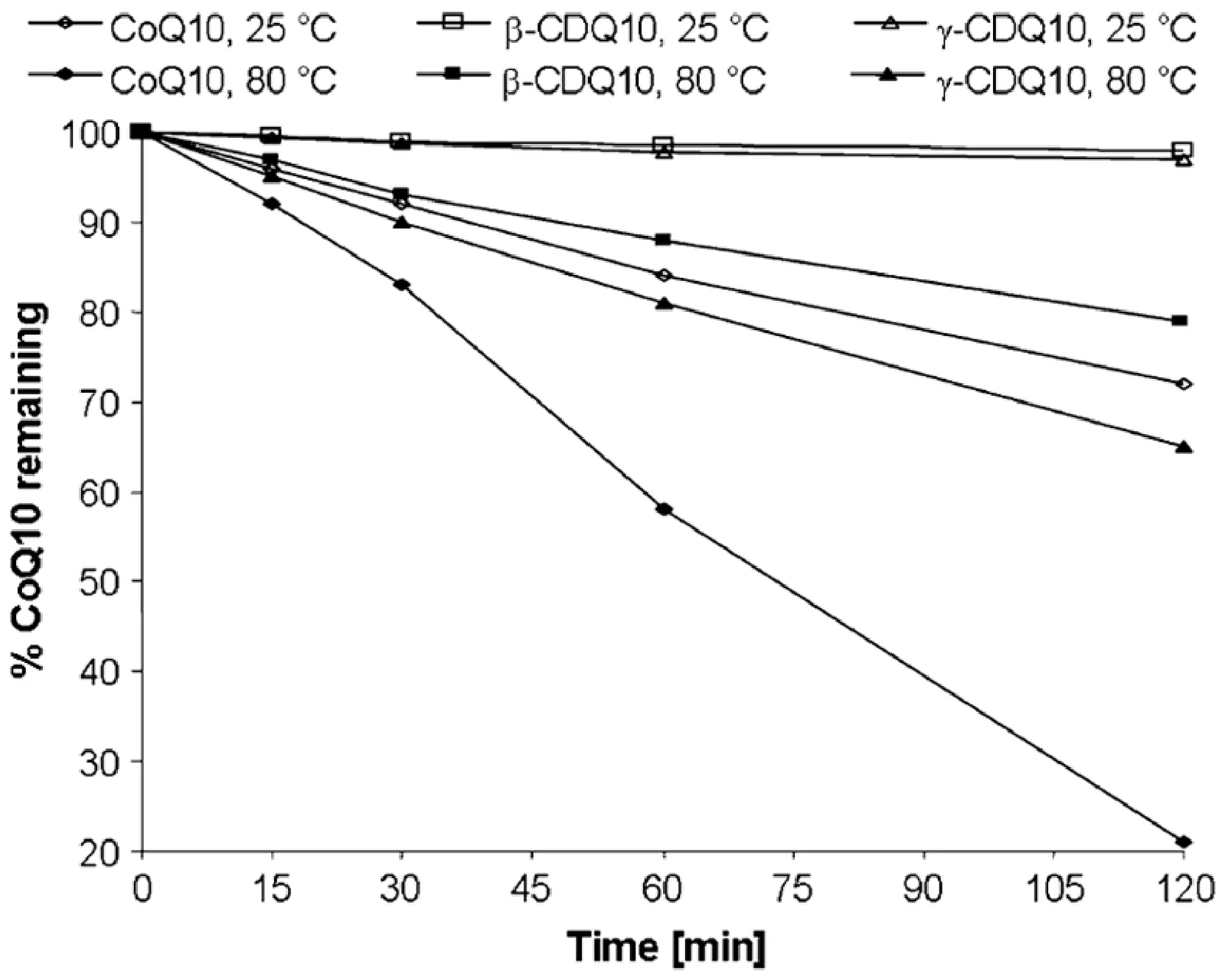
Photodegradation profiles of free coenzyme Q10 (CoQ10) and its β-CD and γ-CD complexes during UVC irradiation at 25 and 80 °C. Complexation markedly reduced CoQ10 loss, with the difference between the two hosts becoming most evident under combined thermal and photochemical stress. Reproduced, with permission, from Milivojevic Fir et al. (2009) [117].

Coenzyme Q10 provides an additional example in which parallel dark thermal controls helped distinguish photochemical from thermal degradation; both β-CD and γ-CD reduced light-dependent loss under UVC exposure (Figure 14) [117]. At room temperature, free CoQ10 lost 27.7% after 120 min, while the CD complexes showed little degradation; under the combined stress of UVC and 80 °C, 79.2% and 64.9% remained in the β-CD and γ-CD complexes, respectively, compared with 27.7% of free CoQ10. Protective effects have also been reported for ascorbic acid and vitamin E derivatives, although the strength of evidence varies with the analytical method and formulation complexity [116,119,120,121]. For example, the ternary ascorbic acid/triethanolamine/HP-β-CD system increased photostability by approximately 11–35-fold depending on the light source and composition [116]. In a carrier-matched α-tocopherol system, post-UV recovery was 97% in β-CD-containing PCL fibers compared with 91% in matched CD-free fibers [120]. β-CD complexes of α-tocopherol and α-tocopheryl acetate also retained more than 99% under the tested light conditions, although free and complexed samples differed in physical state [119]. Trolox provides a complementary example in which CD-mediated protection persisted across aqueous and semisolid matrices but varied with vehicle composition and with the presence of photoactive TiO_2_ [122,123].

Natural phenolic and aromatic compounds show strong guest- and host-dependent behavior. Kfoury and co-workers found large differences among structurally related phenylpropanoids: CD-mediated protection was pronounced for trans-anethole and estragole but much smaller for eugenol and isoeugenol [8]. A similar variability is seen among flavonoids. Quercetin showed only modest protection with HP-β-CD, but substantially greater stabilization with ethylated β-CD and, in a more recent comparison, a longer photochemical half-life with γ-CD than with β-CD [136,137,139]. Rutin provides an especially useful comparison because β-CD and HP-β-CD had similar reported stability constants, yet after UVB exposure the loss of rutin was 5.44% and 2.52%, respectively, compared with 13.6% for the free compound [140]. Thus, similar association constants did not result in the same degree of photoprotection. The same principle is seen for alkannin/shikonin, where the greatest stabilization occurred within an intermediate HP-β-CD concentration range rather than increasing continuously with CD concentration [128]. Host concentration and physical state were also important for volatile bioactives. After 120 min of UVB exposure, approximately 17% of nerolidol remained without CD, compared with about 60% in 0.5 mM and 90% in 10 mM HP-β-CD [160]. In the solid state, freeze-dried estragole/CD complexes retained approximately 54–78% of the guest after 60 min of UVC irradiation and degraded 14–26-fold more slowly than the corresponding physical mixtures [158].

Stilbenes illustrate a related but distinct problem because irradiation can induce photoisomerization as well as irreversible degradation. In the trans-resveratrol/α-CD system, UV–Vis measurements suggested reduced photoisomerization, but the reported statistical evidence requires careful interpretation [150]. The original authors reported an ANOVA *p*-value of 0.0662 and considered the difference significant at a 90% confidence level. However, this result does not meet the conventional α = 0.05 significance threshold, and the study did not establish whether the less stringent α = 0.10 criterion had been specified before the analysis. Although using a 90% confidence level is not inherently inappropriate when justified prospectively, selecting a more permissive threshold after examining the data would increase the risk of false-positive conclusions and confirmation bias. Therefore, these findings should be regarded as suggestive rather than statistically conclusive evidence of photoprotection. Interpretation is further limited by the use of UV–Vis spectroscopy in an analytical medium containing SDS and acetonitrile, rather than direct chromatographic quantification of the individual resveratrol isomers. HP-β-CD produced modest additional protection in gels and emulsions, with magnitude depending on the surrounding formulation [151]. Similar host- and structure-dependent behavior has been reported for polydatin and related stilbenes [149,153]. For trans-polydatin, loss after irradiation decreased from 54% for the free compound to 42%, 36% and 28% in the presence of β-CD, Me-β-CD and HP-β-CD, respectively [149].

**Figure 15:**
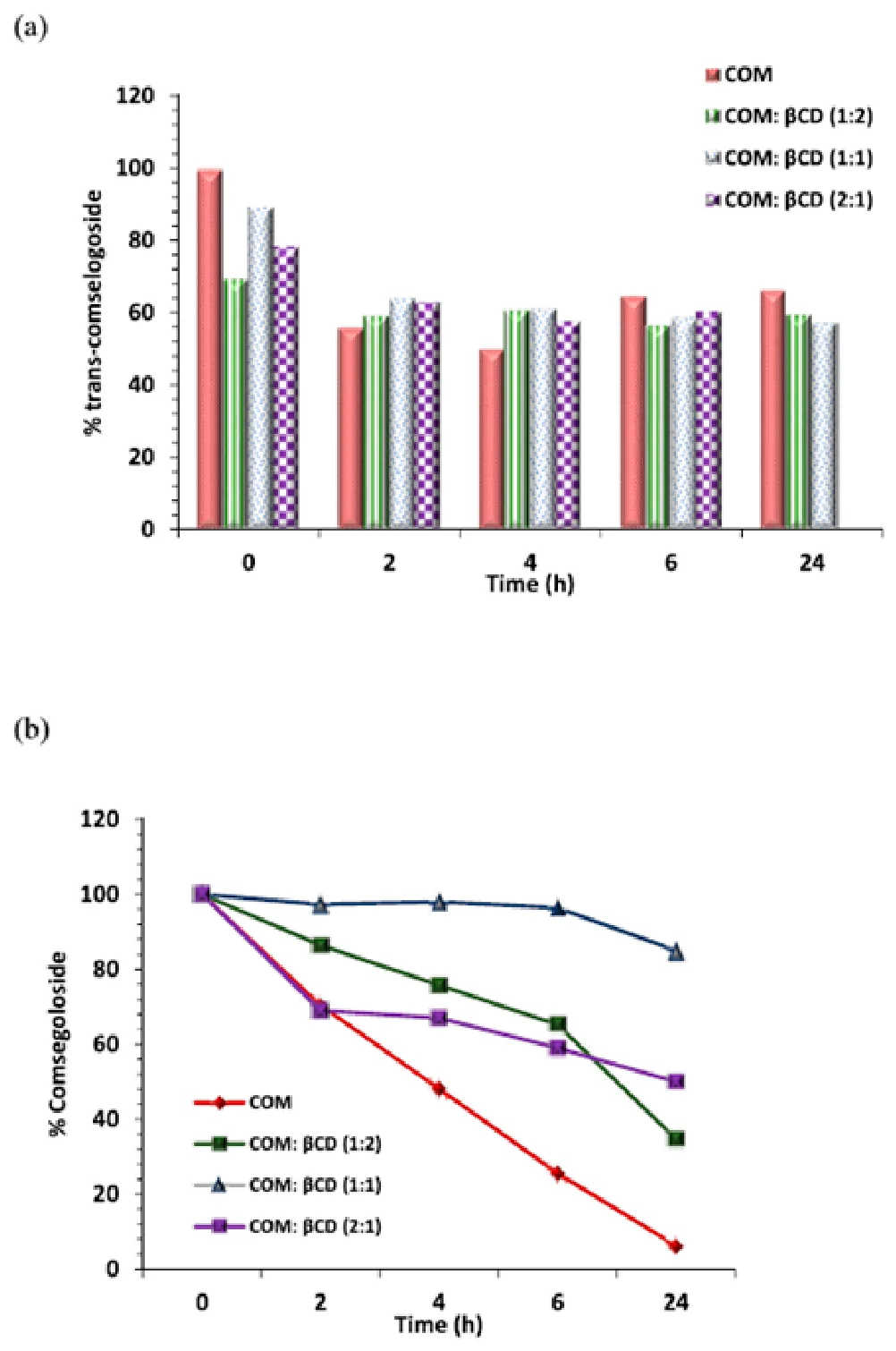
Effect of β-CD complexation on the photochemical behavior of comselogoside (COM) during UVC irradiation: changes in trans/cis isomer distribution and total COM retention in free and complexed samples prepared at different COM:β-CD ratios. Free COM and the β-CD formulations were irradiated in different solvent environments. Reproduced from Rubio-Senent et al. (2024) [144] under CC BY 4.0.

More recently, comselogoside provided a particularly instructive example because trans–cis conversion and total compound loss were followed separately. β-CD slowed both processes, but neither was completely suppressed, showing that retention of the total compound does not necessarily mean preservation of the original isomeric form (Figure 15) [144]. The magnitude of the effect should nevertheless be interpreted cautiously because free comselogoside and the β-CD formulations were irradiated in different solvent environments. For compounds undergoing photoisomerization, both total retention and isomeric composition are therefore relevant.

**Figure 16:**
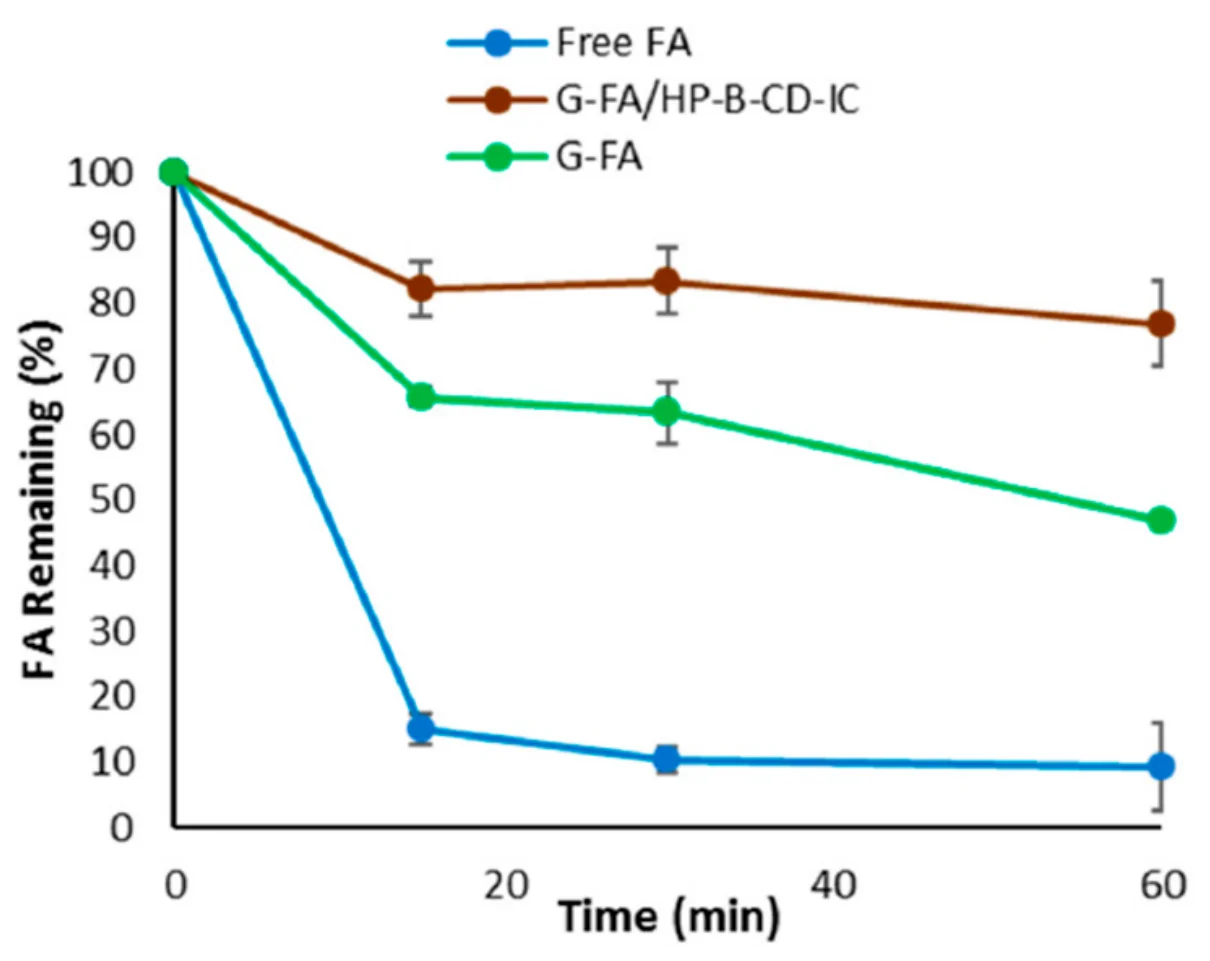
Photostability of ferulic acid (FA) during UVC exposure in the free state, after incorporation of free FA into electrospun gliadin fibers, and after incorporation of the FA/HP-β-CD inclusion complex into the same fibrous matrix. The three-level comparison distinguishes protection provided by the gliadin matrix from the additional effect associated with CD complexation. Reproduced from Sharif et al. (2018) [146] under CC BY 4.0.

A growing proportion of the literature involves more complex CD-containing architectures, including nanosponges, nanofibers, lipid carriers and composite nanoparticles. Several studies allow the contribution of individual components to be separated experimentally. In electrospun gliadin fibers, only about 9% of unprotected ferulic acid remained after 60 min of UVC irradiation, compared with 43% when free ferulic acid was incorporated into the fibers and 76% when the FA/HP-β-CD complex was incorporated into the same matrix (Figure 16) [146]. The matched carrier control therefore shows that both the fibrous matrix and CD complexation contributed to protection. The mode of association with the CD scaffold can also matter—after 6 h of UVB exposure, approximately 77% of a covalent feruloyl-β-CD conjugate remained compared with about 42% of the corresponding noncovalent inclusion complex and 33% of free ferulic acid [147]. A carrier-matched comparison with β-carotene likewise separated the contribution of the CD-containing component: after 6 h of UVC exposure, retention was 90.5% in a BSAF-SACD Pickering emulsion compared with 84.5% in the corresponding BSAF-only system and 75.0% in a Tween-stabilized emulsion [125]. In a recent astaxanthin system, retention after 4 h of UVA irradiation increased from 14.4% for free astaxanthin to 61.3% after SBE-β-CD complexation and to 78.4% after incorporation of the complex into a chitosan-based precursor [124]. These comparisons show that both CD complexation and the surrounding carrier can contribute to the final photoprotective effect.

Curcumin provides an important counterexample to the assumption that strong complexation and improved solubility should result in photoprotection. Several CDs markedly improved its hydrolytic stability while simultaneously reducing its stability under irradiation [50]. Comparison of related curcuminoids further showed that the same HP-β-CD host could stabilize some derivatives while shortening the photochemical half-life of others [26]. For curcumin itself (C-4), the photochemical half-life decreased from 26.2 min in methanol and 12.8 min in buffer/methanol to 7.8 min in 10% HP-β-CD, whereas other members of the same series responded differently. Pronounced photolability was also observed in MβCD-containing curcumin solid dispersions with HA and/or HPMC: after ICH Q1B irradiation, the lyophilizates contained only 0.1 ± 0.1% (*w*/*w*) curcumin, whereas the dark controls remained unchanged [164]. In the intended antimicrobial photodynamic therapy application, however, light responsiveness is functionally required, emphasizing that storage photostability and useful photochemical activity are different endpoints. Conversely, β-CD nanosponges and other higher-order carriers have produced net formulation-level protection of curcumin [6,130,131,132]. These results are not necessarily contradictory: complexation can alter intrinsic guest photochemistry in one direction while the surrounding matrix changes effective light exposure, oxygen availability or molecular mobility in another. The curcumin literature therefore illustrates particularly well that dark stability, binding affinity and photostability are distinct properties and that the photochemical outcome depends on the complete guest–host–environment relationship.

Chemical and functional preservation should also be distinguished in this application area. Some studies evaluate irradiated CD formulations primarily through antioxidant activity, color retention, antimicrobial performance or related functional measures rather than direct determination of the intact compound. For example, vitamin E/HP-β-CD nanofibers retained antioxidant activity after UV exposure even though the remaining vitamin was not independently quantified [165]. Such observations are useful for demonstrating retained function but provide weaker evidence for chemical photostability. For natural mixtures and antioxidants in particular, preserved biological activity does not necessarily demonstrate preservation of the original chemical composition. Functional assays and direct chemical analysis therefore provide complementary information.

For vitamins and natural bioactives, the findings summarized in Table 2 indicate that photoprotection may arise from both molecular and formulation-level effects. Differences in guest accommodation (Section 2.1) are consistent with the distinct responses of quercetin and rutin to different CD hosts [136,137,139,140], while restriction of conformational changes and photoisomerization (Section 2.2) may contribute to the behavior of stilbenes and comselogoside [144,149,150]. Altered local solvation and accessibility of reactive species (Section 2.3) may also be relevant, although these contributions were not directly distinguished in most studies. In multicomponent systems, such as ferulic-acid-loaded gliadin fibers [146], protection additionally reflects the surrounding carrier and cannot be attributed exclusively to cavity inclusion. The adverse or negligible responses of curcumin and related curcuminoids [26,50,164] further illustrate the failure modes described in Section 2.5. Consequently, the proposed mechanism must be considered together with formulation composition and experimental controls, while chemical retention, isomeric composition and functional activity should remain distinct measures of photoprotection.

### 4.3. Sunscreens and Cosmetic UV Filters

Organic UV filters are an instructive group because their intended function requires efficient absorption of UV radiation, while the resulting excited states may undergo photoisomerization, fragmentation or other reactions that reduce filter performance. An effective CD-based strategy should preserve the useful absorption properties of the chromophore while limiting its subsequent photochemical transformation. For this reason, parent-filter retention alone is not always sufficient to establish successful stabilization; preservation of the relevant absorption spectrum, isomeric form and topical performance may be equally important. Avobenzone is the most extensively studied example, although protection has also been reported for aminobenzoate-, camphor-, salicylate- and other UV-filter systems. The available studies are summarized in Table 3.

**Table 3:** Photostability of sunscreen agents and cosmetic UV filters in cyclodextrin-containing systems.

Guest/Application	Cyclodextrin Systems	Irradiation/Assessment	Photostability Outcome	Supporting Evidence	Interpretation	Ref.
Avobenzone and avobenzone-containing systems						
Avobenzone (BMDBM)—organic UVA filter	HP-β-CD (MS ≈ 0.6; other native/HP-CDs screened); BMDBM:HP-β-CD = 1:2; K_1_ ≈ 2233 M^−1^, K_2_ ≈ 13 M^−1^; vacuum-dried complex in solution/O/W lotion	Xenon solar simulator, 4 h; HPLC + GC-MS photoproduct analysis	BMDBM loss: 70.4 ± 6.6% → 49.2 ± 3.4% in solution and 26.6 ± 1.7% → 17.3 ± 3.8% in lotion; physical mixture showed no meaningful protection	Phase solubility, induced CD, DSC, PXRD, HPLC and GC-MS; O/W lotion matrix	Protection depended on true complex formation and was attenuated by the formulation environment	[166]
Avobenzone (BMDBM)—organic UVA filter	HP-β-CD; 1:2 complex loaded into tristearin/phosphatidylcholine lipospheres and O/W cream	Suntest CPS^+^ simulated sunlight, 500 W m^−2^, 2 h (~10 MED); HPLC immediately and after 3 months	Initial loss: 28.9 ± 3.4% free, 17.3 ± 3.9% CD complex, 15.2 ± 2.8% combined carrier; after 3 months: 13.4 ± 2.8% combined vs. 22.1–23.2% single carriers	PXRD, DSC, microscopy, SEM, particle size, encapsulation and release; BHA/EDTA present in common cream base	Drug-in-CD-in-lipid architecture provided incremental protection beyond either carrier alone	[167]
Avobenzone—organic UVA filter	HP-β-CD (MS ≈ 0.8); aqueous formulations with 0–30% HP-β-CD	UVA 320–400 nm, 100–500 kJ m^−2^; HPLC + UV-Vis; in vivo mouse photoprotection	At 500 kJ m^−2^, ~79.3% remained with 30% HP-β-CD vs. ~16.6% without CD; skin penetration, edema and sunburn-cell formation also decreased	DSC, solubility, HPLC, Franz-cell permeation and histology	Protection increased with HP-β-CD concentration and was accompanied by improved topical photoprotection	[25]
Avobenzone—organic UVA filter	2-HP-β-CD (DS ≈ 4.2); AVB:CD = 1:2; K_a_ ≈ 1.4 × 10^5^ (mol L^−1^)^−2^; freeze-dried complex	UV irradiation, up to 200 min, 30 °C, 5% ethanol/water; UV-Vis	Photolysis decreased from 49% free AVB to 8% for the inclusion complex	Phase solubility, FTIR, XRD, TG/DSC, SEM, ^1^H NMR and 2D ROESY	A structurally supported binary inclusion complex produced strong photoprotection under hydroalcoholic conditions	[168]
Avobenzone + octinoxate—UVA/UVB filters	β-CD; crystalline (β-CD)_2_·AVO and (β-CD)_3_·OCT_2_ inclusion complexes prepared mechanochemically	UV irradiation, 24 h; ESI-MS/MS + UV-Vis	No characteristic AVO/OCT photoproducts detected after 24 h in the β-CD complexes; unprotected filters showed pronounced spectral degradation	SCXRD, PXRD, ^1^H NMR, Raman, ATR-FTIR, TGA, DSC and hot-stage microscopy	Defined crystalline host–guest organization strongly suppressed photochemical transformation of both filters	[10]
Avobenzone—organic UVA filter	β-CD (HP-β-CD used in phase-solubility comparisons); predominantly 1:1 in dilute solution; photostability at AVB:β-CD = 1:50–1:100	UVA1 365 nm, 15 W, 15 cm, up to 168 h; UV-Vis, RP-HPLC/SVD + outdoor solar exposure	The most favorable UVA1 protection was reported at AVB:β-CD ratios of 1:50–1:100; elevated β-CD concentrations caused pronounced spectral changes attributed to aggregation of AVB/β-CD-containing species.	Phase solubility, logP, pK_a_, ^1^H NMR titration, solvent-cavity modeling and tautomer analysis	Concentration-dependent behavior was linked to tautomeric redistribution and possible complex aggregation; a water-activity mechanism and accelerated degradation at higher CD concentrations were not directly established.	[60]
p-Aminobenzoate-derived UVB filters						
2-Ethylhexyl *p*-dimethylaminobenzoate (EH-DMAB)—PABA-derived UVB filter	HP-β-CD (native/other HP-CDs screened); 1:1 solid complex tested in solution and O/W emulsion	200 W xenon-mercury lamp, λ > 290 nm, 4 h; HPLC + GC-MS	Degradation decreased from 54.6% to 25.5% in solution and from 33.4% to 25.1% in O/W emulsion	Phase solubility, DTA/TGA, ^1^H/^13^C NMR, HPLC, GC-MS and SPF evaluation	Protection was stronger in solution than in the emulsion, showing that formulation context can reduce the incremental CD effect	[169]
2′-Ethylhexyl-4-(N,N-dimethylamino)benzoate (EHDAB)—PABA-derived UVB filter	Methyl-β-CD and HP-β-CD; 2:1 host:guest cage complexes; K = 1.06 × 10^7^ and 5.67 × 10^6^ L^2^ mol^−2^	250 W high-pressure mercury lamp, 18 cm, up to 8 h; fluorimetric quantification	Degradation: 75.6% free vs. ~1.6% with either CD; photoinduced TICT was also suppressed	UV-Vis, fluorescence, ^1^H NMR, FTIR, TG/DTA; hydrolytic, oxidative and thermal-stability studies	Cage-type complexation strongly protected the filter while suppressing a photoinduced TICT pathway	[170]
Isoamyl 4-(dimethylamino)benzoate (IDB)—PABA-derived UVB filter	SBE-β-CD; IDB:CD = 1:2; Kₐ = 831.85 (mol L^−1^)^−2^; freeze-dried complex	300 W UVB, 10 cm, 8 h, 50% ethanol/water; UV-Vis at 309 nm	Photolysis decreased from 9.16% free IDB to 2.25% for the complex	Job plot, XRD, TGA/DSC, SEM, FTIR, ^1^H NMR and 2D ROESY	A structurally supported 1:2 complex reduced photolysis despite the relatively small absolute loss of the free filter.	[171]
Camphor- and salicylate-based UVB filters						
4-Methylbenzylidene camphor (4-MBC)—organic UVB filter	RM-β-CD (HP-α/β/γ-CDs screened); 1:1 co-evaporated complex incorporated into O/W cream	Xenon solar simulator, λ > 290 nm, 750 W m^−2^, 1 h; HPLC	Degradation decreased from 21.1% free 4-MBC to 7.1% with RM-β-CD; superficial stratum-corneum retention was unchanged	Phase solubility, ^1^H NMR, PXRD, UV-Vis, GC-MS and in vivo tape stripping; BHA/EDTA in both creams	RM-β-CD improved filter photostability without increasing superficial skin retention	[172]
Homosalate—organic UVB filter	2-HP-β-CD; homosalate:CD = 1:2; K ≈ 1040 M^−2^; freeze-dried complex	UVB irradiation, 120 min, 50% ethanol/water; UV-Vis	Photolysis decreased from 21.4% free homosalate to 7.9% for the complex	UV-Vis, Benesi-Hildebrand analysis, TG/DSC, SEM, ^1^H NMR and solubility studies	2-HP-β-CD provided clear photoprotection in a hydroalcoholic medium	[173]
Multicomponent sunscreen filter systems						
Oxybenzone + octocrylene + octinoxate—UVA/UVB filters	HP-β-CD; 1:1 and 2:1 preparation ratios; freeze-dried model complexes and SPF 30 O/W lotion with 0–10% HP-β-CD	Mercury UV lamp, 3 h; UV-Vis + HPLC-UV	Oxybenzone retention 66.3% → 97.2–99.1%; octocrylene retention ~6–8× higher; octinoxate photoisomerization decreased	UV-Vis, DSC, SEM and ^13^C NMR; multicomponent SPF 30 lotion	HP-β-CD improved several distinct photostability endpoints simultaneously in a multicomponent sunscreen formulation	[174]
Oxybenzone + octocrylene + octinoxate—UVA/UVB filters	β-CD; 1:1 structural models; 1:1 and 2:1 preparation ratios; model complexes and SPF 30 O/W lotion	Mercury UV lamp, up to 3 h; UV-Vis	β-CD reduced UV-induced spectral changes in a concentration-dependent manner; individual filter recovery was not quantified by HPLC	UV-Vis, DSC, ^13^C NMR and molecular-mechanics modeling	The study supports a concentration-dependent formulation effect, but the photostability evidence is less chemically specific than HPLC-based comparisons	[175]
Cinnamoyl-CD UV-filter conjugates						
Methoxycinnamoyl-CD monoesters—covalently attached UV-filter chromophores	α-, β- and γ-CD monoesters; covalent C6 conjugates with possible intra-/intermolecular self-inclusion	Broadband UVB in 10% DMSO/water; relative UV absorbance monitored in triplicate	Cavity-matched α-, β- and γ-CD conjugates were more photostable; mismatched β-CD conjugates were not	^1^H/^13^C NMR, ROESY, UV-Vis, DSC, AM1 calculations and cytotoxicity	Photostability followed chromophore-cavity size matching, making geometry more important than CD size alone; this is a covalent self-inclusion model	[53]

Avobenzone provides one of the most extensive bodies of evidence. HP-β-CD reduced its degradation under simulated sunlight both in solution and in an oil-in-water lotion, whereas the corresponding physical mixture provided little protection [166]. The smaller effect observed in the emulsion also showed that the surrounding formulation can reduce the benefit of complexation. This formulation dependence was addressed directly in a subsequent filter-in-CD-in-lipid system. Immediately after preparation, the HP-β-CD complex, BMDBM-loaded lipospheres and lipospheres containing the HP-β-CD/BMDBM complex all reduced photodegradation to a similar extent. During three months of storage, however, the single-carrier systems progressively lost efficacy, whereas the combined CD/lipid formulation maintained a low BMDBM loss of 13.4%, compared with 22.1–23.2% for the single-carrier formulations [167]. The combined carrier therefore provided more persistent protection during storage. In a later study, increasing HP-β-CD markedly improved avobenzone retention while preserving its characteristic UVA absorption; at the highest concentration tested, approximately 79% of the filter remained after irradiation compared with about 17% without CD [25]. Skin permeation, however, did not decrease continuously with HP-β-CD concentration: it increased up to 20% HP-β-CD and then fell at 30%, consistent with a shift toward a surface reservoir of complexed avobenzone. The 30% formulation also reduced UVA-induced edema, whereas differences in sunburn-cell formation among the avobenzone formulations were not statistically significant. These results show that improved chemical photostability does not necessarily produce parallel changes in skin distribution or biological photoprotection.

Protection has subsequently been demonstrated in different physical forms. Freeze-dried 2-HP-β-CD complexes reduced avobenzone photolysis from approximately 49% to 8% under the tested hydroalcoholic conditions [168], while well-characterized crystalline β-CD inclusion compounds of avobenzone and octinoxate also showed strong protection after dissolution; the characteristic photoproducts detected for unprotected filters were not observed after 24 h of UV exposure by ESI-MS/MS [10]. These studies show that photoprotection can be achieved in different physical forms, although its magnitude remains dependent on the formulation.

**Figure 17:**
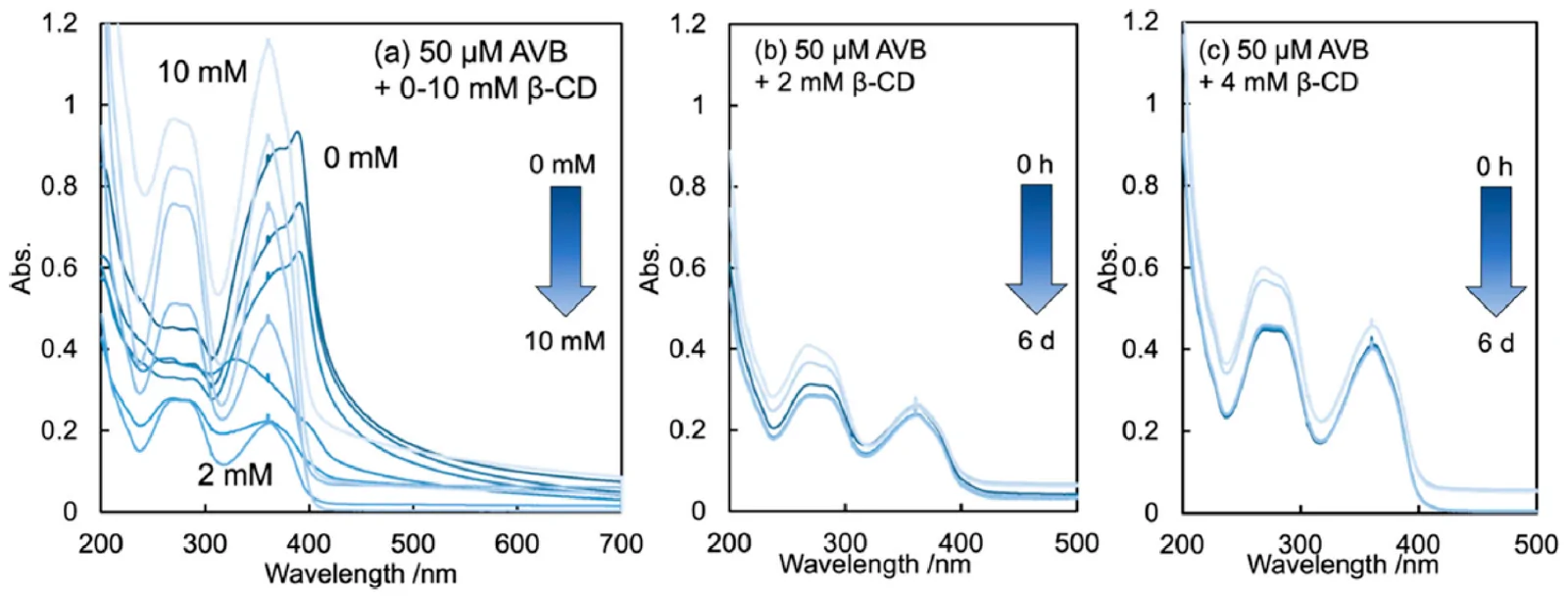
Concentration-dependent effects of β-CD on avobenzone. (**a**) UV-Vis spectra of avobenzone recorded with increasing β-CD concentrations, showing substantial spectral changes at higher host levels. (**b**,**c**) Spectral evolution of avobenzone during prolonged UVA1 irradiation in the presence of 2 or 4 mM β-CD. The spectra illustrate concentration-dependent changes in avobenzone absorption associated with keto-enol/diketo tautomerism and possible aggregation of CD-containing species. Spectral hyperchromicity at higher β-CD concentrations does not independently establish increased chemical degradation. Reproduced from Kuroda et al. (2025) [60] under CC BY 4.0.

More recent work demonstrates that the relationship between cyclodextrin concentration and apparent photoprotection can be non-monotonic. In the avobenzone/β-CD system studied by Kuroda et al., the most favorable UVA1 photostability was reported at intermediate AVB:β-CD molar ratios of approximately 1:50–1:100 [60]. This behavior was associated with changes in the local molecular environment and the equilibrium between the chelated keto-enol and diketo tautomers of avobenzone. The chelated keto-enol form is responsible for strong UVA absorption, whereas the diketo form absorbs predominantly at shorter wavelengths and was comparatively insensitive to the UVA1 irradiation employed in that study. The authors proposed that encapsulation within the β-CD cavity favors the diketo population and consequently reduces UVA1-induced degradation. However, the diketo form should not be regarded as universally photostable, since it may undergo photochemical reactions upon excitation at shorter wavelengths. At elevated β-CD concentrations, pronounced hyperchromic effects and deviations from the initial phase-solubility behavior were attributed to the formation of aggregated AVB/β-CD-containing particles (Figure 17). Such aggregation may modify guest distribution, local solvation and optical properties, but its specific contribution to the observed photochemical response was not independently quantified. Changes in water activity might also influence the supramolecular environment, although this mechanism was not directly investigated. Importantly, the increased absorption observed at higher β-CD concentrations should not automatically be interpreted as accelerated chemical degradation. Overall, this system illustrates how concentration-dependent inclusion, tautomeric redistribution and aggregation-related effects may jointly influence the measured photochemical behavior, without establishing a single dominant mechanism.

The effect is not limited to avobenzone. HP-β-CD reduced degradation of the PABA-derived filter 2-ethylhexyl *p*-dimethylaminobenzoate in both solution and emulsion [169], with substantially stronger protection in solution than in the oil-in-water formulation. GC–MS analysis further showed that complexation reduced, but did not completely suppress, photoproduct formation. A much stronger effect was observed for the related 2′-ethylhexyl-4-(N,N-dimethylamino)benzoate: after 8 h of irradiation, photodegradation reached 75.6% for free EHDAB but only about 1.6% in the presence of either methyl-β-CD or HP-β-CD [170]. Fluorescence measurements showed simultaneous suppression of the photoinduced twisted intramolecular charge-transfer state, indicating that complexation also altered the photophysical behavior of the filter. A structurally characterized 1:2 SBE-β-CD complex of isoamyl 4-(dimethylamino)benzoate likewise reduced photolysis from 9.16% to 2.25%, although the absolute degradation of the free filter was comparatively small [171].

Host identity can be equally important. For 4-methylbenzylidene camphor, complexation with RM-β-CD reduced degradation from 21.1% to 7.1%, whereas the corresponding HP-β-CD system did not provide significant protection [172]. RM-β-CD complexation did not significantly alter the amount of filter retained in the superficial layers of the human stratum corneum, indicating that improved photostability was not accompanied by a clear change in superficial skin retention. Homosalate provides a simpler example in which a 1:2 complex with 2-HP-β-CD reduced photolysis from 21.4% to 7.9% in a hydroalcoholic medium [173].

In multicomponent formulations containing oxybenzone, octocrylene and octinoxate, HP-β-CD increased filter retention and reduced light-induced isomerization [174]. This study compared several aspects of filter photostability within the same SPF 30 lotion: oxybenzone retention increased from 66.3% to 97.2–99.1%, octocrylene recovery increased several-fold, and octinoxate photoisomerization was reduced. This suggests that one CD concentration may not be optimal for every component of a multicomponent sunscreen. A later study with native β-CD supported concentration-dependent spectral stabilization of the same filter combination, although the evidence was less chemically specific because individual filter recovery was not quantified by HPLC [175]. For sunscreen systems, photostability may therefore involve not only preservation of total filter content but also maintenance of the molecular form responsible for the desired spectral performance. Covalent CD-chromophore systems provide a complementary perspective. Methoxycinnamoyl-CD monoesters showed improved photostability only when chromophore dimensions were appropriately matched to the α-, β- or γ-CD cavity; mismatched β-CD conjugates did not benefit from covalent attachment [53]. As discussed in Section 2, this example shows that covalent attachment alone does not guarantee photoprotection.

The magnitude of protection nevertheless depends strongly on the supramolecular and formulation environment. Crosslinked CD polymers likewise produced protective, negligible or adverse effects depending on filter identity, solvent, crosslinker chemistry and oxygen availability [61,176]. These formulation-level results reinforce the same point seen in simpler inclusion complexes: shielding, guest mobility, oxygen access and the physical organization of the carrier can act in parallel and may change the direction of the observed effect. These systems are discussed more broadly as advanced CD architectures in Section 5.

Importantly, favorable CD interactions do not necessarily improve chemical photostability. SBE-β-CD reduced penetration of oxybenzone into deeper skin layers but produced little change in its degradation under simulated sunlight [177]. Improved topical localization or formulation performance should therefore not be interpreted automatically as intrinsic photoprotection. Absent, adverse and strongly condition-dependent effects are considered separately in Supplementary Table S2.

The sunscreen studies summarized in Table 3 illustrate the interplay between inclusion geometry, conformational restriction and excited-state processes. The dependence of methoxycinnamoyl-CD photoprotection on chromophore–cavity matching [53] exemplifies the geometric considerations discussed in Section 2.1, whereas suppression of a photoinduced charge-transfer state in the EHDAB complexes [170] is consistent with conformational and excited-state modulation (Section 2.2 and Section 2.4). Changes in the local environment, including oxygen availability and formulation composition (Section 2.3), may further influence the effects observed in crosslinked CD polymers [61,176]. Conversely, the non-monotonic dependence of avobenzone photostability on β-CD concentration [60] and the absence of a significant chemical photostability benefit for oxybenzone [177] illustrate the limitations discussed in Section 2.5. These findings demonstrate that no single mechanism can explain the behavior of all sunscreen filters. Successful stabilization must therefore be evaluated through preservation of the parent compound, control of photoisomerization and maintenance of the intended UV-absorption properties within the final formulation.

### 4.4. Dyes, Molecular Probes and Photosensitizers

Dyes, molecular probes and photosensitizers differ from most other compounds considered in this review because light absorption is integral to their intended function. Fluorescent probes must tolerate repeated excitation without rapid photobleaching, whereas photosensitizers must remain stable enough to retain useful photochemical activity. CD-mediated photoprotection therefore requires selectivity: destructive pathways should be restricted without compromising fluorescence, sensing performance or photodynamic activity. Relevant studies are summarized in Table 4.

**Table 4:** Photostability of dyes, molecular probes and photosensitizers in cyclodextrin-containing systems.

Guest/Application	Cyclodextrin Systems	Irradiation/Assessment	Photostability Outcome	Supporting Evidence	Interpretation	Ref.
Fluorescent probes and laser dyes						
Anthracene chromophore (β-CD-insulated rotaxane)—fluorescent acene for optoelectronic materials	β-CD; 1:1 mechanically interlocked rotaxane; β-CD-insulated anthracene with uninsulated dumbbell control	380 nm, pH 11.4 phosphate buffer, ~7 μM; UV-Vis photobleaching kinetics	T_1/2_ increased from 13 ± 0.4 to 109 ± 4 min; β-CD insulation also inhibited anthracene photodimerization	ESI-MS, ^1^H NMR, 2D NOESY, UV-Vis and fluorescence	Persistent molecular insulation suppresses intermolecular photodimerization and markedly extends photostability	[54]
Coumarin 1, coumarin 4 and coumarin 120—laser dyes	β-CD; 1:1 spectroscopic inclusion systems in aqueous ethanol; CD concentration varied in laser experiments	Lamp-pumped dye laser, ~400 J, ~3 μs pulses; generation energy monitored over repeated pulses	CD complexes showed higher generation energy than free dyes; after five pulses (~2 kJ), output remained near the initial output of free dyes	UV-Vis, fluorescence, FTIR, Raman and quantum-chemical calculations	Complexation improved operational laser-dye performance, although the endpoint reflects optical durability rather than direct parent quantification	[178]
Coumarin 7—fluorescent probe for sensing and bioimaging	SBE7βCD (DS ≈ 6.4; β-CD comparator); 1:1, K = 2.3 × 10^4^ M^−1^ at pH 3 and 8.1 × 10^3^ M^−1^ at pH 7; aqueous assemblies	Ambient daylight at pH 7.4; absorbance loss over time	SBE7βCD slowed daylight-induced absorbance loss; no numerical photobleaching endpoint; fluorescence lifetime increased from 1.08 to 2.45 ns at pH 7	Job plot, UV-Vis, steady/time-resolved fluorescence, anisotropy, ^1^H NMR, circular dichroism and Drosophila imaging; competitive displacers	The CD microenvironment improved optical persistence and altered excited-state behavior, but quantitative photostability evidence is limited	[179]
Coumarin 30—fluorescent probe for bioimaging	SBE7βCD and SBE7βCD-functionalized AuNPs; 1:1 C30:SBE7βCD; K ≈ 1.24 × 10^4^ M^−1^ free and 1.3 × 10^9^ M^−1^ on AuNPs	Continuous irradiation at pH 7; normalized optical-density decay (irradiation details in Supporting Information)	Free C30 photobleached faster than both CD systems; AuNP–SBE7βCD:C30 showed the slowest optical-density loss	UV-Vis, fluorescence, TCSPC, anisotropy, Job plot, ITC, FTIR, DLS, ζ-potential, TEM/HRTEM and SAED	Surface-immobilized CD produced the strongest apparent protection, highlighting an architecture-dependent effect beyond simple inclusion	[180]
Fluorescein, tetrafluorofluorescein and tetrachlorofluorescein—fluorescent dyes	β-CD; 1:1 binding model; K = 122 ± 15 M^−1^ (tetrafluoro) and 637 ± 70 M^−1^ (tetrachloro); aqueous pH 8 buffer	250 W mercury arc lamp; UV-Vis photobleaching	β-CD slowed photobleaching of all three dyes, with the largest effect for tetrachlorofluorescein; CB [7] gave no protection	^1^H/^13^C/^19^F NMR, UV-Vis, binding constants and relative photobleaching quantum yields; CB [7] comparator	Protection was structure-dependent and host-specific, indicating that inclusion geometry/interaction rather than generic confinement controls the outcome	[181]
IAD1–IAD4 dyes (mainly IAD4)—fluorescent probes for STED imaging	β-CD; 1:1 for IAD1/IAD4 and 2:1 for IAD2/IAD3; IAD@β-CD nanoassemblies (~4–6 nm)	Continuous 470 nm excitation, 0.1 kW cm^−2^, >600 s; fluorescence decay; HeLa confocal/STED imaging	After 600 s, IAD4 retained ~6% fluorescence vs. 78% for IAD4@β-CD; in HeLa cells the complex retained ~67%	^1^H NMR, FTIR, SCXRD, TEM, DLS, UV-Vis, fluorescence and stimulated-emission measurements	CD confinement strongly suppresses excitation-driven signal loss by restricting molecular motion and intermolecular excited-state interactions	[12]
Pacific Blue—fluorescent probe for bioimaging	6-Amino-6-deoxy-β-CD; covalent 1:1 self-included conjugate	390 ± 10 nm, 300 W Xe lamp, up to 90 min; UV-Vis/fluorescence kinetics; live-cell CLSM	k decreased from 2.0 × 10^−2^ to 7.3 × 10^−3^ min^−1^ (2.8-fold); cellular fluorescence loss was 9.5 vs. 1.0% per scan for control vs. PB-CD	NOESY NMR, UV-Vis, fluorescence, ROS assays and confocal microscopy	Intramolecular self-inclusion improves both solution photostability and optical durability in living cells	[32]
Paramagnetic molecular probes						
Indolinonic nitroxide radical—paramagnetic spin probe and antioxidant	RM-β-CD (HP-α/β/γ-CDs screened); 1:1, K_1:1_ = 11,431 ± 879 M^−1^; freeze-dried complex and 30% acetonitrile/water solution	Suntest CPS^+^ xenon, λ > 290 nm, 250 W m^−2^, 2 h; solid and solution; residual nitroxide by HPLC	Solid-state degradation: 78.8 ± 5.1% free vs. 28.0 ± 6.4% complex; solution: 69.1 ± 7.0% vs. 52.7 ± 4.0%, falling to 41.2 ± 4.4% with excess RM-β-CD	Phase solubility, PXRD, DTA and EPR; antioxidant assays in methyl-linoleate and BSA systems	Protection was substantially stronger in the solid state, demonstrating the importance of physical state and bound fraction	[182]
Cyanine, hemicyanine and indocyanine dyes						
Cyanine dye rotaxanes 3a/3b—fluorescent dyes	α-CD; one α-CD per dye; mechanically interlocked cyanine rotaxanes	O_2_ -saturated water and dioxane; visible white light; absorbance and pseudo-first-order photobleaching kinetics	Rotaxane 3b was ~40-fold more photostable in water and ~2-fold more photostable in dioxane than the free dye	^1^H/^13^C NMR, NOESY, mass spectrometry, UV-Vis and fluorescence	Permanent CD insulation produces large protection in water, while the smaller dioxane effect shows strong solvent dependence	[48]
Cyanine dyes 1a–1d/β-CD conjugates 2a–2d—fluorescent labels	6-Amino-6-deoxy-β-CD; covalent 1:1 dye-CD conjugates with proposed intramolecular self-inclusion	Continuous photobleaching in water and Swiss 3T3 cells; pseudo-first-order fluorescence decay	Relative photostability: 9.1 (2a) and 20 (2d) vs. 1.0 for Cy5; in cells, ~7- and ~8-fold greater stability than dextran-linked dye	^1^H NMR, fluorescence quantum yield, photobleaching kinetics, CPK modeling and live-cell imaging; Cy5/dextran controls	Linker-dependent self-inclusion markedly increases aqueous and cellular photostability of cyanine fluorophores	[183]
Hemicyanine dye-β-CD conjugate 3a—fluorescent dye	β-CD; covalent 1:1 conjugate with proposed intramolecular self-inclusion	Natural/ambient light, 25 °C, 4 days; UV-Vis absorbance at λmax	Relative photostability was 10.2-fold higher than for the corresponding non-CD hemicyanine dye	^1^H NMR, IR, UV–Vis, fluorescence and elemental analysis	Covalent attachment and self-inclusion provide persistent chromophore protection under ambient-light exposure	[184]
HITC cyanine dye—photographic/optical-recording dye	β-CD; approximate 2 HITC:1 β-CD dimer-inclusion model; in situ aqueous system	365 nm mercury lamp; absorbance, photodegradation quantum yield and ^1^O_2_ emission at 1268/1580 nm	At β-CD:HITC = 600:1, photodegradation quantum yield fell to ~35% of the free-dye value; ^1^O_2_ formation and attack on HITC also decreased	UV-Vis, ^1^H NMR, flash photolysis, singlet-oxygen lifetime and MNDO/PM3 calculations	Direct ^1^O_2_ measurements show that β-CD can reduce both ROS generation and subsequent oxidative attack on the guest	[44]
Indocyanine chromophore TK-1 (ICG comparator)—NIR imaging contrast agent	Two covalently attached 2A-O-(3-aminopropyl)-β-CD units; dye:(β-CD)_2_ conjugate	0.1 μM in PBS, pH 7.4, 25 °C; continuous 760 nm irradiation for 200 min; fluorescence at 810 nm	TK-1 fluorescence decreased more slowly than ICG over 200 min irradiation	^1^H/^13^C NMR, HPLC-PDA-MS, UV-Vis-NIR, fluorescence quantum yield, blood aggregation and in vivo NIR imaging	The di-β-CD conjugate improves NIR fluorescence persistence, although comparison is to ICG rather than a matched CD-free TK-1 analogue	[185]
Indocyanine green (ICG)—NIR photothermal/photodynamic agent	Methyl-β-CD; optimized ratio not reported; ICG/Me-β-CD complex subsequently loaded into visible-light-cured CMCSMA hydrogel	Repeated 808 nm NIR irradiation; reheating and color-change comparison of free ICG vs. ICG/Me-β-CD	Free ICG showed markedly lower reheating in the second cycle and greater color change; the Me-β-CD complex maintained higher reheating performance	UV-Vis,^1^H NMR, FTIR, thermal imaging, rheology, release, ROS, cell/in vivo assays; CMCSMA hydrogel + riboflavin photoinitiator	CD complexation improved repeated NIR performance, but the final hydrogel system is a multicomponent architecture	[186]
Porphyrin-based photosensitizers and PDT systems						
BPDMA and all-trans-retinoic acid (atRA)—photosensitizer/light-sensitive retinoid	PMDA-crosslinked β-CD nanosponge (βNS-PYRO); BPDMA-loaded aqueous nanosponge and atRA nanosponge/Carbopol gel	BPDMA: 690 nm, 150 W Xe, up to 6 h, absorbance kinetics; atRA: sunlight up to 3 h, HPLC	BPDMA kPB decreased from 17.81 × 10^−5^ to 4.03 × 10^−5^ s^−1^; atRA t_1/2_ increased ~200-fold vs. ethanol and 8-fold vs. Carbopol gel	DLS, SEM, rheology, UV-Vis, fluorescence, HPLC and singlet-oxygen assay; Carbopol comparator	Crosslinked β-CD nanosponges can strongly improve photostability, but the magnitude depends on the reference matrix and complete carrier architecture	[30]
Cationic porphyrin—photosensitizer for photodynamic cancer therapy	Heptakis(2,3-di-O-methyl)-β-CD and octakis(2,3-di-O-methyl)-γ-CD; porphyrin:CD = 1:2; mechanically interlocked [5]rotaxanes	405 nm-filtered 250 W mercury lamp, 120 min; UV-Vis; PDT under 405 nm LED	β-3 and γ-3 showed only minor absorbance loss, whereas the CD-free analogue rapidly photobleached; γ-3 slightly more stable than β-3	^1^H/^13^C NMR, NOESY, HPLC, fluorescence, ^1^O_2_ quantum yield, transient absorption, modeling and cell assays; CB [6] component	Defined interlocked CD shells provide durable chromophore insulation while retaining photosensitizing function	[33]
Hematoporphyrin (HP) + ICG—controllable PDT/NIR photothermal switch	β-CD-grafted G5 PAMAM dendrimer; HP alone (CPH) or HP/ICG (CPHI); no discrete molecular stoichiometry	Sunlight 15 d; HP by UV-Vis at 399 nm; additional 808 nm activation and 660 nm PDT	After 15 days, HP retention: 4.0% free, 23.3% CPH and 89.2% CPHI10; 808 nm irradiation degraded ICG and restored HP photoactivity	^1^H NMR, UV-Vis, fluorescence/FRET, DLS, ζ-potential, loading/release, ROS and cell assays	The strongest stabilization required ICG, so the effect reflects a switchable multicomponent architecture rather than CD alone	[187]
TMPyP—porphyrin photosensitizer for antimicrobial/anticancer PDT	Captisol (SBE7βCD); Captisol:TMPyP = 1:2; K_1_ ≈ 3.0 × 10^4^ and K_2_ ≈ 3.1 × 10^4^ M^−1^; self-assembled nanorods	422 ± 2.5 nm, 150 W Xe lamp, ~80 μW cm^−2^, 1 h; Soret-band photobleaching	Captisol slowed Soret-band photobleaching and increased ^1^O_2_ quantum yield from 0.73 to 0.95	UV-Vis, steady/time-resolved fluorescence, anisotropy, ^1^H NMR, ITC, DLS, SEM/AFM and DPBF/MTT assays	CD association simultaneously reduced bleaching and preserved/enhanced productive singlet-oxygen generation	[188]
TMPyP—cationic photosensitizer for antimicrobial PDT	CAPTISOL^®^ (SBE-β-CD, DS ≈ 6.5); 1:1, K_b_ ≈ 1.32 × 10^5^ M^−1^; freeze-dried redispersible nanoassemblies (~360 nm)	PBS or Mueller-Hinton broth, 37 °C for 4 days, then white LED 2.92 mW cm^−2^ for 4 h; hourly UV-Vis	After 4 h, 97.5%/96.5% remained in PBS/broth vs. 95%/92% free TMPyP	UV-Vis, steady/time-resolved fluorescence, DLS, ζ-potential, SNOM, release, ^1^O_2_ quantum yield and antimicrobial assays	Only modest absolute photostability gains were observed, while antimicrobial photodynamic activity was retained	[189]
Other photosensitizers						
Curcumin—natural polyphenol/antimicrobial PDT photosensitizer	HP-β-CD and HP-γ-CD; HP-γ-CD used for photostability; competitive Cur–HSA/CD binding in PBS	Fluorescent light 350–500 nm (λmax 430 nm), 18 mW cm^−2^, 5–15 min; HPLC	HSA-bound Cur fell to 6.3% after 5 min and was undetectable after 10 min; HP-γ-CD produced concentration-dependent protection in HSA mixtures	HSA fluorescence quenching, Stern-Volmer, UV-Vis, fluorescence and Stokes-shift analysis; PEG 400/Pluronic comparators	Protection depended strongly on host concentration and protein matrix, illustrating competitive binding and environment-dependent photochemistry	[190]
Parietin (physcion)—anthraquinone photosensitizer for antimicrobial PDT	HP-β-CD; 1:1, K_s_ ≈ 733 M^−1^; freeze-dried inclusion complex	Blue LED 457 nm, 220.2 W m^−2^, up to 30 min; UV-Vis photobleaching + ^1^O_2_ generation	After 30 min, ~70% complexed PTN remained vs. <28% free; ΦΔ decreased from 0.49 to 0.35, but aPDT activity was retained	Phase solubility, Job plot, ^1^H NMR/ROESY, ATR-FTIR, PXRD, SEM, DSC and CFU assay	Photoprotection was achieved without loss of antimicrobial PDT efficacy, despite a moderate reduction in singlet-oxygen yield	[191]
Industrial and textile dyes						
Synthetic indigo and Jeju indigo—vat dyes for cotton/denim	β-CD and HP-β-CD; 1:1, A_l_-type phase solubility; coprecipitated/kneaded complexes in aqueous systems and dyed cotton	Ambient light/dark storage 30 days; UVC 254 nm 7 days; fabric sunlight exposure 7 days; color/fading endpoints	For synthetic indigo fabric, Δ_E_*ab after sunlight decreased from 11.41 control to 3.91 (β-CD) and 3.73 (HP-β-CD); Jeju indigo/HP-β-CD gave 9.15	UV-Vis, FTIR, PXRD, TEM,^1^H NMR, thermal stability, CIELAB, washing fastness and fixation efficiency	CD complexation improved textile color fastness, with performance dependent on dye source and host identity	[192]

Early studies with cyanine and related chromophores showed that covalent or mechanically interlocked association with a CD can markedly increase resistance to photobleaching. Covalent β-CD–cyanine conjugates were more photostable than corresponding non-CD dyes in solution and in cells [183], with the magnitude of protection depending strongly on linker length. An α-CD cyanine rotaxane displayed an approximately 40-fold photostability advantage over the free dye in water but only an approximately twofold advantage in dioxane, showing that the magnitude of protection remained strongly solvent-dependent [48]. β-CD insulation of an anthracene chromophore likewise increased the photobleaching half-life from approximately 13 to 109 min and suppressed photodimerization [54]. Together, these studies showed that persistent CD association can markedly extend chromophore photostability, although the magnitude of protection remains system-dependent. Covalent and mechanically interlocked CD architectures are considered more broadly in Section 5.

Reversible inclusion can also improve dye stability, but the magnitude of the effect depends strongly on chromophore structure. β-CD slowed photobleaching of several fluorescein derivatives, with the strongest effect observed for tetrachlorofluorescein [181]. For coumarin laser dyes, β-CD inclusion also improved output over repeated high-energy pulses, although the measured response was operational laser performance rather than direct quantification of the intact dye [178]. Thus, improved optical performance does not necessarily provide direct evidence of chemical preservation.

The practical advantage is especially clear in fluorescence microscopy, where photobleaching directly limits observation time. β-CD nanoassemblies of IAD dyes markedly prolonged fluorescence under the high excitation intensities used for STED imaging; for IAD4, approximately 78% of the initial fluorescence remained after 600 s compared with about 6% for the free dye, with a substantial benefit also observed in living cells [12]. Pacific Blue linked to β-CD through a self-including architecture likewise showed a 2.8-fold decrease in the apparent photodegradation rate constant in aqueous solution and markedly greater signal persistence in living cells: fluorescence loss during repeated confocal scanning was approximately 1.0% per scan for PB-CD compared with 9.5% per scan for the PB-NHS control [32]. A related trend was observed for Coumarin 30, for which soluble SBE7βCD reduced photobleaching and surface immobilization of SBE7βCD on gold nanoparticles provided still greater optical persistence [180]. Because the latter system adds a nanoparticle contribution to CD association, its enhanced performance cannot be assigned to inclusion alone. In such applications, the relevant outcome is not only preservation of chromophore integrity but preservation of a usable optical signal during repeated excitation.

Direct chemical measurements provide a useful complement to fluorescence-based observations. The indolinonic nitroxide radical studied by Damiani and co-workers provides an example in which the remaining guest was quantified directly by HPLC. RM-β-CD reduced degradation much more strongly in the solid state, from 78.8 ± 5.1% for the free nitroxide to 28.0 ± 6.4% for the complex, than in solution, where degradation decreased from 69.1 ± 7.0% to 52.7 ± 4.0%; excess RM-β-CD further reduced the loss to 41.2 ± 4.4% [182]. The difference emphasizes the importance of physical state and the fraction of guest that remains associated with the host during irradiation.

Photosensitizers present a different challenge because chemical photostability and photodynamic functionality are distinct properties that do not necessarily change in parallel. Reduced photobleaching is desirable only when sufficient ROS-generating capacity and biological activity are maintained. A β-CD nanosponge containing BPDMA, for example, reduced the photobleaching rate while preserving singlet-oxygen production [30]. However, CD association may also directly influence the efficiency of singlet-oxygen generation, expressed as the quantum yield (ΦΔ), independently of its effect on chromophore degradation. In Captisol/TMPyP supramolecular assemblies, reduced photobleaching was accompanied by an increase in ΦΔ from 0.73 to 0.95 [188]. The authors attributed this enhancement to reduced nonproductive aggregation and changes in excited-state behavior, including a longer excited-state lifetime potentially favoring triplet-state sensitization. Interestingly, the same study reported a substantially lower ΦΔ of 0.47 in the presence of native β-CD, demonstrating that different CD hosts can exert opposite effects on the photochemical functionality of the same photosensitizer.

A contrasting result was reported for another Captisol/TMPyP formulation, in which ΦΔ decreased from 0.74 to 0.58 despite improved chromophore retention and preserved antimicrobial photodynamic activity [189]. Although reduced aggregation was also observed in this system, it did not translate into enhanced singlet-oxygen generation. Deaggregation therefore represents a plausible and experimentally supported mechanism for improving photosensitizer performance in selected systems, as illustrated by the CD-mediated activation of pheophorbide a [13], but cannot be considered a universal explanation for changes in ΦΔ. Other potential contributions include changes in triplet-state formation and deactivation, oxygen accessibility, and the local supramolecular environment. Their relative importance remains insufficiently established in many reported systems, and differences in experimental media and analytical procedures further limit direct quantitative comparisons between studies. These findings highlight both a design opportunity and a potential limitation: CD association may be exploited to enhance productive photochemistry, but increased photostability can also coincide with reduced singlet-oxygen generation. Consequently, photosensitizer formulations should be evaluated using separate measurements of photobleaching, singlet-oxygen generation (preferably quantified through ΦΔ or supported by suitably controlled EPR/ESR measurements) and photodynamic activity, with experimental conditions matched as closely as possible to distinguish optical stability from preservation of productive photochemistry.

HP-β-CD complexation of parietin increased the fraction remaining after 30 min of blue-light exposure from less than 28% to approximately 70%, while antimicrobial PDT activity was retained despite a decrease in ΦΔ from 0.49 to 0.35 [191]. The magnitude of the photostability difference should nevertheless be interpreted cautiously because free parietin and the HP-β-CD complex were irradiated in ethanol and water, respectively. The surrounding biological environment can also influence the observed effect. In HSA-containing curcumin solutions, only 6.3% of the initial curcumin remained after 5 min of irradiation, and none was detectable after 10 min, whereas increasing HP-γ-CD concentrations markedly improved photostability in the Cur-HSA mixtures [190]. The extent of protection therefore depended on both HP-γ-CD concentration and the presence of protein. These examples show that photosensitizer stability should be considered together with ROS generation and photodynamic activity.

**Figure 18:**
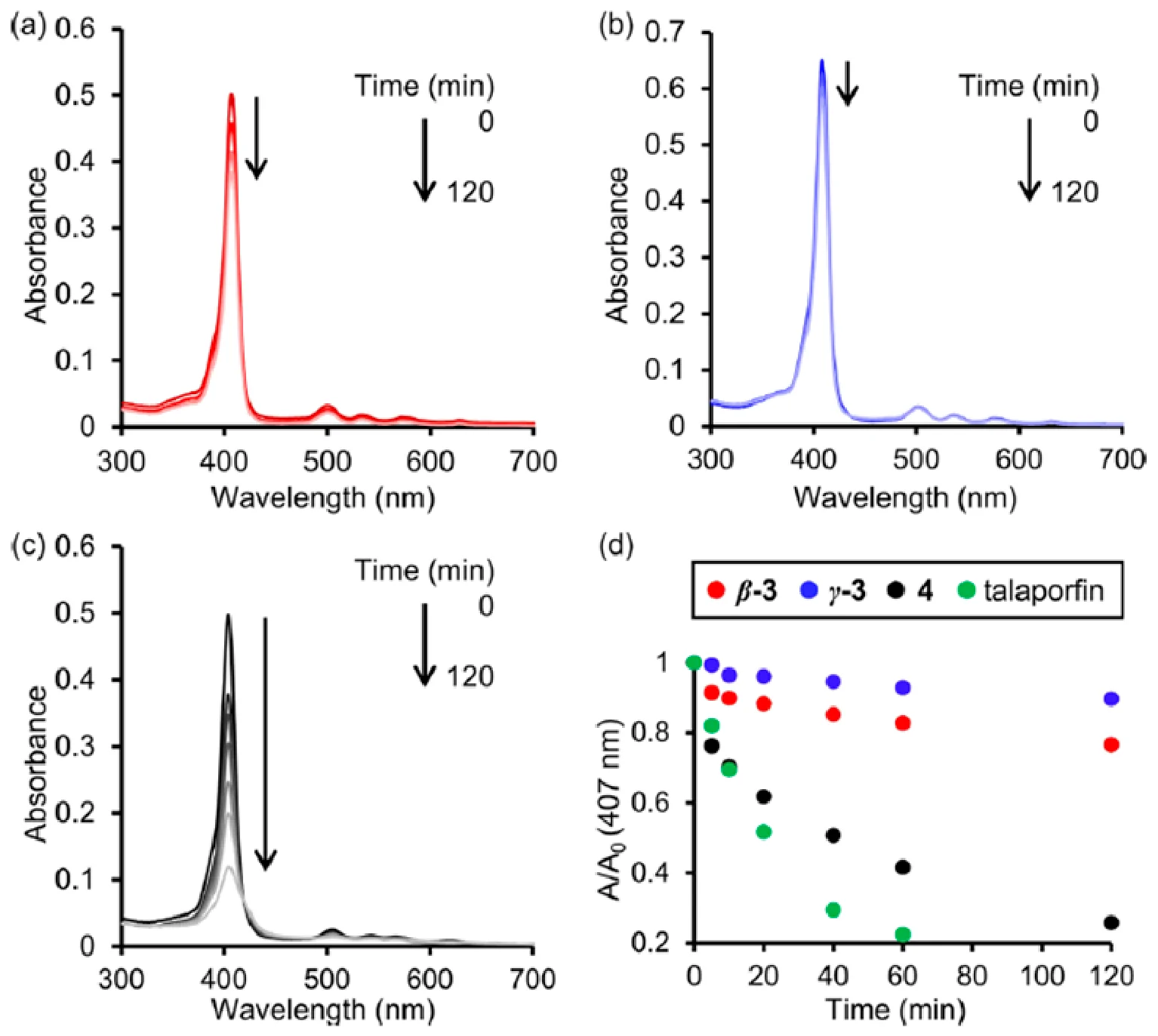
Photostability of cyclodextrin-insulated porphyrin rotaxanes. Absorption spectral changes in the β-CD [5]rotaxane β-3, γ-CD [5]rotaxane γ-3 and the corresponding CD-free [3]rotaxane 4 during continuous irradiation with a 405 nm-filtered high-pressure mercury lamp, together with the time-dependent absorbance at 407 nm and comparison with talaporfin sodium. The CD-insulated rotaxanes showed only minor spectral loss over 120 min, whereas the CD-free analogue was rapidly photobleached. Reproduced from Ohishi et al. (2024) [33] under CC BY 4.0.

Mechanically interlocked porphyrins provide a clear example of this balance. β- and γ-CD-insulated porphyrin rotaxanes resisted photobleaching substantially better than the corresponding CD-free [3]rotaxane while retaining singlet-oxygen generation and light-induced cytotoxicity (Figure 18) [33]. The γ-CD-insulated structure was slightly more resistant to photobleaching than the β-CD analogue, consistent with modeling showing more extensive coverage of the porphyrin core by the larger γ-CD cavity. Importantly, the CD shells did not abolish oxygen sensitization: β- and γ-CD [5]rotaxanes retained high ^1^O_2_-generation efficiencies while the CD-free comparator rapidly photobleached. This system therefore combines improved resistance to photobleaching with retention of the desired photosensitizing function.

More elaborate systems combine CD-mediated protection with polymers, nanoparticles, additional chromophores or photothermal components. In β-CD-grafted PAMAM carriers, for example, hematoporphyrin retention increased further when ICG was incorporated as an additional quencher and NIR-responsive switch [187], while ICG/Me-β-CD systems have been used to improve repeated photothermal performance under 808 nm irradiation [186]. Such systems may perform well, but attribution becomes progressively less specific because the observed response reflects the complete carrier architecture rather than CD association alone. They are therefore considered more fully in Section 5 and Section 6.

**Figure 19:**
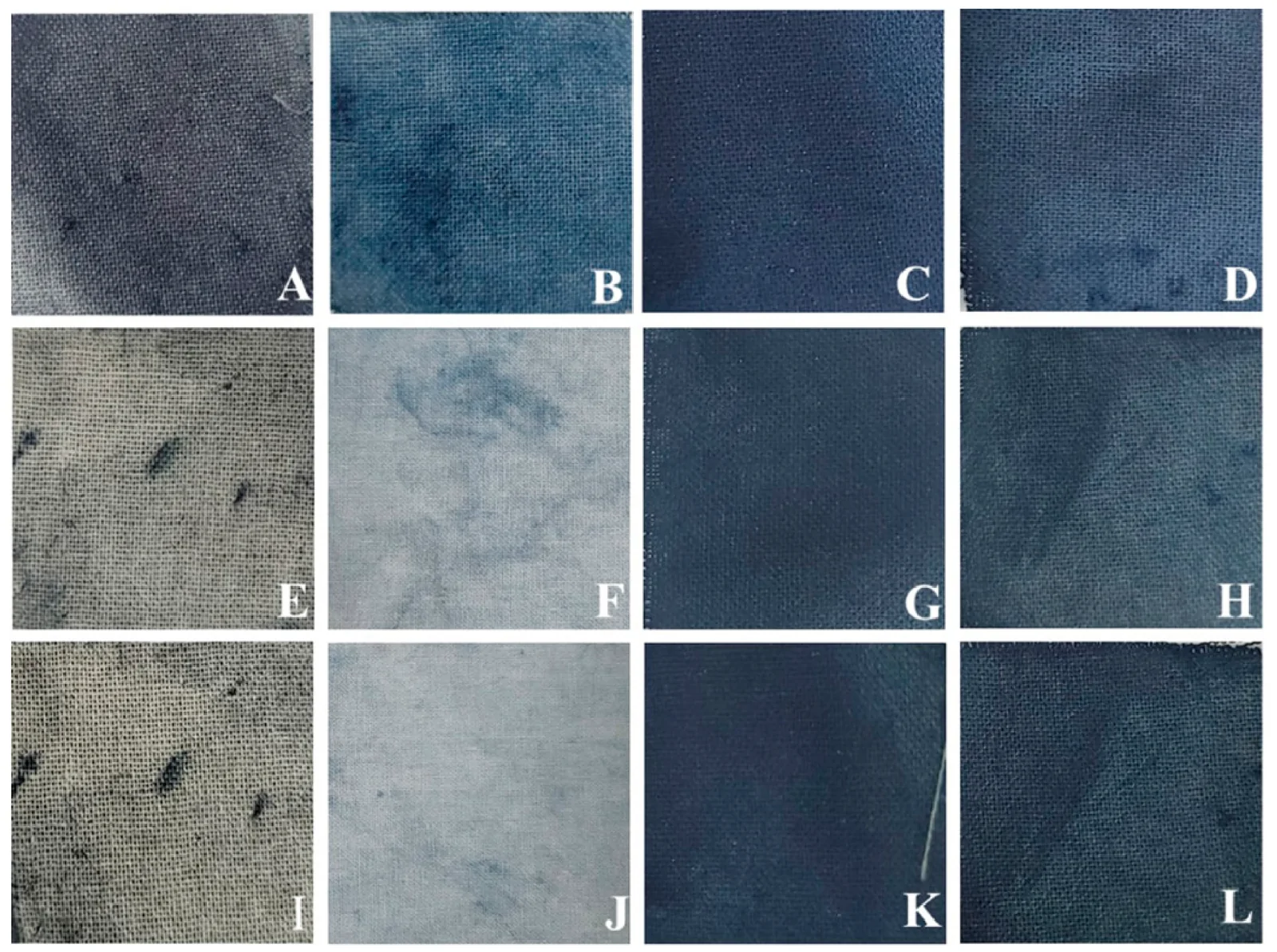
Sunlight stability of cotton fabrics dyed with synthetic or natural indigo in the absence or presence of cyclodextrins. Fabrics were dyed using 5% (*w*/*v*) suspensions prepared by the kneading method and exposed to natural sunlight. Panels are arranged by exposure time: (**A**–**D**) 0 days, (**E**–**H**) 3 days, and (**I**–**L**) 7 days. Within each row, the samples are, from left to right: (**A**,**E**,**I**) synthetic indigo without cyclodextrin (control); (**B**,**F**,**J**) Jeju indigo/HP-β-CD; (**C**,**G**,**K**) synthetic indigo/β-CD; and (**D**,**H**,**L**) synthetic indigo/HP-β-CD. After 7 days, the synthetic indigo/β-CD and synthetic indigo/HP-β-CD fabrics showed markedly lower color change than the CD-free control, consistent with ΔE*ab values of 3.91 and 3.73, respectively, compared with 11.41 for the control; the Jeju indigo/HP-β-CD fabric showed a smaller improvement (ΔE*ab = 9.15). Reproduced from Devi et al. (2026) [192] under CC BY 4.0.

The scope of CD-mediated photoprotection also extends beyond biomedical probes. In cotton dyed with synthetic indigo, the color difference after seven days of natural sunlight exposure decreased from ΔE*ab = 11.41 for the CD-free control to 3.91 and 3.73 for β-CD- and HP-β-CD-containing systems, respectively (Figure 19) [192]. The corresponding Jeju indigo/HP-β-CD system showed a smaller improvement (ΔE*ab = 9.15), indicating that the outcome depended on dye source as well as host identity. Here, color retention is itself a directly relevant measure of performance.

Direct reports of adverse CD effects on photostability are relatively uncommon in this literature. The predominance of favorable results should nevertheless be interpreted cautiously. Much of the literature concerns deliberately engineered dye-CD conjugates, rotaxanes or supramolecular assemblies developed specifically to improve optical performance. In other studies, fluorescence persistence, ROS generation or biological activity is reported without matched quantification of the intact free chromophore. The small number of adverse results therefore cannot be taken as evidence that CD association is intrinsically protective for photoactive dyes.

The systems summarized in Table 4 demonstrate that effective photoprotection of dyes and molecular probes frequently involves spatial isolation of chromophores and restriction of their molecular motion. The cyanine and anthracene rotaxanes [48,54] illustrate the importance of inclusion geometry and intermolecular organization (Section 2.1 and Section 2.2), whereas the HITC system [44] provides direct evidence that CD association can modify singlet-oxygen formation and accessibility (Section 2.3). For photosensitizers, however, modulation of excited-state pathways (Section 2.4) must also be considered in relation to the desired photochemical function. The opposing changes in singlet-oxygen quantum yield reported for Captisol/TMPyP systems [188,189] demonstrate that improved resistance to photobleaching does not necessarily correspond to enhanced ROS generation. These observations also reinforce the limitations outlined in Section 2.5: reduced photobleaching alone is insufficient to establish an overall beneficial effect. Consequently, mechanistic interpretation should consider chemical stability together with fluorescence performance, ROS generation or photodynamic activity, depending on the intended application.

### 4.5. Agrochemicals

Agrochemicals form a relatively small but important group of CD–guest systems. Unlike pharmaceuticals or food bioactives, which may enter the environment through various pathways, agrochemicals are generally applied intentionally to agricultural environments, where their photochemical fate is influenced by natural organic matter, dissolved constituents, oxygen, pH and light penetration. CD-mediated stabilization may therefore prolong the useful lifetime of an active ingredient, but excessive protection may also increase environmental persistence. The surrounding water matrix can therefore strongly influence both the magnitude and direction of the CD effect, making environmentally relevant conditions particularly important. The available studies are summarized in Table 5.

**Table 5:** Photostability of agrochemicals in cyclodextrin-containing systems.

Guest/Application	Cyclodextrin System	Irradiation/Assessment	Photostability Outcome	Supporting Evidence	Interpretation	Ref.
Fungicides						
Pyrimethanil—anilinopyrimidine fungicide	HP-β-CD; 1:1, K_S_ = 100 ± 10 M^−1^; kneaded 1:1 solid complex and aqueous systems with 30 mM HP-β-CD	Simulated solar irradiation, 280–640 nm, ~1.2 mW cm^−2^, 42 days, ultrapure/river water; HPLC-DAD; dark controls	River water: t_1/2_ 24 → 96 d and 75.9% vs. 29.4% remaining after 42 d. Ultrapure water: t_1/2_ 327 → 333 d and 97.8% vs. 90.5% remaining	Phase solubility (~5-fold solubility increase) and ^1^H NMR inclusion analysis; river-water matrix	Protection was strongly matrix-dependent: HP-β-CD had little additional effect in ultrapure water but markedly slowed photolysis in river water	[193]
Herbicides						
Bentazon—post-emergence contact herbicide	β-CD, HP-β-CD and SBE-β-CD; 1:1; K = 118 ± 20, 244 ± 19 and 317 ± 25 M^−1^; aqueous inclusion systems and solid products	Suntest xenon, λ ≥ 290 nm, 500 W m^−2^, 60 h; HPLC; dark controls	Free bentazon: DT_50_ = 17.9 h; no measurable loss with β-CD or HP-β-CD over 60 h. The 32% loss with SBE-β-CD also occurred in the dark	DPV, Job plot, phase solubility, FTIR, XRD, DTA, dissolution and surface-tension measurements	The 32% loss with SBE-β-CD also occurred in darkness, indicating a non-photochemical contribution. Despite its highest Kₐ, SBE-β-CD did not provide the greatest overall bentazon retention; solvolytic or catalytic degradation was not demonstrated.	[11]
MCPA and mecoprop (MCPP)—selective phenoxy herbicides	HP-β-CD; 1:1 assumed; K_S_ = 334 ± 9 M^−1^ (MCPA) and 282 ± 5 M^−1^ (MCPP); 35 mM aqueous inclusion systems	Simulated solar UVA/UVB, up to 130 h, ultrapure/river water; square-wave voltammetry; dark controls	t_1/2_ free → HP-β-CD: MCPA 25.9 → 80.6 h (ultrapure), 30.1 → 108.0 h (river); MCPP 20.2 → 63.6 h and 46.2 → 79.7 h	Phase solubility, UV-Vis and stability constants; river-water matrix	HP-β-CD prolonged herbicide lifetime in both media, but the magnitude of protection depended on the environmental matrix	[68]
Plant growth regulators						
2-(1-Naphthyl)acetamide (NAAm)—plant growth regulator	β-CD; 1:1, K = 651 M^−1^; in situ aqueous systems with 1–10 mM β-CD	Simulated sunlight, λ > 290 nm, 425 W m^−2^, up to 8 h; UVC 254 nm, up to 4.5 h; HPLC-PDA + LC–MS	Suntest: t_1/2_ 4.8 → 38.5 h at 10 mM β-CD; after 8 h transformation fell from 51% to 22–27%. At 254 nm, t_1/2_ 7.7 → 14.7 min	UV-Vis, steady-state/time-resolved fluorescence, Benesi-Hildebrand analysis and fluorescence quenching	β-CD produced concentration-dependent protection and altered the photochemical behavior under both simulated sunlight and UVC exposure	[194]

**Figure 20:**
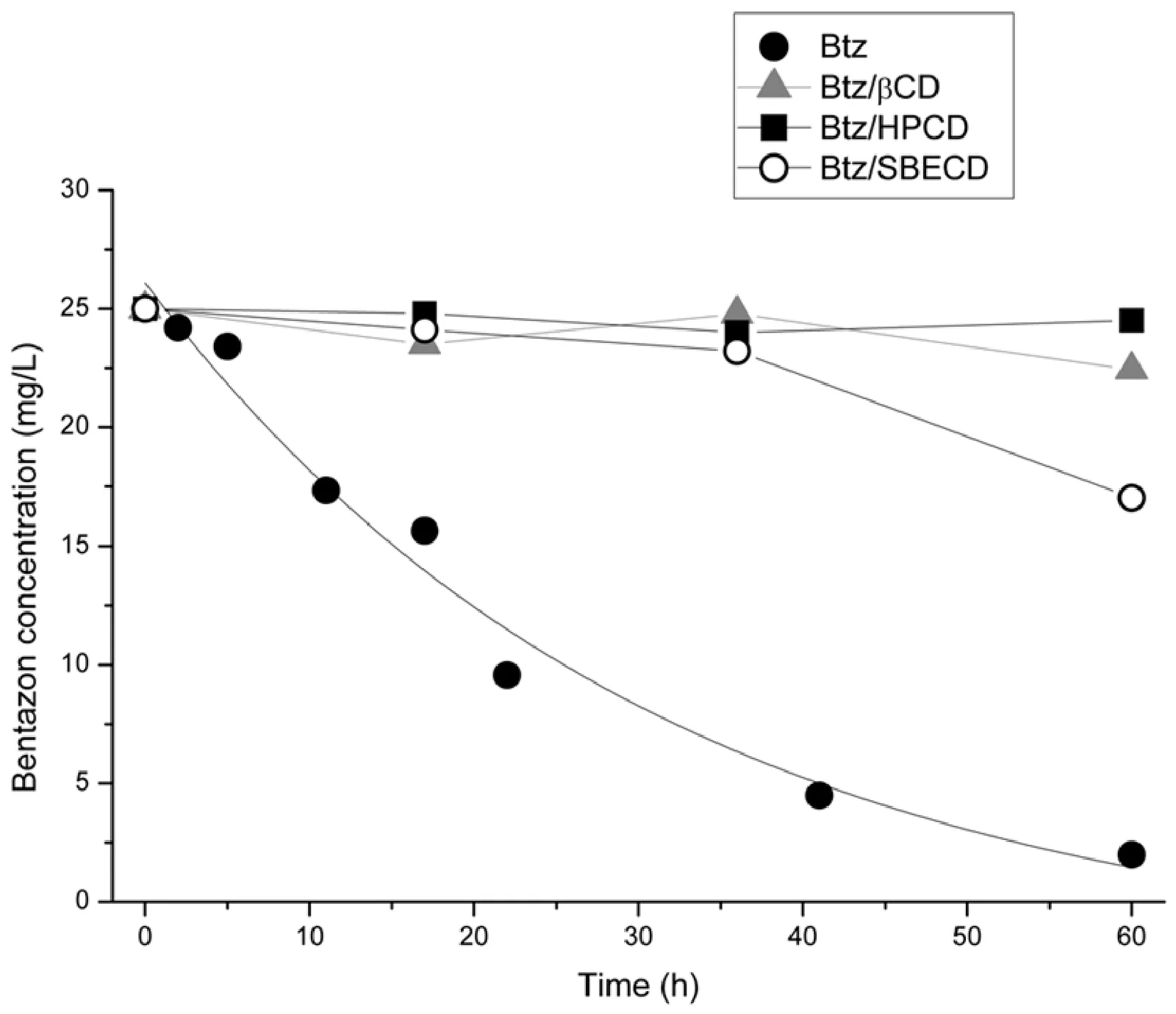
Photodegradation profiles of bentazon in distilled water in the absence and presence of different cyclodextrins under simulated solar irradiation: free bentazon (●), Btz/β-CD (▲), Btz/HP-β-CD (■), and Btz/SBE-β-CD (○). Free bentazon underwent extensive degradation over 60 h, whereas essentially no loss was observed for the β-CD- and HP-β-CD-containing systems. The approximately 32% decrease in bentazon concentration observed with SBE-β-CD after 60 h was also detected in the corresponding dark control, indicating that this loss was not specifically light-induced. This observation illustrates the importance of dark controls and demonstrates that the highest host–guest association constant did not correspond to the greatest overall chemical stability. Reproduced from Yáñez et al. (2012) [11] under CC BY 4.0.

Bentazon provides a particularly instructive example of why host–guest association strength and apparent compound loss should not be interpreted as direct predictors of photoprotection. Under simulated solar irradiation, β-CD and HP-β-CD essentially suppressed bentazon photolysis over 60 h, whereas free bentazon exhibited a DT_50_ of 17.9 h (Figure 20) [11]. Interestingly, SBE-β-CD exhibited the highest reported association constant (Kₐ = 317 ± 25 M^−1^), compared with 244 ± 19 M^−1^ for HP-β-CD and 118 ± 20 M^−1^ for native β-CD, but approximately 32% of bentazon was lost from the SBE-β-CD-containing system after 60 h. Crucially, an equivalent loss was observed in the corresponding dark control, demonstrating that the measured decrease could not be attributed specifically to photolysis. The original authors suggested that SBE-β-CD might facilitate non-photochemical decomposition, potentially influenced by elevated experimental temperatures of approximately 60 °C. However, the underlying degradation pathway was not established, and the available evidence does not demonstrate cyclodextrin-catalyzed solvolysis. This example highlights two fundamental methodological considerations: higher host–guest association constants do not necessarily predict greater photoprotection, and apparent compound loss under irradiation must be evaluated against appropriately matched dark controls before it can be assigned to photochemical degradation.

The influence of environmental water was even more striking for pyrimethanil. In ultrapure water, the free fungicide was already highly persistent, with a half-life of 327 days, and HP-β-CD produced almost no further change (333 days). In river water, however, the half-life of free pyrimethanil fell to 24 days and increased to 96 days in the presence of HP-β-CD; after 42 days, 75.9% of the complexed fungicide remained compared with 29.4% of the free compound [193]. The authors attributed the much faster degradation in river water to photochemical effects of naturally occurring constituents, including organic matter and inorganic species capable of influencing light-driven reactions. In this case, the protective contribution of HP-β-CD became most apparent only when the surrounding matrix itself promoted phototransformation.

**Figure 21:**
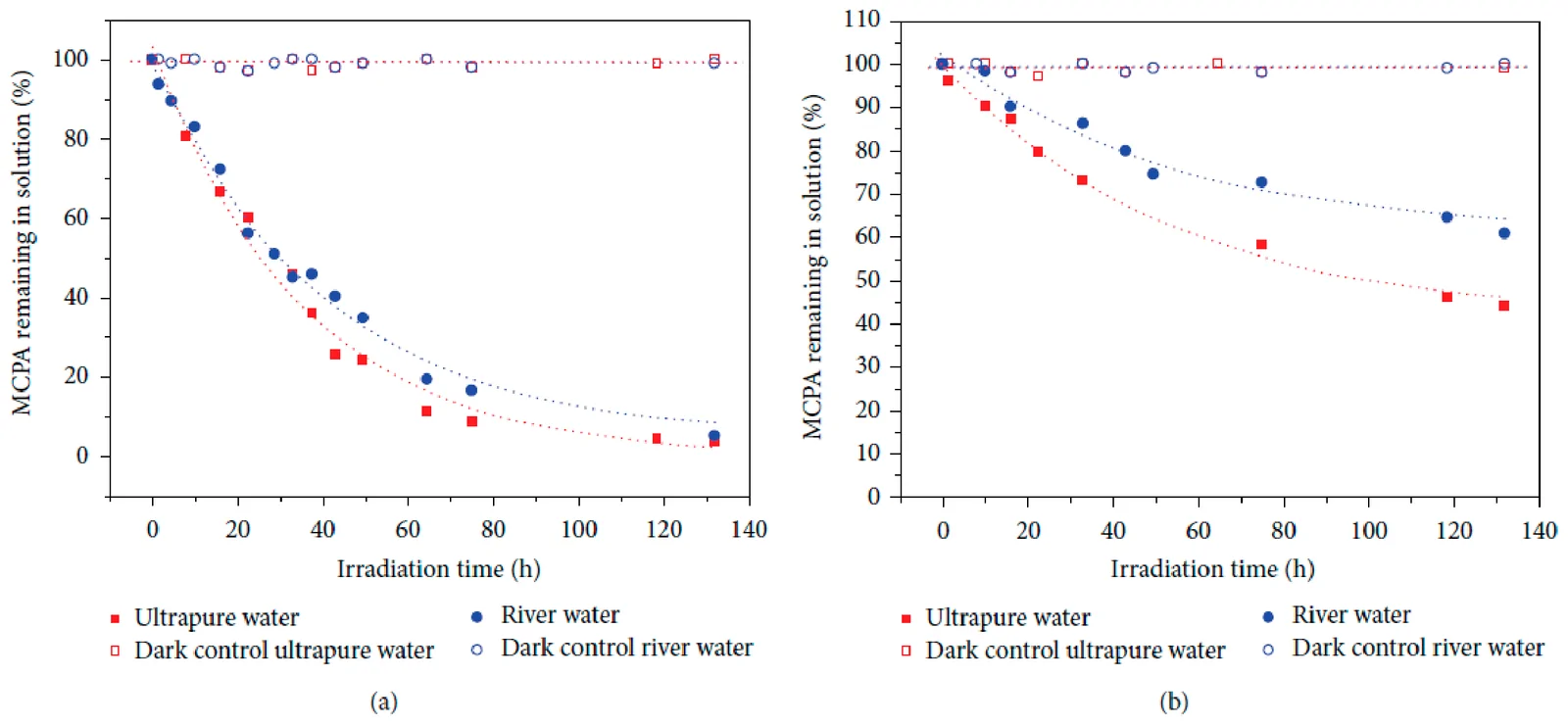
Photodegradation of MCPA in the absence and presence of HP-β-CD under simulated solar irradiation. (**a**) Free MCPA and (**b**) MCPA/HP-β-CD inclusion complex in ultrapure and river water; corresponding dark controls are shown with open symbols. HP-β-CD markedly slowed MCPA loss in both media, while the comparison between ultrapure and river water illustrates the contribution of the environmental matrix to the observed degradation kinetics. Reproduced from Costa et al. (2013) [68] under CC BY 4.0.

Natural-water matrices did not, however, affect all agrochemicals in the same direction. For MCPA and mecoprop, river water slowed photodegradation relative to ultrapure water, particularly for mecoprop, an effect attributed to attenuation of incident light by natural-water constituents. HP-β-CD nevertheless prolonged the half-lives of both herbicides in both media: from 25.9 to 80.6 h and from 30.1 to 108.0 h for MCPA in ultrapure and river water, respectively, and from 20.2 to 63.6 h and from 46.2 to 79.7 h for mecoprop (Figure 21) [68]. The contrasting behavior of pyrimethanil and the phenoxy herbicides shows that natural water cannot be assumed to affect all compounds in the same way.

Cyclodextrins can also influence the products formed during environmental photolysis. Increasing β-CD concentration slowed degradation of 2-(1-naphthyl)acetamide under simulated sunlight and increased its half-life from approximately 4.8 to 38.5 h at 10 mM β-CD [194]. The effect was concentration-dependent, with greater protection observed at higher β-CD concentrations. LC-MS comparison also showed that complexation altered the transformation pathway itself: furanone products and dihydroxylated species bearing hydroxyl groups on both aromatic rings, detected in the absence of β-CD, were not observed in the CD-containing system. For environmental applications, this distinction is important because stabilization of the parent compound and alteration of transformation pathways may have different consequences for persistence, mobility and biological activity. Cyclodextrins do not, however, always slow environmental photodegradation. In humic-water systems containing organophosphorus pesticides, CDs accelerated rather than suppressed photodegradation under artificial sunlight [195]. Such behavior is consistent with a system in which complexation can either shield the guest from reactive species or alter its exposure to photochemically generated intermediates, depending on the guest–host–matrix combination. Results obtained in purified media should therefore not be extrapolated directly to environmental conditions.

More complex formulations introduce additional uncertainty because apparent photoprotection may arise from physical attenuation of incident radiation rather than direct molecular stabilization. A recent azoxystrobin formulation combined β-CD with an alkali-lignin/sodium-alginate hydrogel and exhibited markedly improved resistance to UV exposure [196]. However, this effect may partly reflect an inner-filter and optical attenuation effect, whereby UV absorption by lignin and potential light scattering within the hydrogel matrix reduce the effective photon flux reaching the encapsulated fungicide. Such formulation-level optical protection is mechanistically distinct from genuine host–guest cavity stabilization, in which molecular inclusion modifies the local environment and photochemical behavior of the guest. The available experimental comparisons do not allow the relative contributions of β-CD inclusion, lignin-mediated UV absorption and matrix-dependent light scattering to be quantified independently. Distinguishing these mechanisms would require component-matched controls, including CD-free lignin/alginate matrices and lignin-free CD-containing formulations, together with measurements of wavelength-dependent optical transmission or reflectance under comparable irradiation conditions. Therefore, the observed stabilization should primarily be interpreted as a property of the complete multicomponent carrier rather than as direct evidence of intrinsic cyclodextrin-mediated molecular photoprotection. Such systems are considered more broadly in Section 5. A different strategy is to use light as a trigger rather than to prevent photochemical change. The γ-CD/azobenzene nanovesicles carrying pendimethalin use irradiation deliberately to trigger herbicide release rather than prevent degradation [17] and therefore belong to the photoresponsive systems discussed in Section 6.

The agrochemical studies summarized in Table 5 demonstrate that CD-mediated photostability is governed by both host–guest interactions and environmental photochemistry. The pronounced protection of bentazon [11] and the concentration-dependent stabilization of 2-(1-naphthyl)acetamide [194] are consistent with changes in guest accessibility and local environment (Section 2.1 and Section 2.3), although direct evidence for a unique shielding geometry remains limited. The contrasting photodegradation behavior of pyrimethanil, MCPA and mecoprop in ultrapure and natural waters [68,193] further highlights the importance of surrounding constituents and competing photochemical processes (Section 2.3 and Section 2.4). Changes in the photoproduct distribution of 2-(1-naphthyl)acetamide [194] suggest that complexation may also modify transformation pathways rather than simply reduce the overall degradation rate. Conversely, accelerated photolysis of organophosphorus pesticides in humic-water systems [195] illustrates the condition-dependent failure modes discussed in Section 2.5. In environmental applications, the effectiveness of CD-mediated stabilization must therefore be evaluated not only through parent-compound retention but also through biological efficacy, photoproduct formation and potential changes in environmental persistence.

Although the studies discussed above demonstrate numerous examples of CD-mediated photoprotection, the available literature also contains cases in which complexation provides no measurable benefit or even accelerates photodegradation. Representative examples from different application areas are summarized in Table 6. These findings emphasize that the effects of cyclodextrins depend on guest structure, host identity, concentration and experimental environment, and that complex formation alone does not establish photoprotective efficacy. A more comprehensive account of absent, adverse and condition-dependent outcomes, including experimental conditions and methodological limitations, is provided in Supplementary Table S2.

**Table 6:** Representative examples of absent, adverse and condition-dependent effects of cyclodextrins on photostability.

Guest/Application	CD System	Reported Photostability Outcome	Ref.
Carprofen/pharmaceutical	HP-β-CD	No significant protection; k = 0.113 ± 0.003 h^−1^ (free) vs. 0.126 ± 0.017 h^−1^ (complexed)	[113]
Tenoxicam/pharmaceutical	β-CD	Concentration-dependent reversal: modest protection at low drug concentration but accelerated photodegradation at higher concentration; drug self-association may contribute.	[29]
β-Lapachone/pharmaceutical	RM-β-CD	Stabilization in darkness but accelerated photolysis; under irradiation, t_1/2_ decreased from 2.5 to 0.5 days	[28]
Curcumin/natural bioactive	HP-β-CD	Photochemical half-life decreased from 12.8 to 7.8 min; related curcuminoids showed contrasting responses	[26]
Oxybenzone/sunscreen filter	SBE-β-CD	No significant difference in photodegradation: 2.3 ± 2.2% (free) vs. 2.9 ± 2.7% (complexed)	[177]
Organophosphorus pesticides/agrochemicals	α-, β- and γ-CD	Accelerated photodegradation in humic water; enhancement reached 45.8% for chlorpyrifos with β-CD	[195]

Note: The results were obtained under different irradiation conditions and experimental protocols and should not be compared quantitatively across studies. Detailed experimental information and additional examples are provided in Supplementary Table S2.

## 5. Advanced Cyclodextrin Architectures for Photoprotection

**Figure 22:**
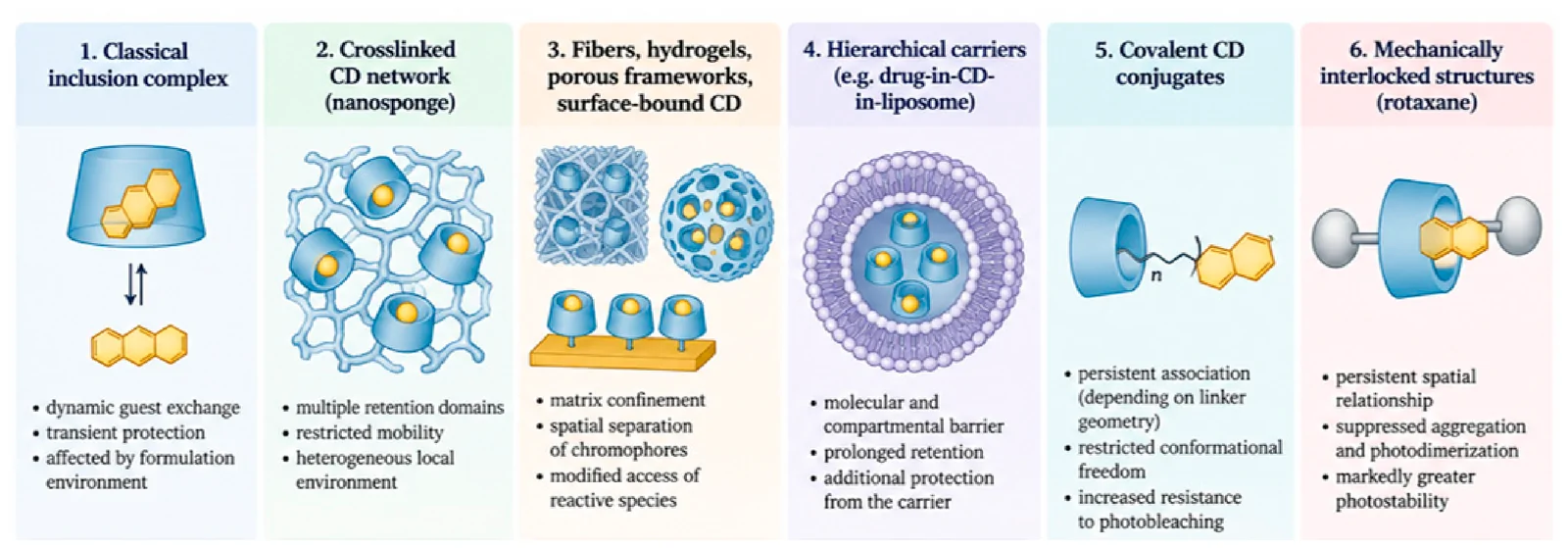
From dynamic inclusion to increasing structural control: advanced cyclodextrin architectures for photoprotection.

Classical CD inclusion complexes are dynamic systems in which guest exchange and the surrounding formulation environment can limit persistent molecular protection. Advanced architectures aim to provide greater control by incorporating CDs into crosslinked networks, fibrous or hydrogel matrices, porous frameworks, lipid carriers, nanoparticle surfaces, covalent conjugates or mechanically interlocked structures [30,54,64,159,197]. These systems can retain the guest more effectively, restrict molecular mobility or aggregation and modify access to reactive species, but their increasing complexity also makes it more difficult to separate the contribution of CD association from that of the carrier itself. The major classes of advanced CD architectures and their increasing levels of guest organization are summarized in Figure 22.

Crosslinked CD networks illustrate this transition particularly well. Unlike conventional inclusion complexes, cyclodextrin nanosponges (CDNS) contain not only the intrinsic hydrophobic cavities of the CD units but also additional interstitial, non-cavity binding domains formed between the cyclodextrin molecules and the crosslinkers. These domains exhibit heterogeneous local polarity and may accommodate guest molecules through interactions that do not involve classical cavity inclusion. The type and density of crosslinking also affect network porosity, swelling, hydration, molecular mobility and the accessibility of encapsulated guests to oxygen and other reactive species. Consequently, photoprotection in CDNS should be regarded as a collective property of the polymeric architecture rather than attributed exclusively to inclusion within individual CD cavities [24]. This distinction is illustrated by studies comparing diphenyl carbonate (DPC)- and pyromellitic dianhydride (PMDA)-crosslinked β-CD nanosponges. For curcumin, PMDA-based nanosponges provided greater apparent photoprotection than their DPC-crosslinked counterparts [130]. A similar trend was observed for resveratrol: after 8 h of UVA irradiation, the reported residual UV–Vis signal corresponded to 31.57 ± 1.00% for the free compound, 62.26 ± 1.42% for DPC-CDNS and 75.35 ± 3.07% for PMDA-CDNS [155]. These differences are consistent with an important contribution from crosslinker-dependent network organization, including potentially altered swelling, guest distribution and local microenvironments. However, the individual contributions of interstitial binding, cavity inclusion and changes in network hydration were not directly quantified, and the spectrophotometric endpoints do not independently establish complete chemical preservation of the parent compounds. PMDA-crosslinked β-CD nanosponges have also improved the photostability of BPDMA and all-trans-retinoic acid while preserving singlet-oxygen generation by the photosensitizer [30]. Further formulation-level protection has been reported after incorporation of nanosponges into Carbomer hydrogels and CD-containing systems into electrospun fibers [120,132,134].

Porous and hierarchical systems provide another level of spatial control. Muscone incorporated into a γ-CD metal–organic framework showed improved retention during prolonged light exposure [159], whereas resveratrol-loaded γ-CD-MOF/chitosan particles retained antioxidant function after irradiation, although the respective contributions of the CD framework and chitosan shell could not be separated [198]. Surface immobilization can similarly enhance optical persistence: Coumarin 30 associated with SBE7βCD immobilized on gold nanoparticles was more resistant to photobleaching than in the presence of soluble SBE7βCD [180]. In drug-in-CD-in-liposome systems, barnidipine and isotretinoin showed additional stabilization beyond that provided by liposomes alone, demonstrating the potential benefit of combining molecular inclusion with compartmental protection [31,64].

**Figure 23:**
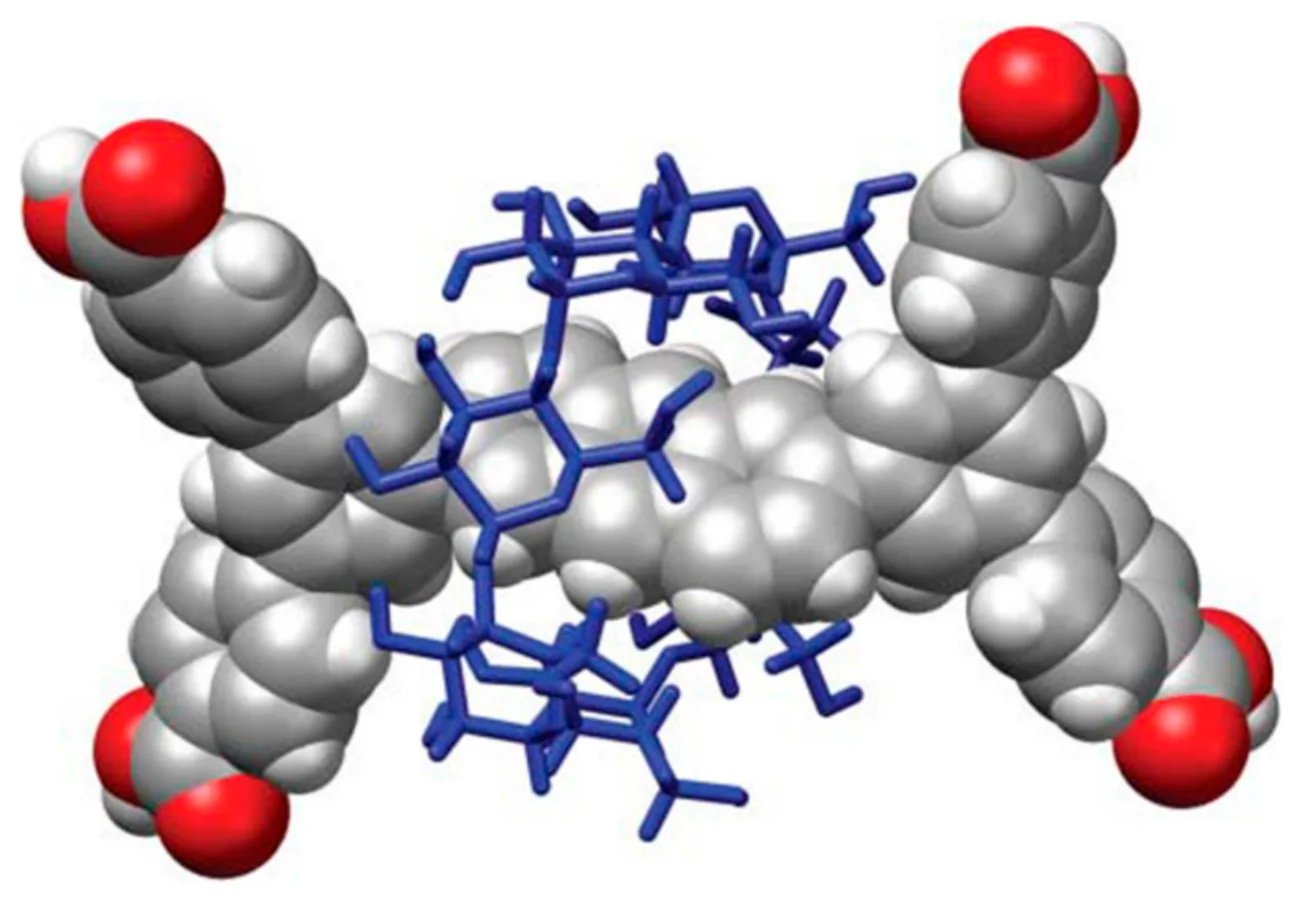
Molecular model of the β-CD-insulated anthracene rotaxane showing the cyclodextrin positioned around the photoactive anthracene unit. Bulky stopper groups prevent complete dethreading of the CD ring, maintaining its mechanical association with the chromophore-containing axle independently of dilution. This persistent molecular insulation provides a structural basis for the observed suppression of photodimerization and enhanced photostability, although the local shielding geometry may remain conformationally dynamic. Reproduced, with permission, from Stone et al. (2007) [54].

More persistent molecular protection can be achieved through covalent attachment or mechanical interlocking, which fundamentally changes the dependence of host–guest association on solution concentration. In conventional inclusion complexes, the equilibrium between free guest, free cyclodextrin and the associated species (G + CD ⇌ G·CD) is governed by the association constant and the concentrations of both components. Dilution, as may occur when formulations encounter physiological fluids or are dispersed in aqueous environments, can therefore reduce the fraction of complexed guest and diminish the associated photoprotective effect. However, the extent of dissociation depends on the binding affinity and experimental conditions. In contrast, mechanically interlocked rotaxanes contain cyclodextrin rings threaded onto molecular axles and retained by bulky stopper groups, preventing complete dethreading under conditions where the mechanical bond remains intact. Consequently, their structural association is not governed by the same intermolecular mass-action equilibrium and is not lost simply through dilution. This distinction should not be described as an infinite local binding constant, since mechanical interlocking imposes a topological constraint rather than an infinitely favorable thermodynamic association. Covalently linked pseudo[1]rotaxanes provide a related but distinct approach: the host and guest remain connected within the same molecule, although reversible conformational changes may expose or shield the photoreactive region [34]. β-CD conjugates of cyanine dyes and Pacific Blue have shown improved photostability, with the magnitude depending on linker geometry and molecular organization [32,183]. Mechanically interlocked cyanine and anthracene rotaxanes exhibited markedly greater resistance to photobleaching than corresponding uninsulated structures, while the β-CD-insulated anthracene rotaxane also suppressed photodimerization [48,54]. Related porphyrin-based [5]rotaxanes demonstrated that persistent CD insulation can improve chromophore durability while retaining the photochemical activity required for singlet-oxygen generation [33]. The structure of the β-CD-insulated anthracene rotaxane is shown in Figure 23. Nevertheless, persistent mechanical association does not necessarily guarantee complete or permanent shielding of a particular reactive site, since the position and mobility of the threaded macrocycle and the resulting molecular environment also influence photochemical behavior.

Architectural complexity does not, however, guarantee greater photoprotection. Crosslinked CD polymers have produced protective, negligible or adverse effects depending on crosslinker chemistry, solvent and oxygen availability [61,176]. Likewise, in multicomponent systems containing polymers, nanoparticles or other stabilizing elements, substantial overall protection cannot automatically be assigned to the cyclodextrin. The most informative studies therefore compare the free guest, the CD-associated guest, a matched CD-free carrier and the complete CD-containing architecture whenever such controls are experimentally feasible.

Advanced CD architectures are most valuable when they provide a demonstrable photochemical advantage beyond classical inclusion. Crosslinked networks and polymeric matrices can improve guest retention, porous and surface-bound systems provide additional organization, hierarchical carriers combine inclusion with compartmental protection, and covalent or interlocked structures maintain a more stable CD–guest relationship [30,54,64,132]. Their usefulness should therefore be assessed by the additional control they provide over guest location, mobility and reactive pathways rather than by structural complexity itself.

## 6. Cyclodextrins in Photoactive and Photoresponsive Systems

**Figure 24:**
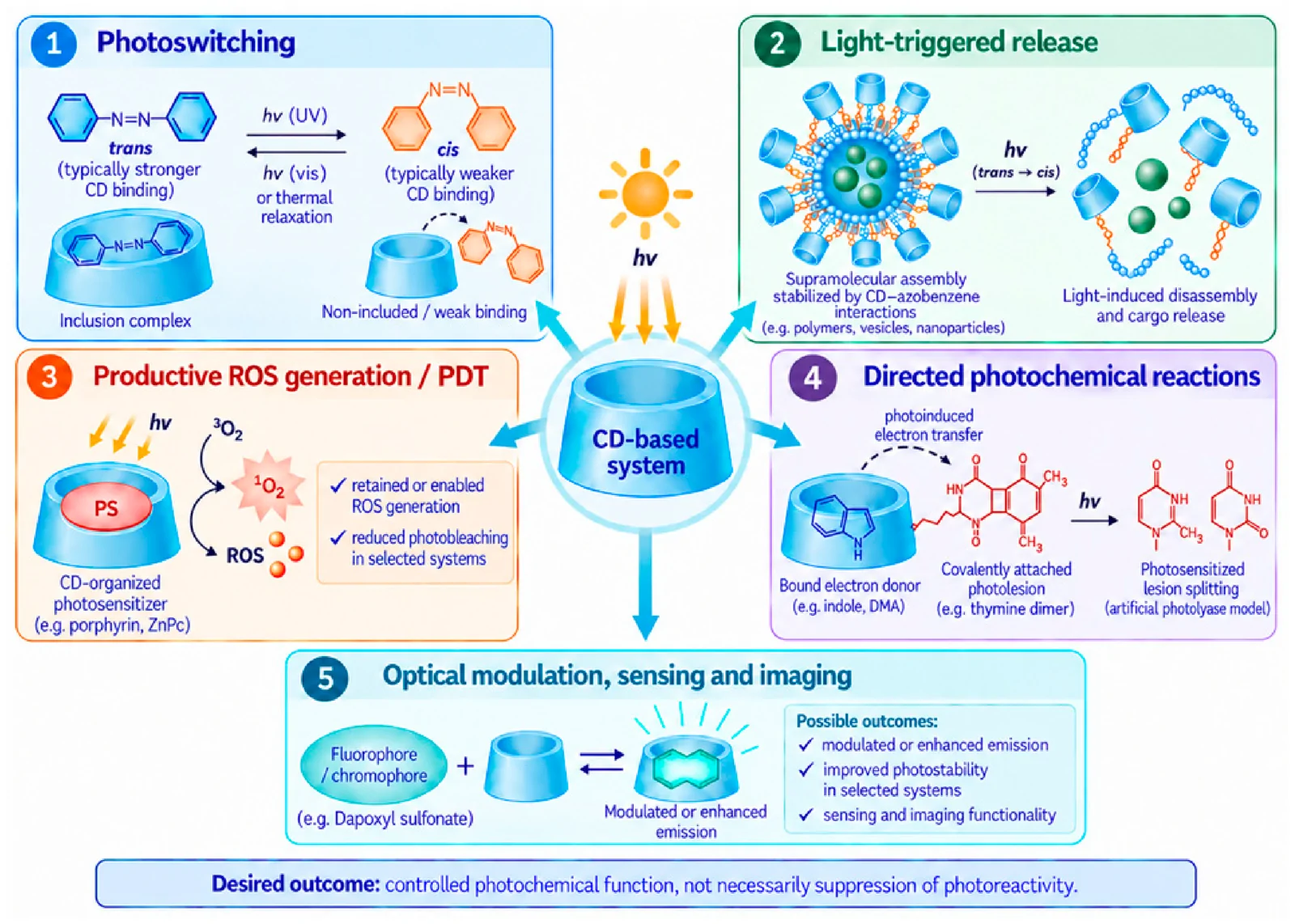
Cyclodextrins as modulators of controlled photochemical function in photoactive and photoresponsive systems. Azobenzene-mediated photoresponsive release is represented schematically; the actual response is governed by the wavelength-dependent E/Z photostationary state, reversible host–guest association and carrier-specific release kinetics, and does not necessarily involve complete molecular switching or quantitative cargo release.

The influence of cyclodextrins on light-driven processes extends beyond suppression of unwanted degradation. In photoactive and photoresponsive systems, irradiation is deliberately used to alter host–guest association, trigger cargo release, generate reactive oxygen species or heat, or modulate optical output. Photochemical transformation is therefore part of the intended function rather than a process to be minimized. In these systems, the role of a CD may range from direct control of a photoreactive guest to organization of a larger supramolecular architecture in which light changes association, accessibility or reactivity. Representative systems are summarized in Supplementary Table S4, and the principal roles of cyclodextrins in photoactive and photoresponsive systems are illustrated in Figure 24.

Reversible CD-azobenzene recognition is one of the most established strategies for converting light into supramolecular motion or release. In sulfonated α-CD-containing polyelectrolyte films, azobenzene residues underwent reversible E/Z photoisomerization, and inclusion significantly affected both photochemical and thermal isomerization [15]. The response was host-dependent: sulfonated α-CD interacted with the azobenzene residues, whereas the corresponding sulfonated β-CD system showed no comparable evidence of inclusion. Related systems exploit the weaker association of the cis form with the CD host to destabilize supramolecular assemblies. In poly(β-CD)/azobenzene gene-delivery complexes, UV irradiation promoted polyplex dissociation and DNA release [16]. A different strategy used light-triggered competition between two guests: photocleavage exposed adamantane groups whose stronger affinity for β-CD displaced the host from biotin, increasing surface exposure of the targeting ligand from approximately 14.6% to 77.7% [199].

The same azobenzene–CD principle has also been adapted to agrochemical delivery. In γ-CD/azobenzene nanovesicles, trans-to-cis photoisomerization destabilized the host–guest assembly and triggered pendimethalin release. Cumulative pendimethalin release reached 88.3 ± 3% under 365 nm UV or sunlight exposure, approximately 4.3 times the value observed in darkness [17]. Here, photochemical destabilization, rather than stabilization, is the intended function of the CD-containing architecture.

An important limitation of azobenzene-mediated release is the establishment of a photostationary state (PSS) under continuous irradiation. Because both E-to-Z and Z-to-E photoisomerization can occur under illumination, the system generally approaches a wavelength-dependent steady-state distribution of the two isomers rather than undergoing complete conversion to the Z form. The PSS composition depends on the absorption properties and photoisomerization quantum yields of both isomers, irradiation wavelength and intensity, solvent conditions and temperature. Thermal Z-to-E relaxation may additionally contribute during irradiation and becomes particularly relevant after the light source is removed. Since the E and Z isomers generally exhibit different affinities for cyclodextrin cavities, the residual E-isomer population and subsequent thermal recovery may permit renewed host–guest association and partial reassembly of the supramolecular structure. Consequently, irradiation does not necessarily induce complete dissociation of all inclusion complexes, and the reversibility of the response may influence repeated switching cycles. However, the PSS composition should not be equated directly with the cumulative percentage of cargo released, since diffusion, irreversible vesicle disruption and subsequent transport of the released cargo can also determine the final release efficiency. For the pendimethalin-loaded γ-CD/azobenzene nanovesicles, the reported cumulative release of approximately 88.3 ± 3% under UV or sunlight exposure, compared with approximately 20.5% in darkness, demonstrates substantial but incomplete release under the investigated conditions [17]. The original studies did not quantitatively establish the relationship between PSS composition, host–guest reassociation and cargo release over repeated irradiation–dark cycles. Future investigations should therefore combine time-resolved E/Z isomer analysis with binding measurements and repeated-cycle release experiments to distinguish reversible molecular switching from irreversible carrier disassembly and cargo escape.

**Figure 25:**
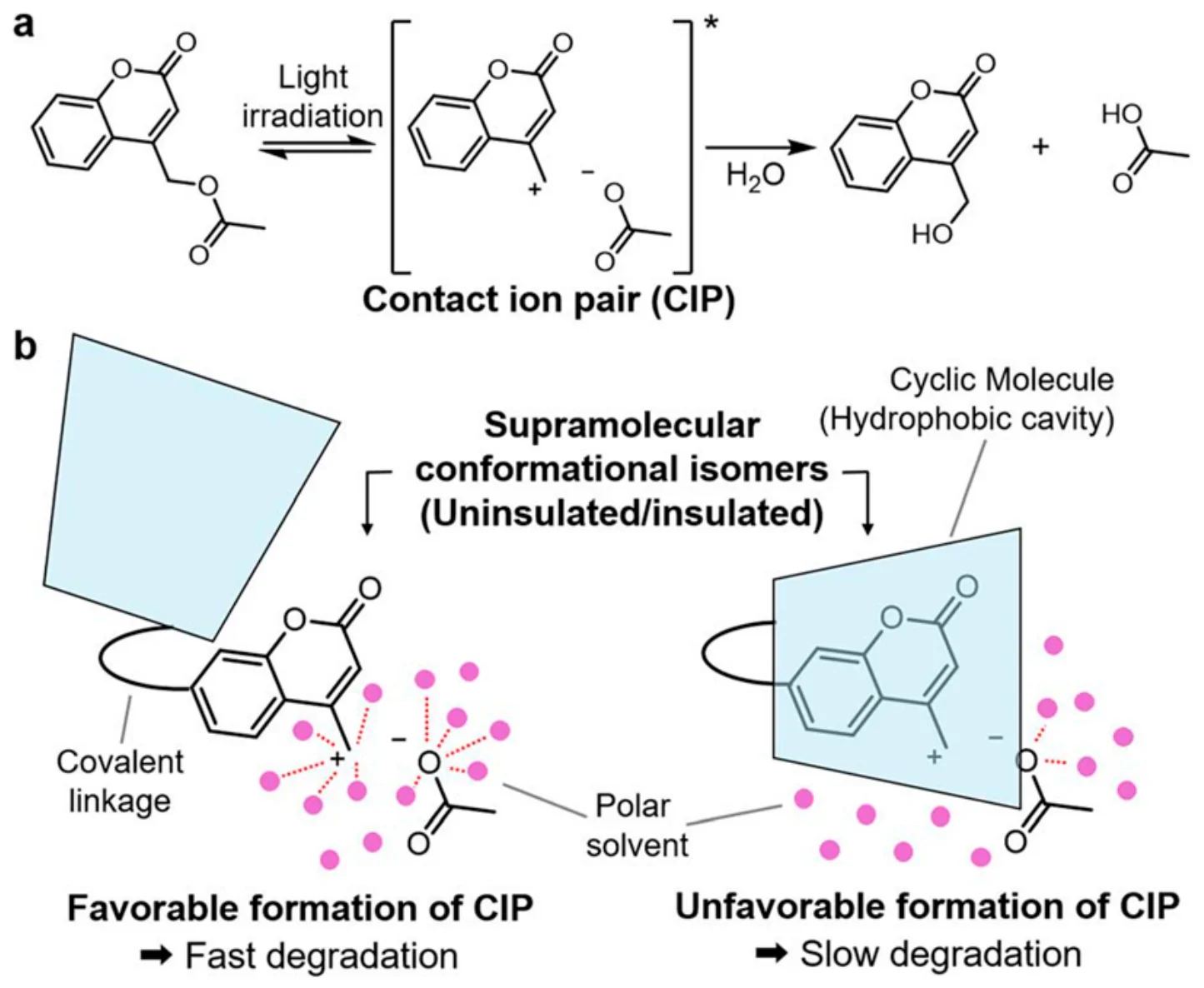
Supramolecular control of coumarinylmethyl-ester photolability in a PM-α-CD pseudo[1]rotaxane. Changes in solvent conditions and heating enable reversible switching between exposed and CD-insulated conformations. The insulated state shows reduced photocleavage under irradiation because the hydrophobic CD environment decreases stabilization of the polar contact-ion-pair intermediate. Reproduced from Masai et al. (2025) [34] under CC BY 3.0.

A different implementation was reported by Masai and co-workers using a PM-α-CD pseudo[1]rotaxane. Unlike permanently stoppered rotaxanes, this covalently connected pseudo[1]rotaxane permits reversible intramolecular switching between insulated and exposed conformations. Thus, dilution does not separate the host and guest components, but changes in solvent conditions and temperature can still modify the degree of chromophore shielding [34]. Changes in solvent polarity combined with heating reversibly switched the coumarinylmethyl ester between insulated and exposed states, allowing the same photolabile unit to alternate between relatively photostable and photoreactive behavior. Under identical irradiation conditions, photodegradation after 90 min was approximately 70% for the uninsulated structure but only about 20% for its PM-α-CD-insulated counterpart. The authors related this difference to the hydrophobic cavity environment, which reduces polar stabilization of the contact ion-pair intermediate involved in photocleavage [34]. The molecular effect was translated to the materials level: polymer networks containing the uninsulated crosslinker lost shape and storage modulus under 365 nm irradiation, whereas the corresponding insulated networks remained markedly more resistant. This system therefore links photoprotection with controlled switching of photoreactivity. The molecular basis of this switch between exposed and CD-insulated states is illustrated in Figure 25.

Cyclodextrins can also regulate productive excited-state chemistry rather than suppress it. Methylated β-CD promoted monomerization of pheophorbide *a* and enabled singlet-oxygen generation in aqueous medium, whereas structurally related far-red sensitizers responded differently: a Zn-naphthalocyanine required elevated temperature for efficient deaggregation, and the tested Zn-phthalocyanine remained aggregated. The contrasting responses of these sensitizers demonstrate that CD-mediated deaggregation can facilitate singlet-oxygen generation in selected systems, although the outcome depends strongly on the molecular structure of the photosensitizer [13].

More elaborate architectures can combine productive photochemistry with substantial protection of the active chromophore. In PPIX-bridged vesicles assembled through β-CD/azobenzene recognition, free PPIX lost approximately 25.2% of its Soret-band absorbance after 10 min of intense UV irradiation, compared with about 8% for vesicle-associated PPIX; after 60 min, degradation of the vesicle-associated sensitizer remained about 26%. The same assemblies exhibited evidence consistent with retained singlet-oxygen-generating activity in an indirect iodide-based assay, although the singlet-oxygen quantum yield (ΦΔ) was not quantitatively determined [200]. Because PPIX-2CD formed part of a hierarchical vesicle together with azobenzene-functionalized polymers, however, this protection should be attributed to the complete supramolecular architecture rather than to cavity inclusion alone.

For photosensitizers intended for photodynamic therapy, suppression of photobleaching alone is insufficient to establish improved functional photostability. The preservation of productive photochemistry, particularly singlet-oxygen generation in Type II photodynamic processes, must also be demonstrated. Ideally, the singlet-oxygen quantum yield (ΦΔ) should be determined using time-resolved near-infrared phosphorescence detection at approximately 1270 nm or an appropriately calibrated indirect assay. Electron paramagnetic resonance (EPR/ESR) spectroscopy with suitable spin-trapping probes, such as the TEMP/TEMPO system, can provide complementary evidence of singlet-oxygen formation, although potential probe-related side reactions require appropriate controls. Importantly, photobleaching and reactive-oxygen-species generation should be evaluated under comparable experimental conditions, with particular attention to excitation wavelength, absorbed photon dose, photosensitizer concentration, solvent or formulation medium, temperature and oxygen availability. Where possible, singlet-oxygen production should also be monitored over the irradiation period to determine whether photodynamic functionality is retained as the chromophore undergoes photobleaching. Studies of TMPyP-containing cyclodextrin assemblies illustrate the importance of this distinction, since different formulations have exhibited increased or decreased ΦΔ despite improved chromophore stability [188,189]. Accordingly, evidence of reduced photobleaching without a corresponding assessment of productive photochemistry should be interpreted as improved optical stability rather than demonstrated enhancement of photodynamic performance. 

A different type of stabilization was obtained when permethylated β-CD-ZnPc conjugates were immobilized on adamantane-functionalized silica through multivalent host–guest recognition. The free CD-ZnPc conjugates in water underwent almost complete Q-band photobleaching within about 3 h, whereas the immobilized assemblies remained nearly unchanged after 6 h and retained recyclable photooxygenation and photocatalytic activity [14]. Here, CD served primarily as a supramolecular anchoring element rather than as a classical inclusion host for the photosensitizer itself.

Cyclodextrins may also organize reactants so that light drives a desired chemical transformation. In an artificial photolyase model, modified β-CD brought indole or *N,N*-dimethylaniline into proximity with covalently attached thymine photolesions, enabling efficient photosensitized splitting through an electron-transfer pathway and suppressing back electron transfer [201]. Control experiments showed that the supramolecularly organized systems were more efficient than corresponding nonorganized reactions, supporting the role of host–guest proximity in directing the photochemical pathway. Such systems illustrate how CDs can act as supramolecular organizers of photochemical reaction pathways rather than simply protective hosts.

The much broader set of examples summarized in Supplementary Table S4 also includes fluorescent probes, upconversion systems, metal nanoclusters, quantum dots and theranostic carriers. In these platforms, however, the role of cyclodextrins must be distinguished from direct modulation of guest photochemistry through cavity inclusion. In rhodamine-based upconversion sensing systems, α-CD primarily functions as a hydrophilic phase-transfer and surface-modifying agent, enabling the aqueous dispersion of oleate-coated NaYF_4_^3+^/Er^3+^ nanocrystals and their compatibility with organic chemosensors [18,19]. The improved photostability of the rhodamine probes is associated mainly with indirect excitation through energy transfer from the upconversion nanocrystals under 980 nm near-infrared irradiation, thereby avoiding direct high-energy excitation of the organic fluorophores. The available evidence does not establish that α-CD directly shields the rhodamine chromophore within its cavity or independently suppresses its photochemical degradation. Similarly, in β-CD-modified, cysteine-protected copper nanoclusters, the cyclodextrin primarily contributes to colloidal stabilization, aqueous dispersibility and the surface environment of the luminescent CuNCs [202]. Although such surface modification may influence emission efficiency and photostability through changes in surface passivation or aggregation, these effects should not be confused with molecular photoprotection of an included guest. Consequently, the improved optical stability reported for these systems should be attributed principally to their excitation strategy or nanoparticle architecture, rather than to experimentally demonstrated cyclodextrin cavity-mediated shielding. These examples illustrate the importance of distinguishing cyclodextrins acting as solubilizing or colloidal stabilizing agents from those directly controlling the photochemical behavior of molecular guests.

Photoactive and photoresponsive systems therefore require different criteria from conventional photostability studies. Reduced photobleaching may be desirable for a photosensitizer, whereas efficient isomerization, bond cleavage, supramolecular disassembly or cargo release may be the intended outcome in another system. The relevant question is not whether a cyclodextrin suppresses photochemistry, but whether host–guest recognition, molecular insulation or supramolecular organization directs the absorbed light toward the intended function while limiting competing destructive pathways. CD-mediated photoprotection and photoresponsiveness can therefore be viewed as different applications of the same ability to control the local environment of a photoreactive system.

## 7. Future Perspectives

**Figure 26:**
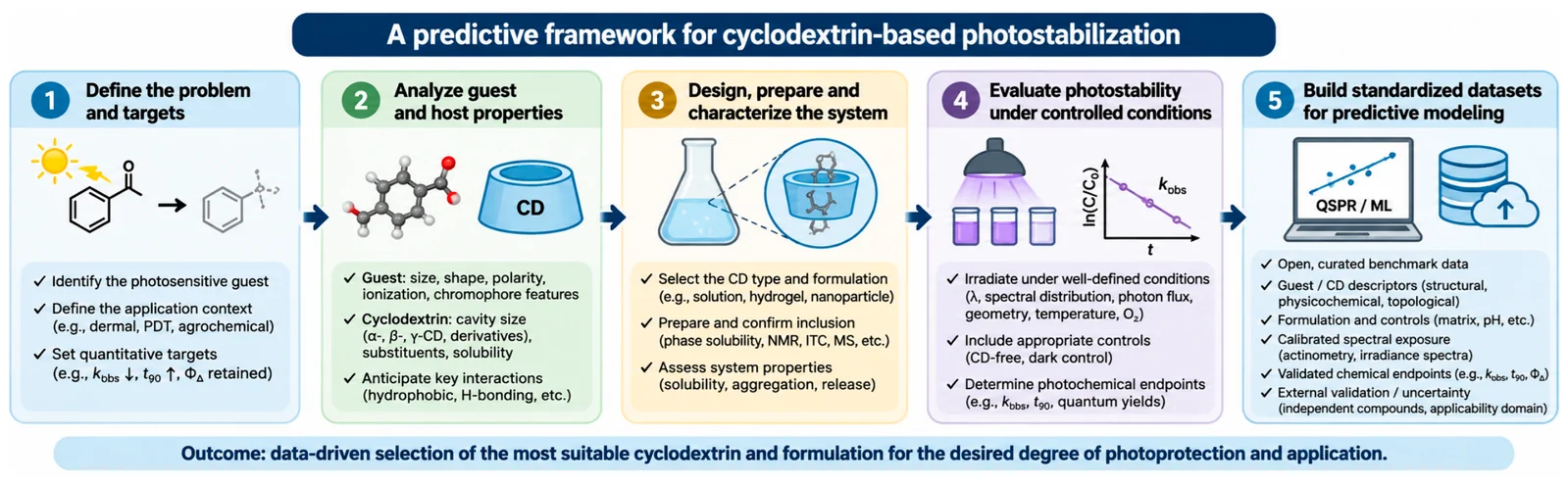
Proposed five-step framework for predictive design of cyclodextrin-mediated photostability. The workflow integrates identification of photochemical degradation pathways, characterization and modeling of host–guest interactions, controlled irradiation experiments, validation in the intended formulation, and construction of standardized benchmarking datasets. The final stage emphasizes curated open-access data, calibrated irradiation conditions, validated analytical endpoints, and external validation of QSPR and machine-learning models to support reproducible, mechanism-guided cyclodextrin selection.

Future research on cyclodextrin-mediated photostability should move beyond empirical host screening toward more predictive design. Binding strength alone is not sufficient to predict photoprotection, and the outcome may change with host identity, guest concentration, solvent or formulation environment [27,29,50,59]. Host selection should therefore begin with the photochemical pathway that needs to be controlled and with the structural features of the guest, its photochemical behavior under the intended conditions of use. The proposed framework for predictive design of cyclodextrin-mediated photostability and photochemical behavior is shown in Figure 26.

A central challenge for predictive cyclodextrin design is to establish a quantitative connection between ground-state host–guest organization and the excited-state pathways responsible for photochemical transformation. Association constants, binding energies and equilibrium inclusion geometries provide information about ground-state complexation but cannot independently predict the fate of a guest following photon absorption. Future studies should therefore integrate time-dependent density functional theory (TD-DFT) and hybrid quantum mechanics/molecular mechanics (QM/MM) calculations with time-resolved spectroscopic measurements [39,74]. Molecular dynamics simulations can first be used to sample representative inclusion geometries, guest orientations and hydration environments. Subsequent TD-DFT calculations, preferably incorporating the surrounding cyclodextrin and solvent environment through suitable QM/MM models, could then provide estimates of vertical excitation energies, oscillator strengths, electronic-state character and relevant regions of the excited-state potential energy surfaces. Comparisons between free and complexed guests may help identify whether confinement changes the accessibility of pathways associated with non-radiative relaxation, intramolecular or intermolecular charge transfer, intersystem crossing or photochemical bond cleavage. Such predictions should be tested against ultrafast transient absorption measurements with femtosecond or sub-picosecond time resolution, which can probe early excited-state relaxation and charge-transfer processes, and complementary laser flash photolysis experiments, which are particularly useful for characterizing longer-lived triplet states, radical intermediates and their subsequent reactions. Importantly, conventional linear-response TD-DFT may be insufficient for describing conical intersections, strong multireference character or homolytic bond dissociation, and more advanced electronic-structure methods or nonadiabatic dynamics simulations may be required in such cases. The combination of computational excited-state screening and experimentally resolved photochemical kinetics could provide a mechanistic basis for prioritizing cyclodextrin hosts and inclusion geometries before extensive synthesis or formulation optimization, while recognizing that reliable prediction of photochemical branching ratios and quantum yields still requires experimental validation.

More information should also be collected during irradiation rather than only before and after exposure. Self-association, aggregation, oxygen availability and the relative populations of free and complexed species may change as irradiation proceeds, particularly in concentration-dependent or multicomponent systems [12,29,61,176]. Time-resolved or continuously sampled monitoring of parent concentration, optical properties and reactive intermediates could reveal pathway changes that would be missed by pre- and post-irradiation analysis.

Predictive design must ultimately extend from the molecular complex to the relevant formulation environment. Solvent composition can alter the protective effect of β-CD on avobenzone, while hierarchical systems such as drug-in-CD-in-liposome formulations may provide stabilization beyond molecular complexation alone [31,59,64]. Advanced architectures are most valuable when they provide a defined photochemical advantage, for example, by prolonging confinement, restricting intermolecular contact or enabling reversible control over photochemical accessibility [17,30,33,34,132]. A staged strategy is therefore preferable: first establish the molecular guest–CD effect, then test it in the relevant matrix, and finally determine whether it persists in the complete formulation using appropriate carrier-matched controls.

The desired degree of stabilization should remain application-specific. Pharmaceuticals generally require preservation of the parent compound, UV filters must retain their absorption properties, fluorescent probes must preserve useful emission, and photosensitizers must remain sufficiently stable without losing productive ROS generation [12,25,30,33]. A major prerequisite for reliable quantitative structure–property relationship (QSPR) and machine-learning (ML) models is the availability of standardized, carefully curated photostability datasets. The existing literature is highly heterogeneous with respect to irradiation wavelength and spectral distribution, incident photon flux, lamp geometry, sample configuration, temperature control, solvent composition, oxygen availability and analytical endpoints. Consequently, apparent photodegradation rate constants (kobs) obtained under different experimental conditions cannot be treated as directly interchangeable measures of intrinsic molecular photostability. Training predictive algorithms on the uncurated literature values risks introducing systematic experimental biases, potentially causing models to reproduce differences between laboratories and irradiation protocols rather than genuine structure–photostability relationships. A priority for future research should therefore be the establishment of open-access benchmarking datasets generated using harmonized irradiation protocols, with photon flux verified by suitable chemical actinometry and, where appropriate, spectrally resolved irradiance measurements. Such datasets should include matched CD-free and dark controls, validated parent-compound assays, clearly defined kinetic models, experimental uncertainties and complete metadata describing the guest, cyclodextrin, formulation and exposure conditions. The retrospective literature data may still support exploratory modeling when sufficiently documented and critically curated, but should not be pooled indiscriminately. Predictive models should additionally undergo external validation using independent compounds and, preferably, data from laboratories not represented in the training set, with explicit assessment of prediction uncertainty and applicability domain. Only after these requirements have been addressed can QSPR and ML approaches provide a reliable basis for predictive cyclodextrin host selection rather than merely fitting heterogeneous experimental observations.

## 8. Conclusions

Cyclodextrins can markedly alter the photochemical fate of molecular guests, but the formation of a stable inclusion complex does not, by itself, predict whether photostabilization will occur. An experimentally determined association constant or a well-defined inclusion geometry provides evidence of ground-state host–guest organization, but neither is sufficient to establish the direction or magnitude of the resulting photochemical response. As demonstrated by the studies reviewed here, cyclodextrin complexation may suppress photodegradation, produce no measurable change, or even accelerate photochemical transformation. These contrasting outcomes depend on the complete guest–host–environment system, including the position and mobility of the photoreactive region, host identity and substitution, guest self-association, solvent and oxygen conditions, physical state and formulation architecture. The decisive factor is therefore not the existence or thermodynamic stability of the inclusion complex itself, but how the resulting supramolecular environment modifies competing excited-state relaxation and photochemical reaction pathways. Cyclodextrins should consequently be viewed not as passive shields against light, but as molecular regulators of photochemical fate whose effects must be experimentally established under defined conditions.

Accordingly, successful CD-mediated photoprotection cannot be defined simply as maximal suppression of photochemical change. The relevant outcome depends on the intended function of the guest and may involve preservation of chemical integrity, spectral or biological performance, suppression of undesirable photoproducts, or controlled retention of productive photoreactivity. For photoactive systems, preserving useful photochemistry may be just as important as limiting degradation. Advanced and photoresponsive CD architectures extend this principle beyond passive stabilization toward deliberate regulation of photochemical accessibility and function.

The strength of any photostability claim must likewise remain consistent with the evidence on which it is based. Direct preservation of the parent compound, formulation-level stabilization, functional persistence and mechanistic support represent distinct levels of information rather than interchangeable measures of photoprotection. Neutral and adverse outcomes are equally informative, because they define the conditions under which CD association fails to protect the guest or redirects photochemistry toward an unfavorable pathway.

The available evidence therefore argues strongly against treating cyclodextrins as universally photoprotective hosts. Their real value lies in their ability to modify the local molecular environment and thereby shift the balance among competing excited-state, reactive-species and degradation pathways. The central design question is therefore not whether a cyclodextrin can protect a guest, but which host or CD-based architecture can direct its photochemical behavior toward the desired outcome under defined molecular, environmental and formulation conditions. Moving from empirical complexation toward such mechanism-guided control is the key step toward predictive design of CD-mediated photostability.

To facilitate the critical evaluation and reproducibility of future studies, we propose the following five-point quality checklist for researchers, reviewers and editors assessing cyclodextrin-mediated photostability:

- Stability-indicating analytical methods. Claims of chemical photoprotection should be supported by direct quantification of the intact parent compound using validated, stability-indicating methods, preferably HPLC or UPLC capable of resolving the parent molecule from its structural degradation products and geometric photoisomers. UV–Vis absorbance retention alone should not be considered definitive evidence of chemical stability.
- Mandatory dark controls. Irradiated samples should be compared with parallel, temperature-matched, non-irradiated controls prepared under otherwise identical conditions. Such controls are essential for distinguishing genuine photochemical degradation from thermal decomposition, hydrolysis, solvolysis and other light-independent processes.
- Solvent- and phase-matched comparators. Free and CD-complexed guests should be evaluated in comparable solvent, pH, oxygen and physical-state environments, with appropriate CD-free vehicle and carrier controls. Comparisons involving different solvents or physical phases should be explicitly identified as potentially confounded, since apparent stabilization may arise from the formulation environment rather than host–guest inclusion.
- Standardized radiometric reporting. Irradiation conditions should be documented comprehensively, including the spectral distribution of the light source, wavelength range, calibrated irradiance (W m^−2^), radiant exposure (J cm^−2^ or equivalent units), irradiation geometry, exposure duration and sample temperature. Chemical actinometry should be used where appropriate to verify photon exposure, while ICH Q1B metrics should be reported when relevant to pharmaceutical photostability assessment.
- Multi-point kinetic verification. Photodegradation should be monitored at multiple time points using replicate samples, with appropriate statistical analysis and evaluation of kinetic-model goodness-of-fit and residuals. Where relevant, experiments should account for deviations from simple first-order behavior arising from induction periods, changes in host–guest association, concentration-dependent aggregation, oxygen depletion or competing photochemical pathways. Rate constants, uncertainties and the limitations of the selected kinetic models should be reported transparently.

Together, these criteria provide a practical framework for distinguishing genuine molecular photoprotection from spectroscopic artifacts, non-photochemical degradation and formulation-dependent effects. Their systematic application would improve the reproducibility, comparability and mechanistic reliability of cyclodextrin photostability research.

## Data Availability

No new data were created or analyzed in this study. Data sharing is not applicable to this article.

## Supplementary Materials

The following supporting information can be downloaded at: https://www.mdpi.com/article/10.3390/ijms272110040/s1. References [203,204,205,206,207,208,209,210,211,212,213,214,215,216,217,218,219,220,221,222,223,224,225,226,227,228,229,230,231,232,233,234,235,236,237,238,239,240,241,242,243,244,245,246,247,248,249,250,251,252,253] are cited in the supplementary materials.

## Abbreviations

The following abbreviations are used in this manuscript:

Abbreviation	Full term
2-HP-β-CD	2-hydroxypropyl-β-cyclodextrin
4-MBC	4-methylbenzylidene camphor
AA	ascorbic acid
ABTS	2,2′-azino-bis(3-ethylbenzothiazoline-6-sulfonic acid)
AFM	atomic force microscopy
AgNPs	silver nanoparticles
AIE	aggregation-induced emission
AIPE	aggregation-induced phosphorescence emission
ANOVA	analysis of variance
aPDT	antimicrobial photodynamic therapy
API	active pharmaceutical ingredient
ATR-FTIR	attenuated total reflectance Fourier-transform infrared spectroscopy
ATRA/atRA	all-trans-retinoic acid
AuNPs	gold nanoparticles
AVB/AVO	avobenzone
AX	astaxanthin
Az/AZO/Azo	azobenzene
Az-BPDM	azobenzene-terminated branched poly[2-(dimethylamino)ethyl methacrylate]
Az-LPDM	azobenzene-terminated linear poly[2-(dimethylamino)ethyl methacrylate]
Az-PAH	azobenzene-modified poly(allylamine hydrochloride)
AzoPEO	azobenzene-terminated poly(ethylene oxide)
AZO-HBPO	azobenzene-functionalized hyperbranched poly(3-ethyl-3-oxetanemethanol)
AZO-HPG	azobenzene-functionalized hyperbranched polyglycerol
BCS	Biopharmaceutics Classification System
BET	Brunauer–Emmett–Teller
BHA	butylated hydroxyanisole
BHT	butylated hydroxytoluene
BMD	4-tert-butyl-4′-methoxydibenzoylmethane
BMDBM	butyl methoxydibenzoylmethane (avobenzone)
BPDMA	benzoporphyrin derivative monoacid ring A
BRA	black-rice anthocyanin extract
BSA	bovine serum albumin
BSAF	bovine serum albumin fibrils
BZ-3	benzophenone-3 (oxybenzone)
C30	Coumarin 30
CD	cyclodextrin
CD-MOF	cyclodextrin-based metal–organic framework
CDNS	cyclodextrin nanosponge
CFU	colony-forming unit
CIELAB	Commission Internationale de l’Éclairage L*a*b* color space
CLSM	confocal laser scanning microscopy
CMCSMA	carboxymethyl chitosan methacrylate
CNT	carbon nanotube
CoQ10	coenzyme Q10
COPD	chronic obstructive pulmonary disease
CPZ	chlorpromazine
CRYSMEB	methylated β-cyclodextrin marketed as Kleptose Crysmeb^®^
CUR	curcumin
Cys	cysteine
D-1NA	3-(1-naphthyl)-D-alanine hydrochloride
DA	dopamine
DAD	diode-array detector
DBM	dibenzoylmethane
DEASPI	trans-4-[p-(N,N-diethylamino)styryl]-N-methylpyridinium iodide
DEVD	Asp–Glu–Val–Asp peptide sequence
DFT	density functional theory
DHP	1,4-dihydropyridine
DLS	dynamic light scattering
DM-β-CD	dimethyl-β-cyclodextrin
DMSO	dimethyl sulfoxide
DNA	deoxyribonucleic acid
DOSY	diffusion-ordered spectroscopy
DOX	doxorubicin
DP	degree of polymerization
DPBF	1,3-diphenylisobenzofuran
DPC	diphenyl carbonate
DPPH	2,2-diphenyl-1-picrylhydrazyl
DPV	differential pulse voltammetry
DRV	dehydration–rehydration vesicle
DS	degree of substitution
DSC	differential scanning calorimetry
DTA	differential thermal analysis
DTG	derivative thermogravimetry
EDTA	ethylenediaminetetraacetic acid
EE	encapsulation/entrapment efficiency
EGMME	ethylene glycol monomethyl ether
EH-DMAB/EHDAB	2-ethylhexyl p-dimethylaminobenzoate/2′-ethylhexyl-4-(N,N-dimethylamino)benzoate
EHMC	ethylhexyl methoxycinnamate (octinoxate)
EPI	epichlorohydrin
EPR	electron paramagnetic resonance
ESI-MS	electrospray ionization mass spectrometry
ESI-UHR-QTOF-MS	electrospray ionization ultrahigh-resolution quadrupole time-of-flight mass spectrometry
ESR	electron spin resonance
Et-β-CD	ethyl-β-cyclodextrin
FA	ferulic acid
FE-SEM	field-emission scanning electron microscopy
FRET	Förster resonance energy transfer
FTIR	Fourier-transform infrared spectroscopy
FT-Raman	Fourier-transform Raman spectroscopy
GC	gas chromatography
GC-FID	gas chromatography with flame-ionization detection
GC-MS	gas chromatography–mass spectrometry
GEM	gemcitabine
GO	graphene oxide
GUMBOS	group of uniform materials based on organic salts
HA	hyaluronic acid
HE-β-CD	hydroxyethyl-β-cyclodextrin
HEC	hydroxyethyl cellulose
HITC	1,1′,3,3,3′,3′-hexamethylindotricarbocyanine iodide
HkβCD	heptakis(2,6-di-O-methyl)-β-cyclodextrin
HMDI	hexamethylene diisocyanate
HP-α-CD	hydroxypropyl-α-cyclodextrin
HP-β-CD	hydroxypropyl-β-cyclodextrin
HP-γ-CD	hydroxypropyl-γ-cyclodextrin
HPCD	hydroxypropyl cyclodextrin; in the lansoprazole study, hydroxypropyl-β-cyclodextrin
HPLC	high-performance liquid chromatography
HPLC-DAD	high-performance liquid chromatography with diode-array detection
HPLC-ESI-MS	high-performance liquid chromatography–electrospray ionization mass spectrometry
HPLC-PDA	high-performance liquid chromatography with photodiode-array detection
HPLC-UV	high-performance liquid chromatography with UV detection
HPMC	hydroxypropyl methylcellulose
HPTLC	high-performance thin-layer chromatography
HRMS	high-resolution mass spectrometry
HRTEM	high-resolution transmission electron microscopy
HSA	human serum albumin
HSQC	heteronuclear single-quantum coherence spectroscopy
HS-SPME-GC-MS	headspace solid-phase microextraction–gas chromatography–mass spectrometry
HTA-β-CD	hydroxytrimethylammoniumpropyl-β-cyclodextrin
ICD	induced circular dichroism
ICG	indocyanine green
ICH	International Council for Harmonisation
IDB	isoamyl 4-(dimethylamino)benzoate
ITC	isothermal titration calorimetry
LA	lactobionic acid
LC-DAD-ESI-MS	liquid chromatography–diode-array detection–electrospray ionization mass spectrometry
LC-MS	liquid chromatography–mass spectrometry
LED	light-emitting diode
LOD	limit of detection
LSP	lansoprazole
MADLS	multi-angle dynamic light scattering
MCA	methoxycinnamic acid
MCC	microcrystalline cellulose
MCPA	4-chloro-2-methylphenoxyacetic acid
MCPP	mecoprop [2-(4-chloro-2-methylphenoxy)propionic acid]
MCR-ALS	multivariate curve resolution–alternating least squares
MD	molecular dynamics
MDA	malondialdehyde
Me-β-CD/M-β-CD/MβCD	methyl-β-cyclodextrin
MIC	minimum inhibitory concentration
MM-PBSA	molecular mechanics/Poisson–Boltzmann surface area
MM2	MM2 molecular-mechanics force field
MMFF94s	Merck Molecular Force Field 94s
MOF	metal–organic framework
MS	molar substitution or, in analytical-method combinations, mass spectrometry
MTT	3-(4,5-dimethylthiazol-2-yl)-2,5-diphenyltetrazolium bromide
MTX	methotrexate
MWCNTs	multi-walled carbon nanotubes
MYR	myricetin
NFOH	hydroxymethylnitrofurazone
NIR	near-infrared
NMR	nuclear magnetic resonance spectroscopy
NOESY	nuclear Overhauser effect spectroscopy
NSAID	nonsteroidal anti-inflammatory drug
O/W	oil-in-water
OMe-β-CD	permethylated β-cyclodextrin
ORAC	oxygen radical absorbance capacity
Ox-CD	thymine-oxetane-modified β-cyclodextrin
PAA	poly(acrylic acid)
PAMAM	poly(amidoamine)
PAS	photoacoustic spectroscopy
PBS	phosphate-buffered saline
PCL	polycaprolactone
PCD	poly(β-cyclodextrin)
PDE-4	phosphodiesterase 4
PDI	polydispersity index
PDT	photodynamic therapy
PEG	polyethylene glycol
PEI	polyethylenimine
PF127	Poloxamer F127
PIMA	poly(isobutylene-alt-maleic anhydride)
PK	pharmacokinetics
PD	pharmacodynamics
PL	photoluminescence
PLSC	pseudorotaxane-like supramolecular complex
PM-α-CD	permethylated α-cyclodextrin
PM-β-CD	permethylated β-cyclodextrin
PMDA	pyromellitic dianhydride
PMF	potential of mean force
PNIPAM	poly(N-isopropylacrylamide)
PPIX	protoporphyrin IX
PPIX-2CD	protoporphyrin IX bearing two cyclodextrin units
PPN	propranolol hydrochloride
PPy	polypyrrole
PTT	photothermal therapy
PVP	polyvinylpyrrolidone
PXRD	powder X-ray diffraction
PZ	promazine
PZQ	praziquantel
QD/QDs	quantum dot/quantum dots
QY	quantum yield
RAMEB	randomly methylated β-cyclodextrin
RES	resveratrol
RGO	reduced graphene oxide
RH	relative humidity
RM-β-CD	randomly methylated β-cyclodextrin
ROESY	rotating-frame Overhauser effect spectroscopy
ROS	reactive oxygen species
RP-HPLC	reversed-phase high-performance liquid chromatography
RSM	response surface methodology
SAAE	star anise alcohol extractives
SACD	succinate-modified β-cyclodextrin
SAED	selected-area electron diffraction
SAXS	small-angle X-ray scattering
SBE-β-CD	sulfobutyl ether-β-cyclodextrin
SBE7βCD	sulfobutyl ether-β-cyclodextrin with an average degree of substitution of approximately 7
SBECD	sulfobutyl ether cyclodextrin; in the manuscript, SBE-β-CD
SCXRD	single-crystal X-ray diffraction
SDS	sodium dodecyl sulfate
SEC	size-exclusion chromatography
SEM	scanning electron microscopy
SH-GC	static-headspace gas chromatography
SLN	solid lipid nanoparticle
SMEDDS	self-microemulsifying drug-delivery system
SNAs	supramolecular nanoassemblies
SNOM	scanning near-field optical microscopy
SPF	sun protection factor
SPME-GC-MS	solid-phase microextraction–gas chromatography–mass spectrometry
STED	stimulated emission depletion
SUF	sufentanil
SVD	singular value decomposition
SWCNTs	single-walled carbon nanotubes
TBARS	thiobarbituric acid-reactive substances
TCSPC	time-correlated single-photon counting
TD-CD	thymine-dimer-modified β-cyclodextrin
TD-DFT	time-dependent density functional theory
TDI	toluene diisocyanate
TEA	triethanolamine
TEM	transmission electron microscopy
TG	thermogravimetry
TGA	thermogravimetric analysis
THPP	5,10,15,20-tetrakis(4-hydroxyphenyl)porphyrin
TICT	twisted intramolecular charge transfer
TMPyP	5,10,15,20-tetrakis(N-methyl-4-pyridyl)porphyrin
TOC	total organic carbon
TPB	tetraphenylborate
TPH	triprolidine hydrochloride
TPP	triphenylphosphine
UCNPs	upconversion nanoparticles
UPLC	ultra-high-performance liquid chromatography
UPLC-DAD	ultra-high-performance liquid chromatography with diode-array detection
UV	ultraviolet
UVA	ultraviolet A
UVA1	long-wave UVA radiation
UVB	ultraviolet B
UVC	ultraviolet C
UV–Vis	ultraviolet–visible spectroscopy
VAP	vitamin A palmitate
VC	valacyclovir
VE	vitamin E
W/O/W	water-in-oil-in-water
XRD	X-ray diffraction
XRPD	X-ray powder diffraction
ZnNPc	zinc naphthalocyanine
ZnPc	zinc phthalocyanine
α-CD	α-cyclodextrin
α-CD-C	coumarin-modified water-soluble α-cyclodextrin
α-TC	α-tocopherol
α-TQ	α-tocopherol
α-TQA	α-tocopheryl acetate
β-CD	β-cyclodextrin
βCDP	β-cyclodextrin polymer
βLAP	β-lapachone
βNS-PYRO	pyromellitic-dianhydride-crosslinked β-cyclodextrin nanosponge
γ-CD	γ-cyclodextrin
s-α-CyD	sulfonated α-cyclodextrin
s-β-CyD	sulfonated β-cyclodextrin
