# Supplementary material for: Cyclodextrins as Modulators of Photostability and Photochemical Behavior: Mechanisms, Analytical Assessment, and Applications
Source: Int J Mol Sci. 2026 Nov 9;27(21):10040. doi: 10.3390/ijms272110040 (PMC13764038; doi:10.3390/ijms272110040)
Supplement: Supplementary file 1 [file ijms-27-10040-s001.zip › ijms-4689746-supplementary.pdf]

Supplementary Materials

Cyclodextrins as Modulators of Photostability and Photochemical Behavior: Mechanisms, Analytical Assessment, and Applications

Monika Zielińska-Pisklak [1,*] , Agnieszka Mykytyn [2], Marcin Gackowski [3] and Łukasz Szeleszczuk [2]

[1] Department of Pharmaceutical Chemistry and Biomaterials, Faculty of Pharmacy, Medical University of Warsaw, 1 Banacha Str., 02-093 Warsaw, Poland; monika.zielinska-pisklak@wum.edu.pl

[2] Department of Organic and Physical Chemistry, Medical University of Warsaw, 1 Banacha Str.,
02-097 Warsaw, Poland; s091821@student.wum.edu.pl (A.M.); lukasz.szeleszczuk@wum.edu.pl (Ł.S.)

[3] Department of Toxicology and Bromatology, Faculty of Pharmacy, L. Rydygier Collegium Medicum in
Bydgoszcz, Nicolaus Copernicus University in Torun, 2 Jurasza Str., 85-089 Bydgoszcz, Poland;
marcin.gackowski@cm.umk.pl

* Correspondence: monika.zielinska-pisklak@wum.edu.pl

Table S1. Previous reviews and perspective articles addressing cyclodextrin-mediated photostability and related light-responsive applications.

Review scope	Cyclodextrin systems	Main light-related focus and conclusions	Ref
Core reviews of pharmaceutical photostability and cyclodextrin-mediated stabilization			
**Pharmaceutical photostability and stabilization strategies**	Native and modified CDs; inclusion and CD-liposome systems	CD complexation is discussed among several photostabilization strategies; effects depend on the drug, excipients and formulation context	[1]
**Drug photodegradation and photostability testing**	α-, β-, γ-CDs and modified derivatives	Covers drug photochemistry, photodegradation kinetics and ICH photostability testing, with examples of CD-mediated photoprotection	[2]
**Photostability of pharmaceutical dosage forms**	α-, β-, γ-CDs and derivatives	Summarizes photodegradation mechanisms and formulation factors, with CDs included among established approaches to improve photostability	[35]
**Topical formulations of light-sensitive drugs**	Native and modified CDs; CD matrices and CD-vesicle systems	Reviews topical photostability methods and CD-based systems used to reduce photodegradation while supporting drug delivery	[3]

Review scope	Cyclodextrin systems	Main light-related focus and conclusions	Ref
**Photoprotection by cyclodextrins and liposomes**	Native and modified α-, β- and γ-CDs; CD and liposomal systems	Focused review of CD- and liposome-based photoprotection, including combined systems that can further improve photostability	[36]
**Systematic comparison of photostabilization strategies**	Native and modified CDs; several encapsulation strategies	CDs were the most frequent encapsulation strategy (13/40), but photostabilization outcomes and experimental conditions were highly heterogeneous	[42]
**Photostability of 1,4-dihydropyridines**	β-, γ- and modified α/β/γ-CDs; combined CD–polymer and CD–liposome systems	Photostability varies with CD identity and drug-host compatibility; some systems are ineffective or destabilizing, whereas combined carriers can enhance protection	[37]
**Cyclodextrin effects on drug stability**	Native and modified CDs; binary and multicomponent systems	CDs can increase or decrease drug stability, including photostability, depending on CD type, inclusion-complex structure and multicomponent composition	[38]
Sunscreens, UV filters and topical/cosmetic photoprotection			
**Photostability of organic UV filters**	β-CD, HP-β-CD and other CDs; free and formulated UV-filter systems	CD inclusion can modify UV-filter photostability, with outcomes depending on filter identity and formulation conditions	[9]
**Avobenzone photostabilization and delivery**	β-CD and derivatives; HP-β-CD highlighted	CDs are discussed as an important avobenzone photostabilization strategy, with selected systems improving photostability and limiting skin penetration	[204]
**Sunscreen technologies and cutaneous photoprotection**	Cyclodextrins among nanocarrier and encapsulation technologies	CDs are briefly discussed as sunscreen nanocarriers; their role in CD-specific photostability is only indirectly addressed	[205]
**Cyclodextrin-based topical and dermocosmetic delivery**	Native/modified and amphiphilic CDs, nanosponges and CD-MOFs	Selected topical CD systems improve photostability, considered together with solubility, release, skin penetration and safety	[206]
Broad cyclodextrin reviews with photostability coverage			
**Cyclodextrin inclusion complexes: preparation and applications**	Native and modified α-, β- and γ-CDs	Photostability is presented as one of several properties that can be improved by CD inclusion, illustrated through selected examples	[207]

Review scope	Cyclodextrin systems	Main light-related focus and conclusions	Ref
**Fat-soluble vitamin - cyclodextrin systems**	Native/modified and functionalized CDs, nanosponges and CD-containing materials	Selected vitamin studies show that CD systems can improve photostability or limit photoisomerization, depending on vitamer, CD type and formulation	[208]
Photoactive, photodynamic and supramolecular photochemistry reviews			
**Excited-state processes in cyclodextrin nanocavities**	α-, β-, γ-, DM-β- and HP-γ-CD host–guest complexes	CD nanoconfinement alters excited-state relaxation, transfer, twisting and isomerization in ways relevant to photostability, although protective efficacy is not the main focus	[39]
**Cyclodextrin-based photoactive and photodynamic systems**	Natural/modified and amphiphilic CDs, CD conjugates and photoresponsive assemblies	CD-based photoactive systems can support photostability while preserving photochemical function and limiting aggregation or self-quenching	[40]
**Porphyrin-based antimicrobial photodynamic therapy**	Captisol (SBE-β-CD) highlighted in one CD-based system	CD-specific coverage is limited to a Captisol/TMPyP system showing improved photostability with retained antimicrobial photodynamic activity	[209]
**Supramolecular dye assemblies for imaging and phototherapy**	α-, β-, γ-CDs and modified/targeted CD derivatives and polymers	CD host-guest systems can improve dye photostability, reduce quenching and support sustained imaging and phototherapeutic function	[210]

Table S2. Studies reporting absent, adverse, marginal or strongly condition-dependent effects of cyclodextrins on photostability

Guest / application	CD system	Irradiation / assessment	Photostability outcome	Interpretation	Ref
No meaningful or marginal photoprotection					
**Brilliant Blue FCF - Triarylmethane food/cosmetic dye**	β-CD; 1:1; Co-evaporated inclusion complex	Sunlight, 3 h; UV-Vis at 630 nm	Degradation was 0.135% free vs 0.086% complexed after 3 h; the absolute difference was only 0.049 percentage points	The absolute difference was negligible; both free and complexed dye were essentially stable over the short exposure	[211]

Guest / application	CD system	Irradiation / assessment	Photostability outcome	Interpretation	Ref
**Carprofen - NSAID**	HP-β-CD; 1:1; K_S = 487 M^{-1}; Freeze-dried inclusion complex in 20% ethanol/water	Fluorescent light, 1500 lx, 30 cm; HPLC first-order kinetics	k = 0.113 ± 0.003 h^{-1} free vs 0.126 ± 0.017 h^{-1} complexed; no significant photostabilization	Matched HPLC kinetics showed no significant benefit despite confirmed HP-β-CD association	[113]
**Curcumin - Natural photosensitizer for antimicrobial PDT**	Methyl-β-CD (MβCD); 1:1 complex indicated at lower MβCD concentrations; 1:1 and 1:3 preparation ratios; Freeze-dried amorphous solid dispersions containing MβCD and HA, HPMC or HA/HPMC	Suntest CPS+ xenon, λ > ~310 nm, 765 W m^{-2} to 1.2 × 10^6 lux·h (ICH Q1B Option 1); HPLC	No photoprotection: all lyophilizates were almost completely bleached; residual curcumin was 0.1 ± 0.1% w/w versus initial loads of 1.4-5.5% w/w	The MβCD-containing solid dispersions did not protect curcumin under the ICH-type exposure; additional polymers did not prevent extensive bleaching	[164]
**Diloxanide furoate - Luminal amoebicide**	β-CD, HP-β-CD; Not established; 0.5% w/v CD; Aqueous–acetonitrile DF solutions containing CD	UVC 254 nm, 3 cm; stability-indicating HPLC and pseudo-first-order kinetics	β-CD, HP-β-CD and control gave essentially the same $t_{1/2}$ (6.5 ± 0.4 min); neither CD provided photoprotection	Stability-indicating HPLC confirmed no CD-specific effect, although other photoprotective agents were effective in separate tests	[212]
**Ensulizole (2-phenylbenzimidazole-5-sulfonic acid, PBS) - Organic UVB sunscreen filter**	Me-β-CD; 1:1; K = 155.4 M^{-1}; Freeze-dried PBS/Me-β-CD inclusion complex	Solar-light exposure, up to 4 h; UV-Vis	Free PBS already showed no change in spectral maxima over 4 h and only slight absorbance variation; the complex showed essentially no spectral change, so the incremental benefit was marginal	The free filter was already highly photostable, leaving little room for an incremental CD benefit	[213]

Guest / application	CD system	Irradiation / assessment	Photostability outcome	Interpretation	Ref
**Hypericin - Photosensitizer for PDT**	β-CD; 1:2 HY:β-CD sandwich model; complex prepared from equimolar components; Lyophilized β-CD/HY complex; DMSO solution for photostability test	590 ± 10 nm LED, 15.8 mW cm^{-2}, 3 h, continuous stirring/O_2; UV-Vis	After 3 h, ≈83% complexed vs ≈88% free HY remained; complexation did not improve photostability and largely preserved HY photochemical properties	β-CD preserved the intended photochemical behavior of the photosensitizer but did not increase resistance to bleaching	[214]
**Ofloxacin - Fluoroquinolone antibacterial drug**	β-CD; 1:1; K_C = 152 M^{-1}; 1:1 and 1:3 products tested; Freeze-dried inclusion complexes	UVA 320-400 nm, 125 W, 21 days; HPLC-DAD	Only 3% (1:1) and 6% (1:3) ofloxacin remained after 21 d; photoproduct profiles resembled free drug. Partial inclusion did not protect the N-methylpiperazinyl moiety	Complex formation was insufficient because the photolabile N-methylpiperazinyl region remained inadequately protected	[114]
**Oxybenzone - Organic UVA/UVB sunscreen filter**	SBE-β-CD; HP-β-CD and hydroxypropyl α-/γ-CDs screened; 1:1 solution complex; 1:2 guest:host co-evaporate used in subsequent studies; Oxybenzone/SBE-β-CD complex in 30% ethanol/water	Suntest CPS+, λ > 290 nm, 500 W m^{-2} for 2 h; HPLC	No significant effect: degradation was 2.3 ± 2.2% free vs 2.9 ± 2.7% with SBE-β-CD ($p > 0.4$)	SBE-β-CD altered formulation/skin-distribution properties but did not measurably improve chemical photostability under the tested conditions	[177]

Guest / application	CD system	Irradiation / assessment	Photostability outcome	Interpretation	Ref
**Tretinoin - Topical anti-acne retinoid**	β-CD; Not established; β-CD 1.5% w/v; Tretinoin lotion with β-CD added directly	450 W xenon lamp, 28 cm, ≤36°C; HPLC of tretinoin and photoisomers	≈20% tretinoin remained after 30 min; 1.5% β-CD did not alter photodegradation. Yellow dyes, not β-CD, retarded loss	Direct addition of β-CD to the lotion did not slow degradation; protection was instead observed with yellow-dye comparators	[215]
**Triprolidine hydrochloride, E-isomer - Antihistamine; photosensitive drug**	β-CD; 1:1 by mass physical mixture; Solid-state TPH/β-CD physical mixture	Suntest CPS+, 550 Wh m^{-2}, 40°C, 20 h (ICH); HPLC; dark control	Only ≈10% TPH degraded; β-CD did not significantly change the limited photodegradation or E→Z isomerization	Baseline degradation was small and β-CD produced no significant incremental change in parent loss or photoisomerization	[216]
**Valacyclovir hydrochloride - Antiviral prodrug used against HSV infections**	γ-CD; 1:1; K_a≈ 3149.9 M^{-1}; Co-evaporated VC/γ-CD inclusion complex; Carbopol 940 gel evaluated separately	Aqueous VC and complex exposed to sunlight for 3 h; UV-Vis at 252 nm	Only a marginal difference: 0.087% degradation free vs 0.072% complexed after 3 h; both samples were essentially photostable	Both free and complexed valacyclovir were essentially photostable; the observed difference was too small to represent meaningful protection	[217]
Condition-dependent or cyclodextrin-dependent effects					
**Avobenzone - Organic UVA sunscreen filter**	β-CD; CB[7] comparator; 1:1 complex; AVO:β-CD = 1:50 or 1:100; Hydroalcoholic host-guest solutions	UV irradiation, 60 min; 10% or 30% ethanol/water; HPLC-UV and UV-Vis	At 10% ethanol, recovery increased from 6 ± 2% free to 33 ± 2% (1:50) and 56 ± 6% (1:100); at 30% ethanol, free AVO already retained 88 ± 9% and β-CD gave no significant additional protection	The apparent CD benefit depended strongly on solvent composition because ethanol changed the intrinsic stability of free avobenzone	[59]
**Benzothiazole- and indolinium-derived cyanine dyes 2–9 - Fluorescent dyes for imaging, diagnostics and optical applications**	2,6-Dimethyl-β-CD; Not determined; In situ aqueous dye–CD systems containing 1-3% DMSO	Ambient light at 25°C; absorbance monitored over several days	Strongly structure-dependent: dyes 2-5 were not protected; dye 6 was stable for ≥5 d vs complete bleaching in 2 days free, and dye 7 retained ~60% after 4 days vs complete bleaching in 3 days. Dyes 8–9 showed little or no CD effect	The same host produced protection, no effect or little effect depending on dye structure	[218]

Guest / application	CD system	Irradiation / assessment	Photostability outcome	Interpretation	Ref
**Curcumin and five symmetric/asymmetric curcuminoids - Natural and synthetic antioxidant/pigment compounds**	HP-β-CD (photostability); M-β-CD and HP-γ-CD evaluated in other experiments; Not directly determined; Curcuminoids in 10% aqueous HP-β-CD at pH 5	Xenon Suntest, λ > ~310 nm, 550 W m^{-2} at 30°C; HPLC	Structure-dependent: HP-β-CD increased $t_{1/2}$ of C-1-C-3 versus buffer/MeOH, but reduced curcumin (C-4) $t_{1/2}$ from 12.8 to 7.8 min, C-5 from 4.43 to 2.74 min and C-6 from ~74 to ~40 min	Closely related curcuminoids responded in opposite directions to the same HP-β-CD environment	[26]
**EHMC, avobenzone (DBM) and curcumin - UV filters and natural photoactive bioactive**	Epichlorohydrin-crosslinked γ-CD polymer (pyCD); 1:1 guest: γ-CD unit; Water-soluble pyCD inclusion complexes	UVB (EHMC) or UVA (DBM/curcumin), 9 W, 60 min; water or ethylene glycol; UV-Vis	EHMC was stabilized (85% in water; 93% in ethylene glycol), whereas DBM/curcumin were not consistently protected; DBM retention in water increased from ≈38% to ≈65% under N_2	Guest identity, solvent and oxygen status jointly determined whether the γ-CD polymer was protective	[176]
**EHMC, avobenzone (DBM) and curcumin - UV filters and natural photoactive bioactive**	β-CD polymers: pbCDE (epichlorohydrin) and pbCDC (citric acid); 1:1 guest:β-CD unit; Water-soluble polymer inclusion complexes	UVB (EHMC) or UVA (DBM/curcumin), 9 W, 60 min; water or ethylene glycol; UV-Vis	pbCDE improved all three guests; EHMC reached 84% (water) and 93% (ethylene glycol). pbCDC gave little benefit for EHMC and decreased DBM/curcumin stability; DBM-pbCDC fell to ≈55% in water	Changing crosslinker chemistry reversed the outcome for the same guests, with solvent further modulating the effect	[61]
**Modified 1,4-dihydropyridine derivatives - Photosensitive DHP analogues**	β-CD; Liquid-phase inclusion complexes	UVC 254 nm, 30 cm; 1000-2900 min; UV-Vis kinetics	Effect depended on substituent: β-CD protected nitro derivatives; 2-NO_2-NR complex degraded ≈200× more slowly than crystalline drug, whereas halogen- and cyano derivatives photodegraded ≈4× faster with β-CD	Substituent pattern reversed the β-CD effect, from strong protection to accelerated degradation	[58]

Guest / application	CD system	Irradiation / assessment	Photostability outcome	Interpretation	Ref
**Praziquantel - Anthelmintic drug**	HP-β-CD, Me-β-CD; 1:1 equimolar co-ground products; Amorphous CD complexes prepared by neat grinding	ICH photostability: 1.2 million lux·h + 200 Wh m^{-2}, 1 week; HPLC	Pure PZQ and PZQ/HP-β-CD showed no significant photodegradation; PZQ/Me-β-CD lost ≈4.3%. Malic-acid-containing systems lost up to ≈6.8-7.9%	The outcome depended on CD identity and ternary formulation composition; the free drug was already relatively stable	[219]
**Quercetin - Natural flavonoid antioxidant**	Me-β-CD; 1:2 preparation ratio; K ≈ 434 M^{-1}; Freeze-dried inclusion complex; hydroalcoholic solution	UVA 320–400 nm, ≈6.0 × 10^{-4} W cm^{-2}, 10 cm, up to 120 min; UV-Vis	Only a modest reduction in photodegradation; complexed Q degraded slightly more slowly than free Q in 15% ethanol, but faster in water	The direction and magnitude of the Me-β-CD effect depended on solvent composition	[220]
**Rac-nicardipine - Chiral 1,4-dihydropyridine calcium-channel blocker**	β-CD and HP-β-CD; 1:1; K_C ≈ 500 M^{-1} (β-CD) and 316 M^{-1} (HP-β-CD); In situ aqueous inclusion systems at pH 7.4 and 5.0	150 W xenon-arc solar simulator, UVA-UVB; achiral and enantioselective CE, DAD and GC-MS	β-CD reduced overall photodegradation, whereas HP-β-CD accelerated it. Both hosts induced stereoselective degradation with opposite enantiomer profiles	β-CD and HP-β-CD produced opposite effects and altered stereoselective degradation profiles	[221]
**Rac-nicardipine hydrochloride - Photolabile 1,4-dihydropyridine calcium-channel blocker**	α-CD, β-CD, γ-CD, HP-α-CD, HP-β-CD, HP-γ-CD, HE-β-CD and M-β-CD; 1:1; Equimolar co-evaporated solid systems subsequently dissolved in water	150 W xenon-arc solar simulator, UVA-UVB; chiral capillary electrophoresis	β-CD, HP-α-CD and HE-β-CD were photoprotective; α-CD accelerated degradation; γ-CD, HP-β-CD, HP-γ-CD and M-β-CD had no significant effect. β-CD also produced different rates for the two enantiomers	Different native and substituted CDs produced protective, neutral or adverse responses for the same drug	[27]

Guest / application	CD system	Irradiation / assessment	Photostability outcome	Interpretation	Ref
**Tenoxicam - NSAID and analgesic oxicam**	β-CD; TXM:β-CD = 1:1, 1:5 and 1:10; In situ aqueous complexes at pH 7.5	Suntest CPS xenon, 765 W m^{-2}; validated LC-PDA kinetics; dark controls	Concentration-dependent effect: β-CD modestly retarded tenoxicam photodegradation at low initial drug concentrations but accelerated the process at higher concentrations. NMR evidence of drug self-association suggests a contribution from concentration-dependent monomer–dimer equilibria.	Drug concentration and self-association shifted β-CD from slight protection to accelerated photodegradation	[29]
**5,10,15,20-Tetrakis(4-hydroxyphenyl)porphyrin (THPP) - Photosensitizer for antimicrobial PDT of infected wounds**	Methyl-β-CD, heptakis(2,6-di-O-methyl)-β-CD and HP-β-CD; Not determined; THPP:PF127: CD = 1:1:30 or 1:1:10; THPP/PF127/CD systems in ionically cross-linked dry alginate foams	Suntest CPS+ xenon, 310–800 nm, 765 W m^{-2} for 1 and 3 h; HPLC and color measurements	After 3 h, THPP remaining was 17.0% without CD, 9.6% with MβCD, 20.3% with HkβCD and 24.4% with high HP-β-CD; effects ranged from destabilization to modest protection	CD identity and concentration within the multicomponent foam determined whether the photosensitizer was destabilized or modestly protected	[222]
**Zinc naphthalocyanine and substituted/unsubstituted zinc phthalocyanines - Photosensitizers for PDT and photoactive materials**	β-CD; HP-γ-CD for tetra-tert-butylphenoxy-ZnPc; Mainly 2:1 CD:photosensitizer; 4:1 also observed; DMSO-prepared inclusion complexes; selected co-ground systems	Q-band irradiation near 700 nm with a 300 W lamp; photobleaching, fluorescence and $^1O_2$ quantum yields	Mixed effect: φp decreased for ZnPc ($2.61 \rightarrow 0.68 \times 10^{-5}$) and tetra-tert-butylphenoxy-ZnPc ($3.33 \rightarrow 2.75 \times 10^{-5}$), was unchanged for two derivatives, but increased for ZnNPc ($16.35 \rightarrow 20.4 \times 10^{-5}$)	The effect on photobleaching depended on photosensitizer structure and was not uniform across the series	[223]
Adverse effects: accelerated photodegradation or altered pathways					

Guest / application	CD system	Irradiation / assessment	Photostability outcome	Interpretation	Ref
**2-(2-Nitrovinyl)furan (G-0) - Antibacterial, antifungal and antiprotozoal drug candidate**	HP-β-CD (Trappsol®) and SBE-β-CD (Captisol®); 1:1 preparation ratio; Freeze-dried inclusion complexes; corresponding physical mixtures as controls	Forced 254 nm irradiation and ICH Q1B Option 2 exposure (1.2×10^6 lux·h; 200 Wh m^{-2} UVA); HPLC-DAD	No photoprotective advantage: $t_{1/2}$ was 23.90 h (HP-β-CD complex) and 24.93 h (SBE-β-CD complex) vs 25.23 and 37.90 h for the respective physical mixtures; complexation changed kinetics and photoproduct profiles	Inclusion complexes offered no advantage over corresponding physical mixtures and altered degradation kinetics/products	[224]
**β-Lapachone - Anticancer naphthoquinone**	RM-β-CD (MS 0.57); Not specified; 5% w/v RM-β-CD; Aqueous β-lapachone/RM-β-CD system	1800 lx + 370 nm UV, 0.0078 W m^{-2}, 4 °C; HPLC; dark control	RM-β-CD protected in darkness ($t_{1/2}$ 1386 vs 330 days) but strongly accelerated photolysis under light ($t_{1/2}$ 0.5 vs 2.5 days)	RM-β-CD strongly improved dark stability but accelerated photolysis, demonstrating pathway-specific reversal of the stability effect	[28]
**Curcumin - Natural pigment, antioxidant and potential therapeutic photosensitizer**	HP-α-CD, HP-β-CD, HP-γ-CD, RM-β-CD, SBE-β-CD and HTA-β-CD; Predominantly 1:1 from phase-solubility behavior; In situ aqueous systems containing 5% CD in phosphate buffer, pH 5	Suntest CPS xenon, $\lambda > \sim 310$ nm; HPLC first-order kinetics corrected for absorptivity	All CD systems accelerated photodegradation relative to organic comparators: $t_{1/2}$ = 20.4-46.2 min with CDs vs 70.7 min in 40% ethanol/buffer and 133.3 min in methanol	All tested CDs accelerated photodegradation under irradiation despite favorable complexation and non-photochemical stabilization	[50]

Guest / application	CD system	Irradiation / assessment	Photostability outcome	Interpretation	Ref
**Eleven organophosphorus pesticides - Agricultural pesticides; aquatic contaminants**	α-CD, β-CD, γ-CD; Not established; CD concentration 8 mM; Pesticide-CD systems in humic water	Artificial sunlight, 25°C; GC-FID pseudo-first-order kinetics; ESR of humic radicals	All CDs accelerated photodegradation; promotion generally followed α-CD < γ-CD < β-CD and reached +45.8% for chlorpyrifos with β-CD in HA-F	In humic water, all CDs promoted photodegradation; the environmental photosensitizing matrix reversed the expected protective role	[195]
**Ketoprofen - NSAID associated with topical photosensitivity**	β-CD; Not established; reported K ≈ 810 M^{-1}; Aqueous KP/β-CD systems at pH 5.0 (0.5 or 8 mM β-CD)	UVC 253.7 nm, 15 W, 46.5 cm, 0-48 h; RP-HPLC, UV-Vis and SVD trajectory analysis	β-CD did not protect ketoprofen; it changed the multistep photolysis pathway and promoted formation of a late hydrophilic product	β-CD did not protect the parent drug and redirected the multistep photolysis pathway toward an additional product	[115]
**Midazolam — Benzodiazepine sedative/anxiolytic**	RM-β-CD; 1:1 (ROESY); higher-order species possible; Aqueous inclusion system in phosphate buffer, pH 5.0	Suntest CPS+ xenon/Solar ID65, 550 W m^{-2}, 40 °C, 12 h (1.2 million lux·h); HPLC-UV, LC–MS/MS; dark control	Photodegradation was similar but slightly faster with RM-β-CD; complexation altered the pathway and generated additional intermediate and dimer photoproducts	RM-β-CD slightly accelerated loss and generated additional intermediate/dimer photoproducts.	[69]
**Praziquantel - BCS II anthelmintic drug used against schistosomiasis**	β-CD, HP-β-CD, RM-β-CD and SBE-β-CD; 1:1; Mechanochemically prepared solid PZQ/CD complexes	ATLAS CPS+ xenon/ID65: 300 W m^{-2} for 1 h and 700 W m^{-2} for 8 h; UPLC-DAD/MS and HRMS/MS	No uniform protection: most PZQ/CD complexes remained stable, but PZQ/SBE-β-CD retained only 75.12% PZQ peak area after 8 h at 700 W m^{-2}; complexation also introduced new degradation pathways	Protection was not uniform across hosts; SBE-β-CD showed substantial loss at high exposure and new degradation pathways appeared	[225]

Guest / application	CD system	Irradiation / assessment	Photostability outcome	Interpretation	Ref
**Rhein - Anthraquinone anti-inflammatory bioactive**	HP-β-CD; β-CD screened in phase-solubility studies; 1:1; K_c ≈ 253 M^{-1} for HP-β-CD; Aqueous Rhein/HP-β-CD systems in phosphate buffer, pH 7.2	Visible light, 800 lx, 10 cm, 25°C, up to 9 days; UV-Vis/fluorescence; dark control	HP-β-CD decreased Rhein photostability in a concentration-dependent manner (1-25 mM CD)	HP-β-CD decreased photostability progressively with increasing CD concentration	[226]

Table S3. Studies providing indirect, formulation-level, computational or otherwise limited evidence of cyclodextrin-mediated photoprotection

Guest / application	CD system / formulation	Light-related assessment	Reported finding	Main evidential limitation	Ref
Formulation-level or functional evidence					
**α-Tocopherol (vitamin E) - Antioxidant vitamin for food and pharmaceutical applications**	HP-β-CD; 1:1 and 1:2 feed ratios; final nanofibers ≈0.80:2.00 vitamin E:HP-β-CD by ^{1}H NMR; Polymer-free electrospun vitamin E/HP-β-CD inclusion-complex nanofibers	300 W UV lamp, 30 cm, 120 min; post-irradiation DPPH assay	UV-exposed nanofibers retained DPPH-scavenging activity, although the reaction slowed from ~35 s to >90 s; intact vitamin E was not quantified	Functional endpoint only: antioxidant activity was measured, but intact vitamin E was not quantified after irradiation	[165]
**Azoxystrobin - Broad-spectrum strobilurin fungicide**	β-CD; β-CD:AZO preparation ratio 1.1:1; AZO/β-CD inclusion system in a Ca^{2+} -crosslinked alkali-lignin/sodium-alginate hydrogel	254 nm UV, 30 W, 25 cm, up to 120 h; residual AZO by UV-Vis	After 120 h, 57.37% AZO remained in the composite vs 7.67% in commercial AZO; the CD contribution was not separated from lignin absorption and hydrogel shielding	Formulation confounding: β-CD could not be separated from lignin UV absorption and hydrogel confinement	[196]

Guest / application	CD system / formulation	Light-related assessment	Reported finding	Main evidential limitation	Ref
**Benzophenone-3 (oxybenzone) - Broad-spectrum UVA/UVB sunscreen filter**	HP-β-CD; 1:1 from phase solubility, K ≈ 5398 M^{-1}; 1:1 and 1:2 solids prepared; BZ-3/HP-β-CD complex incorporated into an O/W sunscreen formulation	No photodegradation test; PAS (240-500 nm) assessed optical absorption and ex vivo skin penetration	The complex retained UV absorption and reduced BZ-3 penetration; the 1:2 formulation gave the lowest dermal penetration. Photostability was not quantified	No photodegradation experiment; optical absorption and skin penetration do not establish chemical photostability	[227]
**β-Carotene - Carotenoid antioxidant and nutraceutical**	HP-β-CD; HP-γ-CD; β-Carotene:CD feed ratio 1:6; phase-solubility profiles consistent with 1:1 association; Polymer-free electrospun β-carotene/CD inclusion-complex nanofibers	300 W UV lamp, 15 cm, 6 h; photostability inferred from post-irradiation DPPH activity	Antioxidant activity fell strongly for free β-carotene, less for HP-β-CD nanofibers and least for HP-γ-CD nanofibers; intact β-carotene was not quantified	Functional endpoint only: DPPH activity was used as a surrogate and intact β-carotene was not quantified	[228]
**Hydroquinone - Topical depigmenting drug for melasma**	HP-β-CD; HQ:HP-β-CD = 3:1; inclusion efficiency ≈98%; HQ/HP-β-CD complex incorporated into a perilla-oil O/W cream	Storage at 25°C for 90 days; HPLC drug content and color monitoring; no defined irradiation protocol	At day 90, ~90% HQ remained without browning in HQ/HP-β-CD/perilla-oil cream vs ~18% in free-HQ cream; CD-specific protection was confounded by the formulation matrix	No defined irradiation protocol and strong formulation confounding from perilla oil, TiO_2, bisulfite and the emulsion matrix	[229]
**Onion peel extract (quercetin- and resveratrol-rich) - Phenolic extract investigated as a natural cosmetic UV filter**	β-CD; Not defined for the multicomponent extract; β-CD microparticles incorporated into O/W sunscreen formulations	SPF at weeks 0, 2 and 4 during a 5-week stability study; 1 h sunlight skin-reactivity test in one volunteer at week 4; no isolated-guest photolysis	MP-OPE SPF decreased from 18.2 ± 0.5 to 6.3 ± 0.0, vs 4.7 ± 0.1 to 1.4 ± 0.4 for free OPE; the CD-specific chemical protection was not isolated	Formulation/SPF study without isolated-guest photolysis; CD-specific chemical protection could not be separated	[230]

Guest / application	CD system / formulation	Light-related assessment	Reported finding	Main evidential limitation	Ref
**Photosensitive sulfonamide model drug - Model pharmaceutical substrate for photostability testing**	RAMEB (RM-β-CD), used to solubilize BMD and MBC; No drug:CD complex; UV-filter:RAMEB stoichiometry not fixed; Aqueous sulfonamide solution containing RAMEB-solubilized BMD or MBC	Sunlight, up to 48 h; HPLC of parent drug and four degradants	RAMEB alone and MBC/RAMEB did not alter photolysis; BMD/RAMEB increased $t_{1/2}$ from 4 to 35 h. Protection was UV-filter-mediated, not direct drug-CD shielding	Protection arose from a RAMEB-solubilized UV filter rather than direct drug-CD complexation	[231]
**Quercetin - Natural flavonoid antioxidant and nutraceutical**	β-CD; 1:1 (phase solubility); K_c = 3345 M^{-1}; β-CD/quercetin inclusion complex in crosslinked PAA film and electrospun nanofibers	366 nm UV, 8 W, 10 cm, 210 min; released quercetin quantified by UV-Vis after irradiation	75.46 ± 0.13% (film) and 74.40 ± 0.06% (nanofibers) remained relative to non-irradiated formulations; no irradiated free-quercetin control	No matched irradiated free-quercetin control under the same protocol, preventing quantification of the CD-specific effect	[197]
**Resveratrol - Natural antioxidant and nutraceutical**	γ-CD-based metal-organic framework (CD-MOF); No discrete host–guest stoichiometry; nanoparticle loading system; Resveratrol-loaded γ-CD-MOF/chitosan core-shell nanocapsules	UV irradiation, 20 W, 30 min; functional stability assessed by DPPH	Free resveratrol showed a marked post-UV fall in DPPH activity, whereas Res-CD-MOF/CS remained highly reactive; intact resveratrol was not quantified, and the chitosan contribution was not isolated	Functional DPPH endpoint plus chitosan/MOF confounding; intact resveratrol was not quantified	[198]
Limited direct photostability evidence					
**3-(1-Naphthyl)-D-alanine hydrochloride (D-1NA) - Synthetic amino-acid derivative for sensing and delivery studies**	β-CD; 1:1; K_a = 5080 M^{-1} at 298.15 K; Solid inclusion complex prepared from 10% aqueous ethanol and reconstituted for analysis	UV irradiation for 225 min; UV–Vis every 45 min; wavelength and intensity not reported	Reported degradation decreased from 13.05% free to 4.83% complexed; no chromatographic confirmation or well-characterized irradiation source	Irradiation wavelength/intensity were not reported and degradation was assessed by UV-Vis without chromatographic confirmation.	[232]

Guest / application	CD system / formulation	Light-related assessment	Reported finding	Main evidential limitation	Ref
**7,3′,4′-Trihydroxyisoflavone (734THIF) Cosmetic antioxidant and antipollutant isoflavone**	HP-β-CD; No discrete stoichiometry established; 5:1 HP-β-CD:734THIF preparation ratio; Co-evaporated/freeze-dried 5-7HP inclusion complex tested as solution and powder	Outdoor daylight, up to 168 h; residual 734THIF by HPLC	After 168 h, 71.49 ± 3.31% remained in solution and 85.52 ± 1.13% in powder; raw 734THIF was not quantitatively reported under the same protocol	No quantitatively matched irradiated free-guest control under the same daylight protocol	[233]
**Black-rice anthocyanin extract (predominantly cyanidin-3-glucoside) - Natural colorant and antioxidant food bioactive**	Cycloamylose, DP 6–31; β-CD comparator; Not determined for the extract; Lyophilized BRA/cycloamylose and BRA/β-CD systems	G8T5 UVA lamp, up to 24 h at pH 2–8; residual antioxidant activity by ABTS; irradiance not reported	At pH 4, antioxidant-activity $t_{1/2}$ increased from 40.38 h free to 58.54 h with 10% cycloamylose and 62.94 h with 5% β-CD; endpoint was functional, not intact anthocyanin	The endpoint was antioxidant activity rather than intact anthocyanin; irradiance was not reported and cycloamylose was the primary system.	[234]
**Gemcitabine - Anticancer nucleoside analogue**	β-CD; 1:1; $K_a = 19.4 \times 10^3$ M^{-1}; Coprecipitated solid inclusion complex; aqueous solutions used for irradiation	Sunlight for >5 h; UV-Vis at 268 nm every 45 min; irradiance and spectrum not characterized	Reported degradation was 13.0% free vs 4.0% for GEM/β-CD; assessment relied on UV-Vis without chromatographic photoproduct analysis	Sunlight exposure was not characterized, and parent loss was inferred from UV-Vis without chromatographic confirmation	[235]
**Hop β-acids mixture - Hop-derived antimicrobial and brewing bioactives**	HP-β-CD, DS = 5.0; Not determined; preparation ratio 18.4 mg β-acids:400 mg HP-β-CD; Co-ground water-soluble β-acids/HP-β-CD inclusion product	Natural light, darkness and 30 W UV for 3 h/day over 120 h; UV-Vis at 356 nm	>95% β-acids were retained under natural light/darkness and >85% after 15 h cumulative UV; no irradiated free β-acids control was included	No irradiated free β-acids control was included, so the magnitude of a CD-specific benefit cannot be established	[236]

Guest / application	CD system / formulation	Light-related assessment	Reported finding	Main evidential limitation	Ref
**Rhodanine - Bioactive heterocycle used as a host–guest model compound**	β-CD; α-CD; 1:1; K_a = 8.95 × 10^3 M^{-1} (β-CD) and 10.02 × 10^3 M^{-1} (α-CD); Coprecipitated solid inclusion complexes; aqueous solutions used for irradiation	Sunlight; UV-Vis at 297 nm every 45 min; irradiation dose not characterized	Reported degradation was 4.00% free, 1.30% with β-CD and 0.70% with α-CD; absolute loss was small and no chromatographic assay was used	Small absolute degradation, uncharacterized sunlight exposure and a UV-Vis-only endpoint limit confidence in the apparent protection	[237]
Computational evidence only					
**Avobenzone (keto and enol forms); octocrylene - Organic UVA/UVB sunscreen filters**	β-CD; HP-β-CD, DS = 1; 1:1 computational complexes; Noncovalent host-guest complexes simulated in explicit water	No irradiation experiment; all-atom explicit-water MD (0.6 μs) and LIE binding-free-energy analysis	Stable complexes formed spontaneously with reduced solvent exposure and guest mobility; octocrylene/β-CD was the most stable model. No experimental photoprotection was demonstrated	Computational evidence only: no irradiation experiment or experimental photostability endpoint was performed	[204]
**4-Methoxy-, 2,4,5-trimethoxy- and 2,4,6-trimethoxycinnamic acids - Model cinnamate UV filters**	α-CD; β-CD; γ-CD; 1:1 computational complexes; Five in silico MCA/CD inclusion complexes in two guest orientations	No irradiation experiment; DFT and TD-DFT calculations of structure, energetics and UV-Vis transitions	Orientation B was favored; 245-MCA/β-CD had the most negative aqueous binding energy (ΔEb = −25.58 kcal mol^{-1}), and calculated spectra retained UVA/UVB absorption	Computational evidence only: predicted inclusion energetics/electronic structure were not tested in an irradiation experiment	[74]

Table S4. Cyclodextrin-based photoactive and photoresponsive systems beyond conventional photoprotection.

Photoactive component / guest	Application	Cyclodextrin system / architecture	Role of CD	Light-related assessment	Main photoactive / photoresponsive outcome	Ref.
Photoresponsive host-guest switching and light-controlled release						

Photoactive component / guest	Application	Cyclodextrin system / architecture	Role of CD	Light-related assessment	Main photoactive / photoresponsive outcome	Ref.
**Azobenzene residues in Az-PAH**	Photoresponsive polymer film / optical switching	Sulfonated α-CD (s-α-CyD); sulfonated β-CD (s-β-CyD); inclusion observed for Az-PAH/s-α-CyD but not Az-PAH/s-β-CyD; Layer-by-layer polyelectrolyte multilayer films on quartz	Host-guest switch	UV 320–380 nm; visible >450 nm; dry and swollen films; UV-Vis and thermal Z→E kinetics	A photostationary state was reached after ~30 min UV irradiation (16% Z in the dry s-α-CyD film); the E-form recovered almost completely after 1 min visible light. S-α-CyD complexation modified photoisomerization and slowed thermal Z→E recovery	[15]
**Carbon nanotubes (MWCNTs, SWCNTs)**	Photoresponsive nanomaterial dispersion	Coumarin-modified water-soluble α-cyclodextrin (α-CD-C); Not reported; Photoresponsive α-CD-C/CNT supramolecular nanocomposite	Photoresponsive supramolecular scaffold	365 nm photodimerization and 254 nm photocleavage; UV-Vis/HPLC-MS; CNT dispersion monitoring	Coumarin photodimerization reversibly crosslinked the α-CD-C/CNT assembly at 365 nm, whereas 254 nm irradiation regenerated the monomeric state and altered CNT dispersion; the study addressed switching rather than guest photoprotection	[238]

Photoactive component / guest	Application	Cyclodextrin system / architecture	Role of CD	Light-related assessment	Main photoactive / photoresponsive outcome	Ref.
**Az-LPDM / Az-BPDM / plasmid DNA**	Photoresponsive gene delivery	poly(β-CD); CD:Az = 2:1; Supramolecular PCD/Az-polycation/DNA polyplexes	Host-guest release switch	365 nm UV; trans→cis conversion complete within ~15 min; NOESY after 30 min; DNA release/transfection compared ±UV	UV-induced Az isomerization disrupted PCD/Az host-guest complexes, reduced polyplex association and promoted DNA release; 15 min irradiation increased cellular transfection. No guest-photostability endpoint was measured	[16]
**Biotin and adamantane as competitive guests; doxorubicin as payload**	Light-activated tumor targeting and anticancer drug delivery	β-CD; Not directly determined; literature $K_a \approx 3 \times 10^2\ M^{-1}$ for biotin and $8.43 \times 10^3\ M^{-1}$ for adamantane; β-CD-capped mixed micelles containing o-nitrobenzyl groups and Dox	Competitive host-guest gate	365 nm UV cleavage of o-nitrobenzyl groups; surface-biotin exposure, uptake, release and cytotoxicity assessed ±UV	Photocleavage exposed adamantane, which displaced β-CD from biotin through stronger host-guest binding; exposed biotin increased from ~14.6% to 77.7% and cellular uptake by ~1.7-fold.	[199]
**AzoPEO / DOX**	Light-responsive graphene-based drug delivery	β-CD; Host–guest inclusion (AzoPEO/β-CD; stoichiometry not determined); β-CD-functionalized reduced graphene oxide (AzoPEO/β-CD-RGO)	Host-guest assembly switch	UV/visible-light reversible azobenzene isomerization; dispersion/aggregation and drug-release behavior monitored	AzoPEO/β-CD recognition reversibly controlled assembly of β-CD-functionalized RGO and light-responsive carrier behavior; no API photodegradation endpoint was measured	[239]

Photoactive component / guest	Application	Cyclodextrin system / architecture	Role of CD	Light-related assessment	Main photoactive / photoresponsive outcome	Ref.
**Azobenzene moieties and methylene blue**	Photoresponsive switching unit and model compound for controlled release	Acryloyl-modified α-CD covalently incorporated into the hydrogel; α-CD:Azo monomer feed ratio 1:4; Cross-linked PNIPAM-Azo/α-CD dual thermo- and photoresponsive hydrogel	Photoresponsive cavity gate	365 and 430 nm irradiation; UV-Vis photoisomerization and MB release; 20/37°C thermo-response	At 20 °C, MB release was 73.2% at 430 nm and 65.6% at 365 nm. Azo–α-CD association controlled cavity availability, while PNIPAM swelling/shrinkage supplied an independent thermal release mechanism	[240]
**4-Dodecylamino azobenzene and pendimethalin payload**	Photoresponsive switching unit and pre-emergence herbicide	γ-CD; 1:1 azobenzene:γ-CD by Job plot; pendimethalin was loaded non-stoichiometrically; Self-assembled γ-CD/azobenzene nanovesicles loaded with pendimethalin, dialysed and freeze-dried	Photoresponsive host–guest scaffold	365 nm UV or sunlight; azobenzene trans→cis switching, vesicle morphology and pendimethalin release; dark control	Pendimethalin release reached 88.3 ± 3% under UV/sunlight, 4.3-fold the dark-condition release (~20.5%); loaded vesicles ruptured after 5 min UV irradiation	[17]
**Coumarinylmethyl ester derivative**	Switchable photolabile crosslinker for polymer networks	Permethylated α-CD (PM α-CD); Intramolecular 1:1 pseudo[1]rotaxane architecture; Reversibly insulated/uninsulated coumarinylmethyl-ester pseudo[1]rotaxanes and crosslinked poly(NIPAM) gels	Molecular shielding / conformational gate	365 nm; 480 mW cm^{-2} for molecular systems and 33–116 mW cm^{-2} for gels; ^{1}H NMR and rheology	After 90 min, photodegradation was ~70% for the uninsulated form and ~20% for the PM α-CD-insulated form; supramolecular conformational switching converted polymer networks between photolabile and photostable states	[34]
Photodynamic, photothermal and photocatalytic systems						

Photoactive component / guest	Application	Cyclodextrin system / architecture	Role of CD	Light-related assessment	Main photoactive / photoresponsive outcome	Ref.
**Porphyrinoid photosensitizers, including pheophorbide *a* and tetra-tert-butylphenoxyphthalocyanine**	Photosensitizer delivery for targeted PDT	β-CD; functionalized and tethered β-CD dimers; biotinylated β-CD; 1:1 reported for simple monomeric CD-photosensitizer complexes; exact stoichiometry of dimeric clathrates not established; Monomeric photosensitizer-CD complexes and proposed high-affinity dimeric β-CD clathrates integrated with a biotin-avidin-anti-CEA targeting system	Photosensitizer solubilization / deaggregation	Visible absorption/disaggregation and binding studies; PDT activation proposed; no comparative photobleaching assay	β-CD and β-CD dimers solubilized/disaggregated hydrophobic porphyrinoid photosensitizers and enabled a proposed biotin–avidin targeting architecture; no direct CD-mediated photoprotection was quantified	[241]
**Pheophorbide a; octa-α-butyloxy-Zn-phthalocyanine; tetra-tert-butyl-Zn-naphthalocyanine**	Far-red photosensitizers for photodynamic therapy	Methylated β-CD, average methylation 1.8; γ-CD; Not established; complex formation inferred from spectral deaggregation and persistence upon dilution; Photosensitizer-CD systems prepared in PBS by adding EGMME/ethanol dye stock and heating for 5 min; CD concentrations up to 0.2 M	Photosensitizer deaggregation	UV-Vis/NIR absorption, steady/time-resolved fluorescence and $^1O_2$ luminescence under optical excitation; no photobleaching comparison	Methylated β-CD efficiently monomerized pheophorbide a and enabled $^1O_2$ generation in water; tetra-tert-butyl-Zn-naphthalocyanine required elevated temperature, whereas octa-α-butyloxy-Zn-phthalocyanine remained aggregated. γ-CD was less effective	[13]

Photoactive component / guest	Application	Cyclodextrin system / architecture	Role of CD	Light-related assessment	Main photoactive / photoresponsive outcome	Ref.
**Azobenzene groups as guests; protoporphyrin IX as photosensitizer**	Photoresponsive vesicles, singlet-oxygen generation and potential PDT	Di-β-CD-modified PPIX; 1:1:1 PPIX-2CD:AZO-HPG:AZO-HBPO preassembled unit; Hierarchically self-assembled PPIX-bridged Janus particles and hollow vesicles	Supramolecular organizer / vesicle scaffold	Xe-lamp (250 W) and UV-lamp (500 W) irradiation; UV-Vis kinetics, vesicle morphology and iodide $^1O_2$ assay	Free PPIX lost ~37% of Soret-band absorbance after 60 min Xe irradiation. Under 500 W UV, free PPIX lost ~25.2% after 10 min, whereas vesicle-bound PPIX lost ~8%; only 26% was degraded after 60 min. I_3^- conversion reached 86% after 60 min	[200]
**IR780-based [IR780][TPB] GUMBOS**	NIR fluorescent theranostic agent for tumor imaging and chemo/photothermal therapy	HP-β-CD; β-CD evaluated during optimization; 1:1 [IR780][TPB]:HP-β-CD preparation ratio was optimized; Core–shell CD-[IR780][TPB] nanoGUMBOS prepared by reprecipitation/self-assembly, ultrasonication and freeze-drying	Formulation / assembly component	Irradiation-time-dependent NIR fluorescence; free IR780 vs non-CD and HP-β-CD nanoGUMBOS; 808 nm PTT at 0.5 W cm^{-2} in solution and 2 W cm^{-2} in vivo	Free IR780 fluorescence decreased during irradiation, whereas both CD and non-CD nanoGUMBOS showed essentially unchanged emission maxima. CD nanoGUMBOS gave an ~18.0°C temperature rise after 10 min at 0.5 W cm^{-2}; the CD-specific photostability contribution was not isolated	[242]

Photoactive component / guest	Application	Cyclodextrin system / architecture	Role of CD	Light-related assessment	Main photoactive / photoresponsive outcome	Ref.
**Adamantane-functionalized SiO_2 as host-guest binding partner; ZnPc photosensitizers covalently conjugated with CD**	Singlet-oxygen photosensitizers for photooxygenation and photocatalytic pollutant degradation	Permethylated β-CD; two or four CD units covalently attached to ZnPc; Not determined; multivalent PM-β-CD-adamantane surface binding; not a classical ZnPc inclusion complex; Water-soluble $ZnPc(CD)_2$ and $ZnPc(CD)_4$ conjugates; supramolecular $1@SiO_2$ and $2@SiO_2$ nanosystems immobilized through CD-adamantane interactions	Supramolecular immobilization anchor	>610 nm irradiation; Q-band monitoring of free CD-ZnPc conjugates and SiO_2 -immobilized assemblies; photocatalysis and recycling	Free CD-ZnPc conjugates in water lost almost all Q-band absorbance after ~3 h, whereas $1@SiO_2$ and $2@SiO_2$ remained nearly unchanged after 6 h and retained recyclable photooxygenation/photocatalytic activity; stabilization arose from supramolecular immobilization on silica	[201]
**TMPyP cationic porphyrin**	Photosensitizer for antimicrobial photodynamic therapy	CAPTISOL® (SBE-β-CD, average DS ≈ 6.5); No discrete inclusion stoichiometry established; CAPTISOL®:TMPyP formulation ratio 50:1; Freeze-dried and reconstituted CAPTISOL®/TMPyP nanoassemblies	Solubilizing / assembly carrier	White LED, 54.82 J cm^{-2}, for aPDT/biofilm assays; spectroscopic and storage stability; no free-vs-CD photodegradation kinetics	CAPTISOL®/TMPyP nanoassemblies supported photoantimicrobial activity; biofilm destruction increased from ~68% to ~76%, but MIC/MBC were higher than for free TMPyP. Direct CD-mediated photoprotection was not demonstrated	[243]

Photoactive component / guest	Application	Cyclodextrin system / architecture	Role of CD	Light-related assessment	Main photoactive / photoresponsive outcome	Ref.
**Doxorubicin; Rhodamine B as thermosensitive probe**	Anticancer drug delivery, fluorescence imaging and photothermal therapy	β-CD grafted to PEI and functionalized with lactobionic acid; Not determined; PPy-RhB-PDA-CD-LA/DOX multifunctional nanoplatform	Carrier / assembly component	808 nm laser heating/cooling cycles, fluorescence monitoring and NIR-triggered DOX release	The multifunctional β-CD-containing platform converted NIR light to heat (photothermal conversion efficiency η = 51.7%) and triggered DOX release; no CD-specific guest-photoprotection comparison was performed	[244]
Fluorescent sensing and bioimaging systems						
**Dapoxyl sulfonic acid sodium salt**	Solvatochromic fluorescent probe for biological and supramolecular microenvironments	β-CD; 1:1; K_a ≈ 5488 M^{-1}; In situ aqueous inclusion complex	Microenvironmental host	Fluorescence measurements with 365 nm excitation; no comparative photobleaching kinetics	β-CD increased Dapoxyl fluorescence quantum yield from ~0.04 to ~0.31 and shifted emission from 584 to 560 nm, demonstrating strong microenvironmental control rather than photoprotection	[245]
**DEASPI two-photon dye and adamantane-labeled DEVD-BHQ2 peptide**	Two-photon fluorescent caspase-3 sensing and cellular/tissue imaging	β-CD-based polymer, βCDP; DEASPI/βCDP nanomicelles surface-functionalized with Ad-DEVD-BHQ2 through β-CD/adamantane recognition	Host-guest recognition scaffold	One- and two-photon fluorescence/FRET assays; NIR two-photon imaging in cells/tissues; no free-dye vs βCDP photobleaching study	DEASPI/βCDP nanomicelles enabled caspase-3-responsive two-photon imaging and reduced reliance on high-energy one-photon excitation; a CD-specific photoprotective effect was not quantified	[246]

Photoactive component / guest	Application	Cyclodextrin system / architecture	Role of CD	Light-related assessment	Main photoactive / photoresponsive outcome	Ref.
**Malachite green**	Aquaculture contaminant and analytical target	Amino-functionalized permethylated β-CD, OMe-β-CD; 1:1 MG/OMe-β-CD inclusion proposed; OMe-β-CD covalently conjugated to MPA-capped CdTe quantum dots	Analyte-recognition host	Continuous 365 nm irradiation for >1 h; QD fluorescence response monitored	OMe-β-CD/CdTe-QD probes retained >80% fluorescence and enabled malachite-green sensing; this reflects nanoprobe photostability rather than protection of malachite green	[247]
**Oleic-acid-coated $NaYF_4:Yb^{3+}/Er^{3+}$ nanoparticles with Rb-CO and RS-CO rhodamine chemosensors**	NIR-excited optical cysteine sensing	*α*-CD; Oleic acid/*α*-CD stoichiometry not established; Cys:chemosensor recognition was 1:1; *α*-CD-modified water-dispersible upconversion nanoparticles coupled with rhodamine sensors	Phase-transfer coating	Continuous 980 nm irradiation for 10 h; emission-intensity monitoring; comparison with conventional UV excitation	>93% of chemosensor emission was retained after 10 h under indirect 980 nm excitation. *α*-CD primarily enabled aqueous phase transfer of oleate-coated $NaYF_4:Yb^{3+}/Er^{3+}$ nanocrystals; photobleaching suppression was dominated by upconversion excitation	[248]
**Rhodamine chemosensors R-1 and R-2; cysteine as analyte**	NIR-excited upconversion optical sensing of cysteine	*α*-CD; *α*-CD-oleic-acid surface inclusion not quantified; chemosensor:Cys recognition 1:1; *α*-CD-phase-transferred $NaYF_4:Yb^{3+}/Er^{3+}$nanocrystals combined with rhodamine probes	Phase-transfer coating	Continuous 980 nm irradiation for 5 h; host and chemosensor emission monitored; comparison with direct UV excitation	>93% of chemosensor emission was retained after 5 h. Improved photostability arose mainly from indirect 980 nm excitation and the solid $NaYF_4$ host; *α*-CD served chiefly as the phase-transfer coating	[18]

Photoactive component / guest	Application	Cyclodextrin system / architecture	Role of CD	Light-related assessment	Main photoactive / photoresponsive outcome	Ref.
**Oleic-acid-coated $NaYF_4$:Yb,Er nanocrystals combined with rhodamine-derived chemosensors**	NIR-excited optical sensing of cysteine	*α*-CD; *α*-CD/oleic-acid stoichiometry not determined; chemosensor:Cys = 1:1; *α*-CD phase-transferred upconversion nanocrystals coupled with rhodamine sensors	Phase-transfer coating	Continuous 980 nm irradiation for 10 h; upconversion-host and chemosensor emissions monitored; direct-UV comparison	After 10 h, CD-$NaYF_4$ host emission retained 83–88% and chemosensor emission 68-70% of initial intensity; the composites outperformed direct UV excitation. *α*-CD mainly provided aqueous phase transfer rather than isolated photoprotection	[19]
**Lysozyme as analyte; cysteine-protected Cu nanoclusters as photoactive probe**	Photoluminescent biosensing of lysozyme in human serum	β-CD; non-classical surface host-analyte interaction; Water-soluble β-CD-stabilized cysteine-protected Cu nanoclusters	Nanocluster stabilizer	PL stability during 45 days storage plus pH/ionic-strength testing; no controlled irradiation comparison	β-CD-CuNCs increased absolute QY from 0.26% to 54.6%, showed a 12.7 ns fluorescence lifetime and lost <5% PL intensity after 45 days at room temperature; this is nanocluster stabilization, not classical guest photoprotection	[202]
**Dopamine**	Neurotransmitter and biomedical analytical target	β-CD capping/passivating groups on ZnO-doped carbon dots; binding constants ≈3428 M^{-1} for DA and ≈3125 M^{-1} for oxidized DA; β-CD-capped ZnO-doped fluorescent carbon dots prepared hydrothermally	Surface passivation / analyte recognition	Continuous 360 nm excitation for 2 h; fluorescence stability and dopamine-sensing response	β-CD-capped ZnO-doped carbon dots maintained stable blue fluorescence during irradiation and enabled dopamine sensing; dopamine itself was not assessed for CD-mediated photoprotection.	[249]

Photoactive component / guest	Application	Cyclodextrin system / architecture	Role of CD	Light-related assessment	Main photoactive / photoresponsive outcome	Ref.
**Rhodamine-derived chemosensors with cysteine as analyte; $NaYF_4:Yb^{3+}/Er^{3+}$ nanocrystals as excitation source**	NIR-excited optical detection of cysteine	α-CD coating on oleic-acid-stabilized upconverting nanocrystals; Not established for α-CD/UCNP assembly; 1:1 refers only to sensor–cysteine binding; Water-dispersible α-CD-modified $NaYF_4:Yb^{3+}/Er^{3+}$ UCNPs coupled with rhodamine-like sensors by energy transfer	Phase-transfer coating	980 nm upconversion excitation; photophysical/sensing performance and photobleaching resistance assessed without an α-CD-free photostability control	α-CD enabled aqueous phase transfer of oleate-coated UCNPs and supported off–on cysteine sensing (LOD 2.7-2.8 μM); reduced high-energy excitation, rather than isolated CD shielding, underpinned photostability	[250]
**No conventional guest; fluorescence generated by clustered nonconjugated N/O-containing groups**	Long-term cell tracking and in vivo bioimaging	β-CD covalently grafted onto PIMA; Not applicable-covalent β-CD-grafted polymer, not a discrete host-guest complex; self-assembled nonconjugated AIE-active PIMA-g-β-CD polymer nanoparticles	Polymer building block / self-assembly component	Aqueous fluorescence monitored for 30 days; dried 10 mg mL^{-1} films observed during 12 h illumination; cell/zebrafish imaging	Nonconjugated β-CD-grafted AIE polymer nanoparticles showed only slight fluorescence loss over 30 days and no obvious solid-state brightness loss after 12 h illumination; no separate CD-free polymer control isolated the β-CD contribution	[251]

Photoactive component / guest	Application	Cyclodextrin system / architecture	Role of CD	Light-related assessment	Main photoactive / photoresponsive outcome	Ref.
**Adamantyl Re-AdaQx-TPP complex and sodium deoxycholate as competitive guest**	Red AIPE luminophore and bile-acid sensing	β-CD; Re complex/β-CD approximately 1:1 expected from adamantane recognition; NaDC forms literature-reported 2:1 host:guest complexes; not determined experimentally here; β-CD-controlled competitive host–guest luminescence switching in acetonitrile/water	Competitive host–guest luminescence switch	385 nm irradiation for 24 h with UV-Vis monitoring; competitive host–guest luminescence switching	The Re luminophore was intrinsically photostable. β-CD suppressed aggregation-induced phosphorescence through adamantane inclusion, and sodium deoxycholate displacement restored red emission; the function was sensing/switching rather than photoprotection	[252]
Photostable nanomaterials and theranostic carriers						
**Silver nanoparticles, AgNPs**	Broad-spectrum antibacterial nanomaterial	β-CD used as a surface-capping agent; Not applicable-surface-capped nanoparticles; β-CD:$AgNO_3$ synthesis ratios of 2.5:1, 5:1 and 10:1; β-CD-capped AgNP colloids produced by $NaBH_4$ reduction	Surface-capping stabilizer	Xenon-chamber UV, 340 nm, 0.4 W m^{-2}, up to 4 h; surface-plasmon band monitored by UV-Vis	β-CD capping inhibited UV-induced AgNP growth/aggregation: uncapped particles darkened and the plasmon band broadened by ~14.05 nm, whereas higher-β-CD formulations changed only minimally.	[253]
Mechanistic photophysics and artificial photochemistry						

Photoactive component / guest	Application	Cyclodextrin system / architecture	Role of CD	Light-related assessment	Main photoactive / photoresponsive outcome	Ref.
**Indole or N,N-dimethylaniline; covalently attached thymine dimer/oxetane units**	Artificial photolyase model / photosensitized repair of thymine photolesions	Thymine-dimer-modified β-CD (TD-CD); thymine-oxetane-modified β-CD (Ox-CD); 1:1 for Ox-CD–DMA and Ox-CD–indole complexes; Photoactive supramolecular complexes of modified β-CD with electron-donor photosensitizers	Donor–acceptor supramolecular organizer	295 or 310 nm irradiation; quantum yields for thymine-dimer/oxetane splitting; host-guest binding and actinometry	β-CD inclusion organized electron-donor photosensitizers near covalently attached thymine lesions, enhanced photosensitized splitting and suppressed unproductive back electron transfer; the objective was artificial photorepair, not photoprotection	[201]
**Promazine and chlorpromazine hydrochlorides**	Phenothiazine drugs; chlorpromazine is associated with cutaneous photosensitization	HP-β-CD, molar substitution ≈0.60; 1:1; $K = 2.55 \times 10^3\ M^{-1}$ for PZ and $3.27 \times 10^3\ M^{-1}$ for CPZ; In situ aqueous inclusion complexes in PBS, pH 7.40	Excited-state microenvironment modifier	355 nm nanosecond laser-flash photolysis; transient-triplet kinetics and actinometry; no steady-state degradation assay	HP-β-CD increased phenothiazine triplet lifetimes and altered intersystem crossing. When >95% complexed, $\Phi T = 0.45 \pm 0.06$ for promazine and 0.17 ± 0.05 for chlorpromazine; no net photoprotection endpoint was quantified	[57]
